# E3 ubiquitin ligase HECTD2 mediates melanoma progression and immune evasion

**DOI:** 10.1038/s41388-021-01885-4

**Published:** 2021-06-18

**Authors:** Eleonora Ottina, Veera Panova, Laura Doglio, Anastasiya Kazachenka, Georgina Cornish, Joanna Kirkpatrick, Jan Attig, George R. Young, Kevin Litchfield, Tom Lesluyes, Peter Van Loo, Charles Swanton, James MacRae, Thomas Tüting, George Kassiotis

**Affiliations:** 1 Retroviral Immunology Laboratory, The Francis Crick Institute, London, UK; 2 Proteomics STP, The Francis Crick Institute, London, UK; 3 Retrovirus-Host Interactions Laboratory, The Francis Crick Institute, London, UK; 4 Cancer Evolution and Genome Instability Laboratory, The Francis Crick Institute, London, UK; 5 Cancer Genomics Laboratory, The Francis Crick Institute, London, UK; 6 Laboratory of Experimental Dermatology, Department of Dermatology, University of Magdeburg, Magdeburg, Germany; 7 Department of Infectious Disease, Faculty of Medicine, Imperial College London, London, UK

**Keywords:** Melanoma, Genetic markers

## Abstract

The ubiquitin-proteasome system maintains protein homoeostasis, underpins the cell cycle, and is dysregulated in cancer. However, the role of individual E3 ubiquitin ligases, which mediate the final step in ubiquitin-mediated proteolysis, remains incompletely understood. Identified through screening for cancer-specific endogenous retroviral transcripts, we show that the little-studied E3 ubiquitin ligase HECTD2 exerts dominant control of tumour progression in melanoma. HECTD2 cell autonomously drives the proliferation of human and murine melanoma cells by accelerating the cell cycle. HECTD2 additionally regulates cancer cell production of immune mediators, initiating multiple immune suppressive pathways, which include the cyclooxygenase 2 (COX2) pathway. Accordingly, higher HECTD2 expression is associated with weaker anti-tumour immunity and unfavourable outcome of PD-1 blockade in human melanoma and counteracts immunity against a model tumour antigen in murine melanoma. This central, multifaceted role of HECTD2 in cancer cell-autonomous proliferation and in immune evasion may provide a single target for a multipronged therapy of melanoma.

## Introduction

Cancer initiation and progression depends on the balance of multiple pro-tumour and anti-tumour processes. These include cancer cell-intrinsic mechanisms regulating cell proliferation, survival and migration [1, 2], and extrinsic factors, such as stromal cells in the tumour microenvironment and the anti-tumour immune response [3–6]. These processes are connected and a clear link between cancer cell-intrinsic genetic programs and anti-tumour immunity is beginning to emerge [7–9]. For example, defects in DNA damage repair and concomitant increase in mutation load or reactivation of endogenous retroviruses are thought to contribute to tumour immunogenicity by providing tumour-associated antigens [9–12]. In other instances, cancer cell cycle and proliferation programmes are under the control of transcription factors that also regulate the production of immune mediators, with NF-κB being a prime example [9]. Thus, cancer cell-intrinsic properties shape anti-tumour immunity, which in turn influences tumour evolution. However, the full extent of such bidirectional communication is incompletely understood and its outcome specific to the type of cancer [13].

Skin cutaneous melanoma (SKCM) is one of the most aggressive cancer types with increasing incidence worldwide [14]. It exhibits the highest load of somatic mutations among all cancer types [15] and can be inhibited by natural anti-tumour immunity and immunotherapy [16, 17]. Although intensely studied, the precise mechanisms underlying melanoma progression and evasion of natural and immunotherapy-induced immunity remain unclear. We recently examined the transcriptional activity of endogenous retroviruses in cancer and identified a novel transcript spanning the *HECTD2* locus and expressed uniquely in melanoma [18]. The novel transcript, *[HECTD2-AS]HERVH-2*, is transcribed antisense to *HECTD2* and uses a human endogenous retrovirus H (*HERVH*) provirus as terminal exon and polyadenylation signal [18]. Antisense transcription is mutually exclusive of sense transcription and strongly associated with a better prognosis of primary SKCM and uveal melanoma (UVM) [18]. These findings suggested a functional tumour-promoting role for HECTD2 in melanoma progression, which, however, remained unexplored.

HECTD2 is one of 28 members of the homologous to E6AP C-terminus (HECT) E3 ubiquitin ligases in humans [19]. Despite being essential for diverse cellular processes, most members of HECT E3 ubiquitin ligases, and HECTD2 in particular, are relatively understudied. *HECTD2* was first identified as a candidate susceptibility gene for prion and Alzheimer’s diseases [20, 21], but its substrates or mode of action remained unknown. A recent study identified PIAS1 (protein inhibitor of activated STAT-1) as a direct HECTD2 target [22]. PIAS1 is an E3 SUMO-protein ligase that negatively regulates key inflammatory pathways, including NF-κB and its targeting for degradation by HECTD2 is required for maximal NF-κB activation and innate immunity in the lung [22]. The role of HECTD2 in cancer has not yet been examined. However, two recent studies have suggested a possible involvement [23, 24]. *HECTD2* was identified as a candidate driver gene in neuroblastoma [24] or as a potential target of miR-221, which promotes androgen-independent growth of prostate cancer cell lines [23], thus indicating an anti-proliferative role for HECTD2 in this cancer type.

Here, we directly investigated the possible involvement of HECTD2 in melanoma, suggested by the discovery of the melanoma-specific *[HECTD2-AS]HERVH-2* antisense transcript and its association with better prognosis [18] and uncovered an unexpectedly central role.

## Results

### *HECTD2* expression defines transcriptional clusters in SKCM

**Fig. 1:**
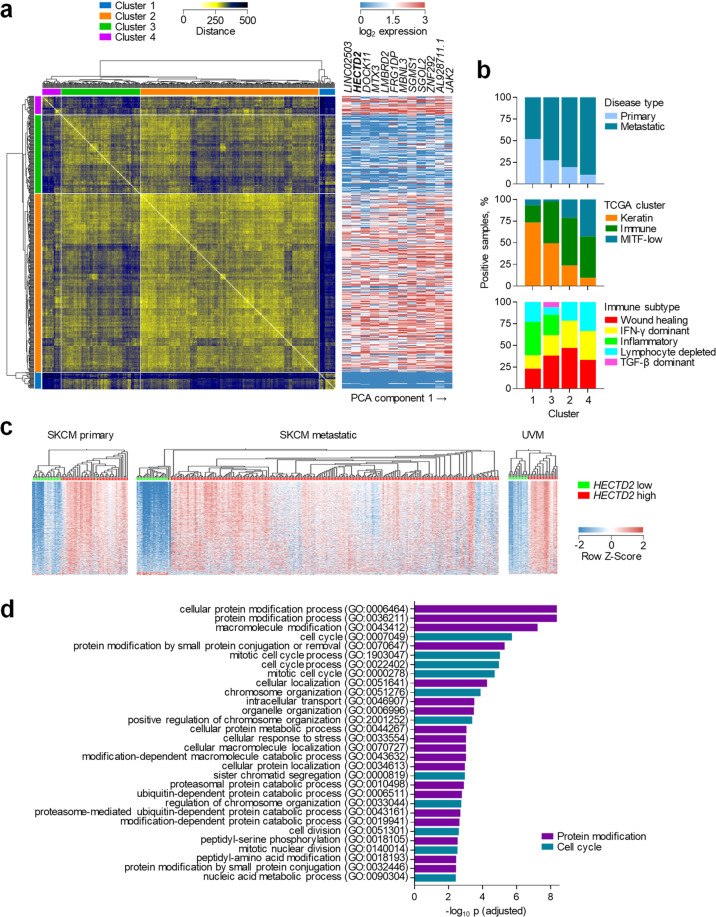
HECTD2 expression stratifies melanoma patients. **a** Hierarchical clustering of 442 TCGA SKCM samples according to sample distance, calculated based on all gene expression (left). Expression of the top 12 genes, ranked by their contribution to sample distance (right). Each row represents the same sample in both heatmaps. **b** Correspondence between each individual cluster defined by sample distance and previously described molecular clusters. **c** Hierarchical clustering of primary and metastatic SKCM samples and UVM samples, according to differential gene expression between low and high *HECTD2* expressing subsets (≥2-fold, *q* ≤ 0.05). **d** Gene functional annotation analysis of the top 1000 genes upregulated in high *HECTD2* expressing samples.

To investigate the basis for the association of *HECTD2* expression with SKCM survival, we first examined whether it marked disease subsets. Sample distance analysis of 443 SKCM samples from the cancer genome atlas [25] (TCGA) identified 4 distinct clusters based on the expression of all genes (Fig. 1a). Notably, *HECTD2* expression was second only to lncRNA *LINC02503* as the top gene responsible for the observed clustering (Fig. 1a). Clusters characterised by high *HECTD2* expression (2 and 4) were enriched for metastatic disease, where *HECTD2* expression is higher [18] (Fig. 1b). The TCGA-defined [25] ‘keratin’ subtype was underrepresented in clusters 2 and 4, whereas the ‘melanocyte inducing transcription factor (MITF)-low’ subtype was almost exclusive to these high *HECTD2*-expressing clusters (Fig. 1b). For samples where ‘immune landscape’ annotation was available [26], the ‘inflammatory’ subtype was underrepresented in clusters 2 and 4, whereas the ‘IFN-γ dominant’ and ‘lymphocyte depleted’ subsets enriched (Fig. 1b). Thus, clusters identified by sample transcriptional distance and characterised by distinct *HECTD2* expression levels correlated well with previously identified molecular and immune subtypes [25, 26].

To further probe the potential influence of *HECTD2* expression on the observed transcriptional profiles, we filtered genes differentially expressed between subsets with high (≥2 transcripts per million, TPM) or low expression of *HECTD2* (Fig. 1c). This analysis identified over 6000 differentially expressed genes (≥2-fold, *q* ≤ 0.05) in primary SKCM, metastatic SKCM and UVM separately, the majority of which (5166) were differentially expressed in all three conditions (Fig. 1c). Moreover, nearly all of these genes were upregulated in high *HECTD2*-expressing samples in all three conditions (Fig. 1c). Lastly, functional enrichment analysis of the top 1000 genes upregulated in *HECTD2*-expressing samples identified two major pathways, protein modification (including ubiquitin-dependent protein degradation) and cell cycle (Fig. 1d). Together, these data raised the possibility that *HECTD2* expression was part, if not a driver, of the extensive transcriptional dichotomy of melanoma.

### HECTD2 cell-autonomously promotes melanoma cell proliferation

**Fig. 2:**
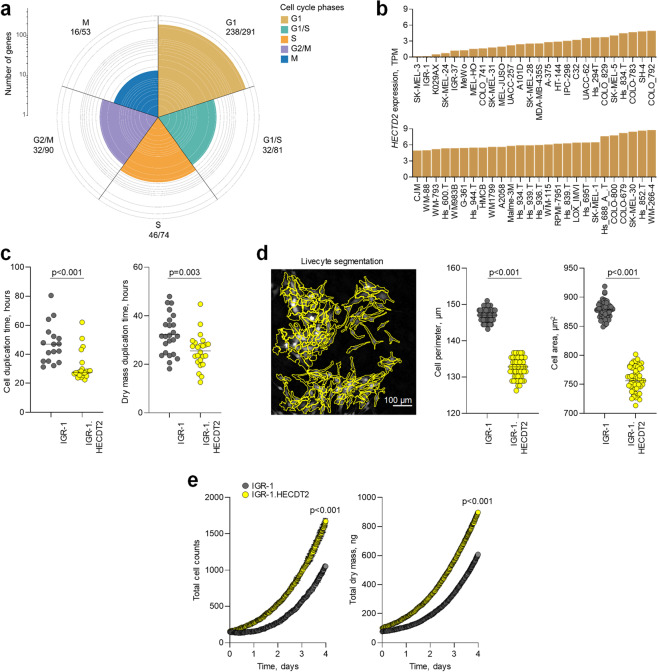
HECTD2 expression drives the cell cycle in human melanoma cell lines. **a** Distribution of genes differentially upregulated in high *HECTD2* expressing samples, according to the phase of the cell cycle they are functionally annotated to be involved in. **b** Expression of *HECTD2* (TPM) in the indicated human melanoma cells lines in data from CCLE. **c** Cell duplication times (left) and dry mass duplication times (right) of parental IGR-1 human melanoma cells and IGR-1 cells overexpressing *HECTD2* (IGR-1.HECTD2). Symbols represent the mean values of separate wells (*n* = 24), with 4 fields of view averaged per well, from three independent experiments. **d** Representative example of cell perimeter and cell area demarcation using Livecyte segmentation (left) and cell perimeters and cell areas of IGR-1 and IGR-1.HECTD2 cells. Symbols represent individual cells in one of three independent experiments. **e** Total cell counts (left) and total dry mass (right) in cultures of IGR-1 and IGR-1.HECTD2 cells over time. Plotted are the mean values (±SEM) of the mean of each of the three independent experiments. *P* values were calculated with Mann–Whitney Rank Sum tests.

Whereas induction of genes involved in protein modification and degradation was expected in samples expressing high levels of *HECTD2*, induction of cell cycle-related genes was not. Despite its essential role in maximal NF-κB activation [22], HECTD2 has not been previously associated with the cell cycle and the only currently available data suggest an anti-proliferative role for HECTD2 in androgen-independent growth of the LNCaP prostate cancer cell line [23]. We, therefore, investigated if the apparent induction of cell cycle genes in melanoma biopsies with high *HECTD2* expression could be due to a cell-autonomous proliferative effect of HECTD2. The upregulated genes in melanoma biopsies covered all stages of the cell cycle but were particularly enriched for the G1 phase, indicating a stronger effect on mRNA and protein synthesis (Fig. 2a). Consistent with melanoma biopsies, the vast majority of human melanoma cell lines from the cancer cell line encyclopaedia [27] (CCLE) expressed medium to high *HECTD2* levels, with the exception of SK-MEL-3 and IGR-1 cells, where *HECTD2* levels were very low (Fig. 2b). Inspection of cancer dependency map (DepMap) data [28] indicated that *HECTD2* is not an essential gene in cell lines from multiple cancer types, including melanoma (Fig. S1a). Although the loss of *HECTD2* function was variable and overall neutral for cell lines derived from metastatic melanoma, a potentially negative effect on growth was observed for lines derived from primary melanoma, in proportion with *HECTD2* expression (Fig. 1a). To examine a direct effect on cell growth, we stably overexpressed HECTD2 in IGR-1 cells by retroviral transduction (Fig. S1b). Compared with parental cells, IGR-1 cells overexpressing HECTD2 (IGR-1.HECTD2) exhibited decreased cell and dry mass duplication times and considerably reduced cell perimeter and area (Fig. 2c, b), consistent with accelerated proliferation, which was further confirmed by analysis of total cell counts and total dry mass accumulated over time (Fig. 2e), and with live imaging of cell growth (Video S1).

**Fig. 3:**
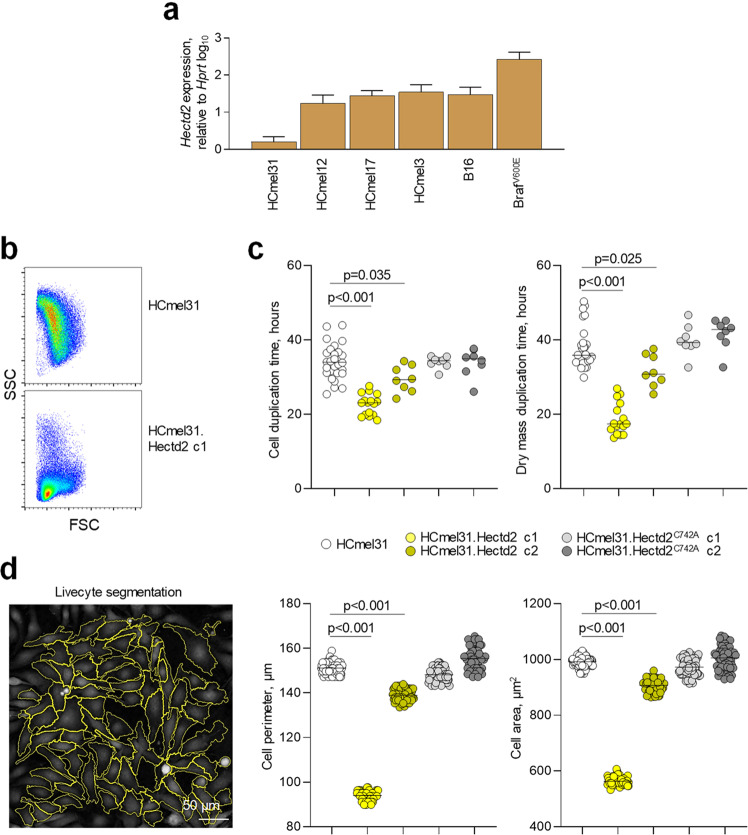
HECTD2 expression drives the cell cycle in murine melanoma cell lines. **a** Expression of *Hectd2* in the indicated murine melanoma cells lines, measured by RT-qPCR. **b** Flow cytometric example of forwarding and side scatters of parental HCmel31 murine melanoma cells and HCmel31 clone 1 cell overexpressing *Hectd2* (HCmel31.Hectd2 c1). **c** Cell duplication times (left) and dry mass duplication times (right) of parental HCmel31 and HCmel31 cells overexpressing wt or C742A HECTD2 variant proteins (two clones for each). Symbols represent the mean values of separate wells (*n* = 8–28), with four fields of view averaged per well, from 1 to 4 independent experiments. **d** Representative example of cell perimeter and cell area demarcation using Livecyte segmentation (left) and cell perimeters and cell areas of the same HCmel31 cell line derivatives. Symbols represent individual cells in one of three independent experiments. *P* values were calculated with One Way Analysis of Variance (ANOVA) on Ranks tests.

To extend these findings, we examined the potential role of HECTD2 in murine melanoma. The murine and human HECTD2 proteins share 89.3% amino acid identity, indicating a highly conserved function in the two species (Fig. S2a). Moreover, *Hectd2* was highly expressed in commonly used murine melanoma cells lines, with the exception of HCmel31 cells, where it was expressed at much lower levels, determined by RT-qPCR (Fig. 3a). Similar to the human *HECTD2* locus, the murine *Hectd2* locus demonstrates both sense and antisense transcription, initiated at syntenic positions (Fig. S2b). However, in contrast to the human antisense transcript, which terminates at the human-specific *HERVH* integration, the murine antisense transcript, *Hectd2os* does not span the *Hectd2*-encoding locus and, importantly, is not anti-correlated with sense transcription (Fig. S2b, c). Thus, although the regulation of its transcription differs between mouse and man, HECTD2 expression characterises both human and murine melanoma cell lines.

To examine a direct effect on cell growth, we stably overexpressed murine HECTD2 in HCmel31 cells by retroviral transduction (Fig. S3a–c), together with a GFP reporter separated by an internal ribosome entry site. Two clones were selected, expressing slightly different *HECTD2* levels (Fig. S3a–c). As a control, we also overexpressed a C742A mutant of murine HECTD2 with a predicted loss of the catalytic activity (Fig. S3b; Fig. S4; Supplementary text).

In keeping with its effects on human IGR-1 cells, overexpression of HECTD2 in murine HCmel31 cells had a dramatic effect on cell size and granularity, as determined by flow cytometry, decreased the cell and dry mass duplication times and significantly reduced cell perimeter and area, as determined by microscopy (Fig. 3b–d), and accelerated cell growth, as determined by live imaging (Video S2). The observed effects were stronger in HCmel31.Hectd clone 1, expressing higher HECTD2 levels, but were not seen in cells expressing the C742A HECTD2 variant (HCmel31.Hectd^C742A^), regardless of expression levels (Figs. 3b–d; S3; Video S2).

To further quantify the proliferative advantage conferred by HECTD2 expression, we competed for parental HCmel31 cells and HECTD2-overexpressing HCmel31.Hectd cells, which were distinguished by GFP expression in the latter (Fig. S5a). The ratio of HCmel31.Hectd cells to HCmel31 cells changed over 15-fold during a 3-day co-culture of cells plated at equal starting numbers, and a comparable shift was seen for co-cultures started at different ratios (Fig. S5a). In contrast, the ratio of HCmel31.Hectd^C742A^ cells to HCmel31 cells remained constant in their respective co-cultures for at least 7 days (Fig. S5a).

We used the same competition assay to examine the effect of HECTD2 overexpression in murine melanoma cells lines B16 and Braf^V600E^, which were already spontaneously expressing high levels of HECTD2. Further increasing HECTD2 expression B16 and Braf^V600E^ cells had no measurable effect on their growth, when competed with the respective parental cell lines (Fig. S5c, d), likely because HECTD2 expression in these cells was already mediating the maximal effect. We, therefore, performed the reverse experiment of loss of HECTD2 function in Braf^V600E^ cells. To this end, we used Cas9-mediated disruption of the *Hectd2* gene in these cells by introducing a promoterless GFP-encoding open reading frame into *Hectd2* exon 1 (Fig. S6). Expression of GFP in these cells reports insertion in the correct position with respect to the locus promoter (Fig. S6). Targeted cells were sorted on the basis of GFP expression to over 90% purity, but their frequency was reduced to under 60% after a two-week culture (Fig. S6b). GFP^+^ cells were sorted again to a higher purity (99%), but again were outcompeted by the residual GFP^−^ cells following culture (Fig. S6b), and a stable GFP^+^ subline could not be established. Thus, high spontaneous expression of HECTD2 in Braf^V600E^ cells appeared necessary for maximal cell growth in vitro.

**Fig. 4:**
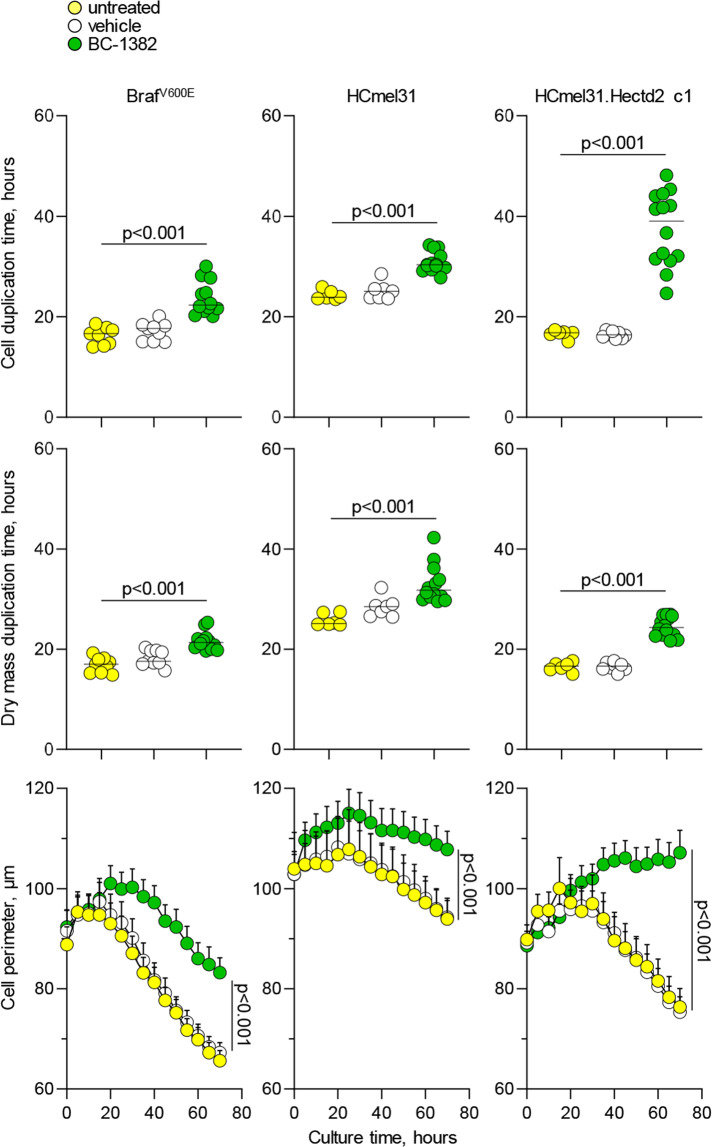
Deceleration of cell proliferation by in vitro HECTD2 inhibition. In vitro growth of parental Braf^V600E^ cells, HCmel31 cells and *Hectd2*-overexpressing HCmel31.Hectd2 c1 cells, in the absence of treatment or in the presence of the 200 µM BC-1382 inhibitor or of the DMSO (vehicle) alone. Mean cell duplication times (top) and dry mass duplication times (middle) of separate wells (*n* = 9–12), with four fields of view averaged per well, are shown from 3 to 4 independent experiments. *P* values were calculated with ANOVA on Ranks tests. Mean cell perimeters (±SEM) from separate wells (*n* = 9–12) are shown (bottom) from three independent experiments. *P* values were calculated Student’s *t*-tests.

To independently confirm these results, we attempted to block HECTD2 activity using the recently developed BC-1382 small molecule inhibitor [22]. In cultures of Braf^V600E^ and HCmel31 cells, the addition of BC-1382 significantly increased cell and dry mass duplication times, as well as cell perimeter over time (Fig. 4). These changes were equivalent to a 4.8-fold and 2.0-fold reduction in cell accumulation over 5 days of culture, for Braf^V600E^ and HCmel31 cells, respectively. The magnitude of this effect, at least for HCmel31 cells, was not different from the non-specific effect of BC-1382 on HECTD2-negative U937 cells (1.9-fold reduction; Fig. S7; Supplementary text) and could not, therefore, be attributed to HECTD2 inhibition. In contrast, the addition of BC-1382 to HCmel31.Hectd c1 cells had a much stronger effect on the same parameters, equating to a 16.3-fold reduction in cell accumulation in the same time period, fully reversing the effects of HECTD2 overexpression in these cells (Fig. 4). Collectively, these results support a major role for catalytically-active HECTD2 in driving the cell-autonomous proliferation of human and murine melanoma cell lines.

### HECTD2 tunes immune-reactivity to melanoma growth

Although HECTD2 clearly promoted in vitro growth of melanoma cell lines, in vivo tumour growth is influenced by additional tumour-extrinsic factors, including the anti-tumour immune response. A pro-inflammatory role for HECTD2 in the healthy lung has been proposed [22] and it was, therefore, possible that high HECTD2 expression in melanoma was also pro-inflammatory.

**Fig. 5:**
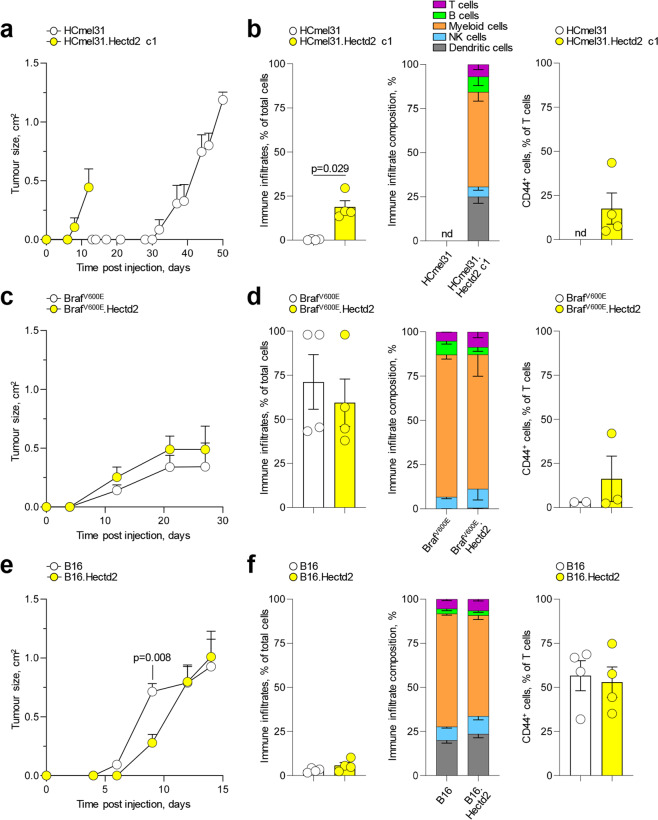
Effect of HECTD2 overexpression on murine melanoma growth in vivo. **a** Mean size (±SEM) of tumours formed over time following injection of HCmel31 cells or HCmel31.Hectd2 c1 cells into B6 recipients (*n* = 4 and *n* = 4, respectively, from one of two experiments). **b** Percentage and composition of CD45^+^ immune infiltrates, and percentage of antigen-experienced CD44^+^ cells in tumour-infiltrating T cells in the same recipients as in a. Bars graphs denote the mean (±SEM) and symbols represent individual mice. **c** Mean size (±SEM) of tumours formed over time following injection of Braf^V600E^ cells or Braf^V600E^.Hectd2 cells into B6 recipients (*n* = 8 and *n* = 8, respectively, from two experiments). **d** Percentage and composition of CD45^+^ immune infiltrate, and percentage of antigen-experienced CD44^+^ cells in tumour-infiltrating T cells in the same recipients as in c. Bars graphs denote the mean (±SEM) and symbols represent individual mice. **e** Mean size (±SEM) of tumours formed over time following injection of B16 cells or B16.Hectd2 cells into B6 recipients (*n* = 4 and *n* = 4, respectively, from one of two experiments). **f** Percentage and composition of CD45^+^ immune infiltrates, and percentage of antigen-experienced CD44^+^ cells in tumour-infiltrating T cells in the same recipients as in e. Bars graphs denote the mean (±SEM) and symbols represent individual mice.

To examine the overall effect of HECTD2 expression on melanoma growth in vivo, we employed the three transplantable murine cell lines, HCmel31, Braf^V600E^ and B16, and monitored tumour formation and infiltration by diverse immune cell types (Fig. S8). HCmel31 cells transplanted into fully-syngeneic C57BL6/J (B6) mice grew into tumours over a period of 30–50 days, without eliciting an overt immune reaction (Fig. 5a, b), as previously reported [29, 30]. In stark contrast, the growth of HCmel31.Hectd cells transplanted into such recipients was significantly accelerated (7–14 days) and was also accompanied by immune infiltration of the tumours (Fig. 5a, b). Immune infiltrates comprised diverse immune cell types, dominated by myeloid cells, but contained relatively few antigen-experienced (CD44^high^) T cells (Figs. 5b and S8). Accordingly, despite the recruitment of immune cells in the HCmel31.Hectd tumours, their growth was unaffected by an adaptive immune response, as comparably accelerated growth of HCmel31.Hectd cells were observed also in severely immunodeficient *Rag1*^−*/*−^*Il2rg*^−*/*−^*Cd47*^−*/*−^ recipients, lacking all lymphocytes (Fig. S9), demonstrating a tumour cell-intrinsic effect of HECTD2 expression. Therefore, overexpression of HECTD2 in HCmel31 cells autonomously increased their in vivo growth and ignited immune infiltration of tumours formed, which, however, did not restrain their growth.

As previously reported [29–33], Braf^V600E^ cells injected into B6 mice formed tumours with faster kinetics than HCmel31 cells (Fig. 5c). Moreover, Braf^V600E^ tumours were heavily infiltrated by immune cells, predominantly myeloid cells, often outnumbering tumour cells, with very few antigen-experienced CD44^high^ T cells or dendritic cells (Fig. 5c). HECTD2 overexpression in Braf^V600E^ cells had no measurable effect on either growth or immune infiltration (Fig. 5c, d). Similarly, injection of B16 cells into B6 mice led to rapid tumour growth and immune infiltration by low overall numbers of immune cells, but containing a higher proportion of antigen-experienced CD44^high^ T cells and dendritic cells (Fig. 5e, f). HECTD2 overexpression in B16 cells caused a slight delay (1–2 days) in tumour growth but did not appreciably alter the outcome of immune response to the rumours (Fig. 5e, f). Thus, consistent with in vitro data, in vivo growth of HCmel31, Braf^V600E^ and B16 cells correlated with their spontaneous HECTD2 expression and was dramatically increased by HECTD2 overexpression specifically in HCmel31 where HECTD2 was not already highly expressed. Similarly, HECTD2 overexpression increased the immune infiltration of otherwise immune-depleted HCmel31 tumours but did not further enhance the infiltration of more immunogenic Braf^V600E^ and B16 tumours.

**Fig. 6:**
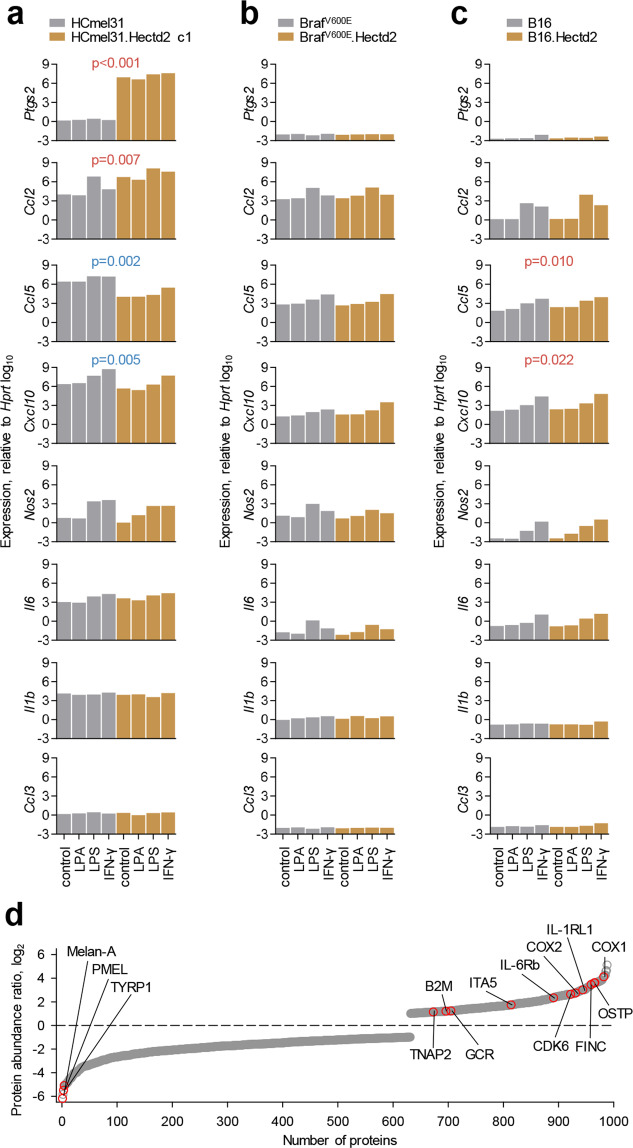
Effect of HECTD2 overexpression on in vitro melanoma cell expression of NF-κB target genes encoding pro- and anti-inflammatory mediators. **a**–**c**, Production of the indicated mediators, assessed by RT-qPCR in parental HCmel31 (**a**), Braf^V600E^ (**b**) and B16 cells (**c**) and the respective *Hectd2*-overexpressing derivatives, either spontaneously or following stimulation with LPA, LPS or IFN-γ. Bars denote the means of technical RT-qPCR triplicates. *P* values are calculated by paired comparisons between parental and *Hectd2*-overexpressing sublines for each treatment condition (Student’s paired *t*-test). P values in red and blue indicate significant upregulation and downregulation, respectively, in *Hectd2*-overexpressing sublines. **d** Ratio of protein abundance between HCmel31.Hectd2 c1 and parental HCmel31 cells for the 990 individual proteins that were found to be differentially abundant (≥2-fold, *p* ≤ 0.05, *q* ≤ 0.05) between the two cell lines, ranked according to their ratio.

The combination of these findings suggested that a potential anti-tumour immune response promoted by the pro-inflammatory activity of HECTD2 was either non-functional or counteracted by a stronger immune-suppressive activity. To explore these possibilities, we measured the transcription levels of immune mediators that could be cell-intrinsically correlated with HECTD2 activity. Given the recently described role for HECTD2 in potentiating NF-κB activity in response to innate immune stimuli [22], we included in our analysis NF-κB target genes and also stimulation with lipopolysaccharide (LPS), lysophosphatidic acid (LPA) and IFN-γ, which activate NF-κB. Overexpression of HECTD2 in HCmel31 cells caused a marked increase (over one million-fold) in the transcription of *Ptgs2*, the gene encoding cyclooxygenase 2 (COX2), also known as prostaglandin G/H synthase 2 (PGHS2), independently of innate immune stimulation (Fig. 6a). Additionally, HECTD2 overexpression in HCmel31 cells significantly increased transcription levels of *Ccl2*, encoding CCL2, also known as monocyte chemoattractant protein-1 (MCP-1) (Fig. 6a). *Ccl2* transcription was responsive to LPS and IFN-γ stimulation, but the enhancing effect of HECTD2 overexpression was evident both in stimulated and unstimulated cells (Fig. 6a). Transcription levels of *Ccl5* and *Cxcl10* were also responsive to LPS and IFN-γ stimulation in HCmel31 cells, but, in contrast to levels of *Ccl2*, they were significantly reduced by HECTD2 overexpression both in stimulated and unstimulated cells (Fig. 6a). *Nos2* and *Il6* transcription responded to LPS and IFN-γ stimulation, but remained unaffected by HECTD2 overexpression, whereas *Il1b* and *Ccl3* transcription was minimally affected by innate immune stimulation or HECTD2 overexpression in HCmel31 cells (Fig. 6a). In agreement with the differential effects on growth or in vivo immune infiltration of the three murine cell lines, a further expression of HECTD2 did not affect transcription of these immune mediators in Braf^V600E^ cells and only moderately increase *Ccl5* and *Cxcl10* transcription in B16 cells (Fig. 6b, c), again illustrating the different melanoma profiles exemplified by these three cell lines.

To independently, as well as more comprehensively, validate the observed effects of HECTD2 overexpression on NF-κB target genes, we compared the global proteomic profiles of parental and HECTD2-overexpressing HCmel31 cells. HECTD2 overexpression leads to loss of melanosome-specific proteins Melan-A, PMEL and TYRP1, and to the significant overrepresentation of several known NF-κB targets, including COX2 (also known as PGHS2, encoded by the *Ptgs2* gene) and its homologue COX1 (also known as PGHS1, encoded by the *Ptgs1* gene), upregulated 6.7-fold and 17.2-fold, respectively (Fig. 6d; Table S1; Fig. S10; Supplementary text). Collectively, these data confirm the direct link between HECTD2 overexpression and increased NF-κB activity, which likely underlies the effect of HECTD2 on the cell cycle and immunogenicity of tumour cells.

### HECTD2 counteracts adaptive immune resistance to melanoma

The results from the murine HCmel31 cells demonstrated that HECTD2 overexpression modified the balance of the pro-inflammatory and immune-suppressive mediators we analysed. To examine if this effect on the selected immune mediators reflected broader changes in immune signatures and also in human melanoma, we build modules of co-regulated immune genes.

**Fig. 7:**
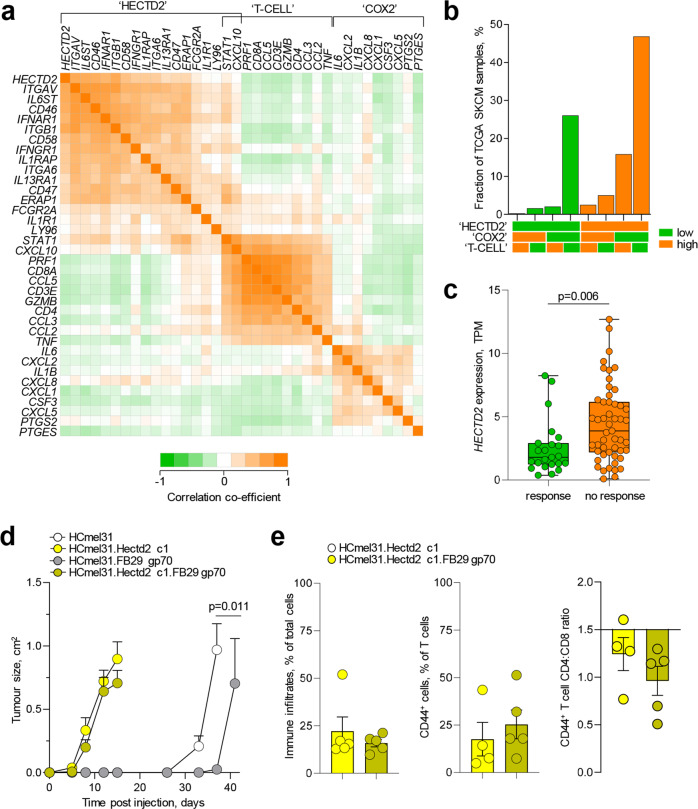
Effect of HECTD2 expression on human melanoma immunotherapy and murine melanoma antitumour immunity. **a** Correlation matrix of expression levels of genes associated with the ‘HECTD2’, ‘T-CELL’ and ‘COX2’ modules in 442 TCGA SKCM samples. **b** Percentage of 442 TCGA SKCM samples that express the indicated combination of the ‘HECTD2’, ‘T-CELL’ and ‘COX2’ gene modules. **c** *HECTD2* expression (TPM) in melanoma biopsies from patients exhibiting a response (*n* = 23) or no response (*n* = 51) to anti-PD-1 immunotherapy (*p* value calculated with Mann–Whitney Rank Sum test). Box plots show the upper and lower quartiles, centre lines show the median and whiskers represent the 1.5x interquartile range. Symbols show all individual samples. **d** Mean size (±SEM) of tumours formed over time following injection into B6 recipients of HCmel31 cells or HCmel31.Hectd2 c1 cells and derivatives of these engineered to express FB29 gp70, (*n* = 4, *n* = 7, *n* = 3 and *n* = 7, respectively). **e** Percentage of CD45^+^ immune infiltrates, percentage of antigen-experienced CD44^+^ cells in tumour-infiltrating T cells and CD4:CD8 ratio in antigen-experienced tumour-infiltrating T cells in the same recipients as in d. Bars graphs denote the mean (±SEM) and symbols represent individual mice. The CD4:CD8 ratio was significantly lower (*p* = 0.002), Student’s *t*-test in antigen-experienced T cells infiltrating HCmel31.Hectd2 c1.FB29 gp70 tumours than in naïve B6 recipients.

In TCGA SKCM, expression of *PTGS2* was highly correlated with expression of *PTGES*, encoding prostaglandin E synthase, in a module we refer to as ‘COX2’ to denote the COX2-mediated prostaglandin E_2_ (PGE_2_) biosynthetic pathway (Fig. 7a). In agreement with prior reports [33], this module was characterised by higher expression of *IL6*, *IL1B* and *CSF3*, as well as of several neutrophil chemoattractants (Fig. 7a). Also agreeing with prior reports [33], the ‘COX2’ module was distinct from the ‘T-CELL’ module, characterised by expression of genes from CD4^+^ and cytotoxic CD8^+^ T cells and chemokine genes *CCL2*, *CCL3*, *CCL5* and *CXCL10* (Fig. 7a).

Despite the stronger association with protein modification and cell cycle pathways (Fig. 1d), high expression of HECTD2 in TCGA SKCM was also associated with a number of co-regulated immune genes (Fig. 7a). These formed a separate module, referred to as ‘HECTD2’, which was distinct from the ‘COX2’ module and showed only a small overlap (*STAT1*, *CXCL10*) with the ‘T-CELL’ module (Fig. 7a). The upregulated genes in the ‘HECTD2’ module encoded predominantly transmembrane proteins, such as integrins and adhesion molecules (*ITGAV*, *ITGB1*, *ITGA6* and *CD58*), and receptors and co-receptors for interleukins (*IL1R1*, *IL6ST*, *IL13RA1*), interferons (*IFNAR1* and *IFNGR1*), immunoglobulins (*FCGR2A*), complement (*CD46*) and LPS (*LY96*) (Fig. 7a). Also notable was the co-expression in the ‘HECTD2’ module of *CD47* (Fig. 7a), which encodes CD47, also known as integrin associated protein (IAP), and which can inhibit phagocytosis in macrophages [34].

We next stratified 442 TCGA SKCM samples according to the aggregate expression of the genes in each of these three distinct modules and looked for the overlap in expression. As expected by the segregation observed at the gene level (Fig. 7a), the expression of the modules in SKCM samples also largely segregated into distinguishable subsets (Fig. 7b). The ‘COX2’ module was expressed in a minority of SKCM samples (41 of 442, 9.3%), most of which were within the ‘HECTD2’-expressing subset (Fig. 7b). Samples expressing the ‘T-CELL’ module were more numerous (91 or 442, 20.6%), largely distinct from ‘COX2’-expressing samples, and most were also within the ‘HECTD2’-expressing subset (Fig. 7b). However, the largest single subset, amounting to nearly half of all samples (207 of 442, 46.8%), expressed the ‘HECTD2’ module in the absence of the other two modules (Fig. 7b).

The partial overlap of the ‘HECTD2’ and ‘T-CELL’ modules at the gene expression level (Fig. 7a) and inclusion of the majority of the ‘T-CELL’-expressing samples within the ‘HECTD2’-expressing subset (Fig. 7a; *p* < 0.00001, Fisher Exact test) is consistent with a pro-inflammatory role for HECTD2. However, the much larger number of ‘HECTD2’-expressing samples without evidence of an anti-tumour T cell response, suggests either lack of inherent immunogenicity of these tumours or, alternatively, active HECTD2-mediated immune modulation, only a small part of which could be attributed to the immune suppressive ‘COX’ module. To examine if elevated HECTD2 expression may correlate with a lack of effective T cell anti-tumour immunity, we analysed cohorts of melanoma patients that were treated with PD-1 blocking antibodies [35, 36]. Notably, the outcome of such immunotherapy correlated significantly with levels of HECTD2 expression (Fig. 7c). Given that the response to PD-1 blockade was not strongly associated with tumour mutation load and, by extension, expected tumour neoantigenicity in either of these cohorts [35, 36], the link between elevated HECTD2 expression and lack of immunotherapy response suggest that HECTD2 activity may counteract T cell anti-tumour immunity.

To test this hypothesis directly, we used the non-immunogenic HCmel31 tumour cells, which we engineered to express a retroviral antigen (Friend helper murine leukaemic virus isolate FB29 envelope glycoprotein gp70), previously shown to elicit adaptive anti-tumour responses even in the absence of prior immunisation [30, 37]. The expression of FB29 gp70 in HCmel31 cells significantly delayed their growth after inoculation into naïve B6 mice (Fig. 7d). In contrast, expression of FB29 gp70 had no discernible impact on the growth of HCmel31 cells that expressed high levels of HECTD2, despite the recruitment of sizeable immune infiltrates, particularly of CD8^+^ T cells, mediated by HECTD2 (Fig. 7d, e). Thus, although HECTD2 expression alone was sufficient to attract immune cells to the otherwise non-immunogenic HCmel31 tumours, it did not promote T cell-mediated tumour resistance and even negated the resistance provided by the immunogenic expression of FB29 gp70 as a tumour-specific antigen.

## Discussion

Our results place HECTD2 as a central regulator of a number of different functions that determine melanoma progression. This central role is supported by the stratification of melanoma subtypes based primarily on HECTD2 expression, forming transcriptional clusters of genes involved in protein modification and the cell cycle. Accordingly, HECTD2 expression directly promoted cell-autonomous proliferation of human melanoma cells in vitro and murine melanoma cells in vitro and in vivo. This increase in proliferation was also accompanied by extensive morphological changes at least in vitro, with a reduction in cell size as the most notable.

Another major effect of HECTD2 activity in melanoma cells is on their production of soluble immune mediators and transmembrane proteins involved in interaction with immune cells. HECTD2 overexpression in HCmel31 cells directly increased levels of *Ptgs2* transcription by at least six orders of magnitude. Cancer cell-intrinsic expression of COX2, encoded by *Ptgs2*, has been shown to induce PGE_2_, which in turn subverts myeloid cell function in melanoma [33]. In contrast, loss or inhibition of COX2 activity promotes anti-tumour immunity, developing either spontaneous or in the context of checkpoint blockade [33]. Its effect on *Ptgs2* transcription places HECTD2 upstream of this important ‘COX2’ module, which controls the PGE_2_ biosynthetic pathway, as well as expression of *IL6*, *IL1B*, *CSF3* and neutrophil chemoattractants. Induction of the immunosuppressive ‘COX2’ module by HECTD2 offers one explanation for the negative effect on anti-tumour immunity we observed in this study. However, the ‘COX2’ module is found only in a minority of SKCM samples (9.3%), predominantly also expressing high levels of HECTD2 and lack evidence for T cell infiltration. In contrast, nearly half of SKCM samples expressed the ‘HECTD2’ module and were devoid of T cell infiltration, without expressing the ‘COX2’ module. These findings suggest that HECTD2 activity is responsible for COX2-mediated immunosuppression, but may also activate more prevalent alternative immunosuppressive mechanisms, such as the balance of cytokines and chemokines.

Using data from two separate studies [35, 36], we found that higher expression of HECTD2 in melanoma patients was predictive of a poor response to PD-1 blockade. Moreover, overexpression of HECTD2 in a mouse melanoma model diminished the effectiveness of the adaptive immune response to a model tumour antigen. These observations support an immunosuppressive role for HECTD2 in melanoma, which warrants further investigation. It is notable that the ‘HECTD2’ module is characterised by immune-related genes encoding predominantly transmembrane proteins, including CD47. By binding to its receptor SIRPα (signal receptor protein-alpha) on macrophages, CD47, ubiquitously expressed in healthy or transformed cells, inhibits their phagocytosis by macrophages, by sending ‘do not eat me’ signals [34, 38]. Elevated CD47 expression in cancer cells counteracts their pro-phagocytic signals and CD47 blockade is considered a valid target for immunotherapy [34, 38]. Collectively, these findings suggest an association of HECTD2 expression with multiple immune evasion pathways.

HECTD2 is widely expressed in healthy tissues, including the skin [18]. We find that this expression is maintained in primary melanoma and further elevated in metastatic melanoma [18], supporting a link between high HECTD2 expression and melanoma progression. However, several mechanisms that regulate HECTD2 expression and activity have been proposed, operating at distinct levels, as would be expected for genes involved in multiple cellular processes. A possible role for HECTD2 in melanoma was suggested by its regulation through the recently discovered *[HECTD2-AS]HERVH-2* antisense transcript expressed highly specifically in melanoma [18]. Antisense transcription has long been recognised as an effective regulator of gene activity [39] and appears to be the predominant form of *HECTD2* regulation in human melanoma [18]. Other than melanoma, antisense *[HECTD2-AS]HERVH* transcripts have been detected in bladder adenocarcinoma, as well as healthy bladder and a few reproductive tissues [18]. In most healthy tissues and cancer types, however, *HECTD2* expression is not subject to regulation by antisense transcription.

*HECTD2* expression has also been shown to be directly regulated by miR-221 in prostate cancer cells [23]. MiR-221 and miR-222 are two small non-coding RNAs with the same seed sequence that have been implicated in cancer [40] and their targeting of *HECTD2* could extend its involvement beyond melanoma. *HECTD2* expression may additionally be regulated by the lncRNA ERP in human cancer, as has been suggested by studies in epithelial cells [41, 42].

Lastly, in addition to transcription and stability of the *HECTD2* RNA, Coon et al. reported high frequency (8.5%) of a single-nucleotide polymorphism affecting HECTD2 activity [22]. However, our independent analysis of all available genomes failed to detect this variant, excluding a contribution to the regulation of HECTD2 activity (Supplementary text).

Collectively, our results point to a critical role for HECTD2 in promoting melanoma cell-intrinsic proliferation and drug resistance and counteracting anti-tumour adaptive immunity and immunotherapy. Blocking this multifaceted melanoma-promoting function of HECTD2 may, therefore, be considered as a potential treatment of melanoma.

## Methods

### Mice

Inbred C57BL/6J (B6) and severely immunodeficient *Rag1*^−*/*−^*Il2rg*^−*/*−^*Cd47*^−*/*−^ mice were originally obtained from The Jackson Laboratory (Bar Harbor, ME, USA) and subsequently maintained at the Francis Crick Institute’s animal facilities. Eight to twelve-week-old male and female gender-matched recipient mice were used for all experiments, randomly allocated to the different groups. All animal experiments were approved by the ethical committee of the Francis Crick Institute and conducted according to local guidelines and UK Home Office regulations under the Animals Scientific Procedures Act 1986 (ASPA).

### Cell lines, transfection and transduction

HCmel cell lines [29, 32] and Braf^V600E^ cells [31] have been previously described. Detailed information about cell lines, transfection and transduction are available in Supplementary materials and methods.

### Statistical analysis

Statistical comparisons were made using SigmaPlot 13.0 (Systat Software Inc., Germany). Parametric comparisons of normally distributed values that satisfied the variance criteria were made by unpaired Student’s *t*-tests or One Way Analysis of variance (ANOVA) tests. Data that did not pass the variance test were compared with non-parametric two-tailed Mann–Whitney Rank Sum tests or ANOVA on Ranks tests.

Detailed information about the materials and methods used in the present study is available in Supplementary materials and methods.

## Supplementary information

Supplementary text

Supplementary materials and methods

Supplementary figures

Table S1

Video S1

Video S2
