# Supplementary material for: E3 ubiquitin ligase HECTD2 mediates melanoma progression and immune evasion
Source: Oncogene. 2021 Jun 18;40(37):5567–78. doi: 10.1038/s41388-021-01885-4 (PMC8445817; doi:10.1038/s41388-021-01885-4)
Supplement: Supplementary file 1 — Supplementary text [file 41388_2021_1885_MOESM1_ESM.pdf]

Supplementary text

Catalytically inactive HECTD2

The catalytic cysteine residue was previously mapped in the catalytic HECT domain of human E6AP E3 ubiquitin ligase [1] and alignment of HECT domains of several related E3 ubiquitin ligases confirmed the conservation of this cysteine residue (position 742 of murine HECTD2), in agreement with PROSITE feature annotation as the active site (Fig. S4). This cysteine residue was therefore replaced with an alanine residue to create a catalytically-inactive C742A HECTD2 variant [1], and the C742A HECTD2 variant was overexpressed also in HCmel31 cells.

Specificity of the BC-1382 inhibitor

As the specificity of this inhibitor has not been fully established in prior studies, we first tested it on cells that did not express HECTD2. To this end, we used the monocytic U937 cell line, one of very few human cell lines that do not express detectable *HECTD2* RNA (Fig. S7a). U937 cells were chosen as we have been unable to establish a HECTD2-deficient melanoma cell line or achieve sufficient and stable knock-down of *HECTD2* expression using shRNA approaches. The addition of BC-1382, but not the vehicle, significantly reduced the accumulation of U937 cells by half (1.9-fold reduction) over 5 days of culture (Fig. S7b), consistent with a non-specific effect.

Proteomics analyses of HECTD2 overexpression

A total of 990 of detectable proteins differed in their abundance between HCmel31 and HCmel31.Hectd2 c1 cells (≥2-fold, $p \leq 0.05$, $q \leq 0.05$) (Table S1), distinguishing the two cell types with high confidence (Fig. S10a). Two thirds of these proteins were downregulated upon HECTD2 overexpression and one third were upregulated (Fig. 6d). In addition to the cell cycle, the differentially abundant proteins were involved in protein/organelle biogenesis, subcellular organisation and cell

morphology/motility (Fig. S10b), in agreement with analysis of RNA-seq data. More specifically, HECTD2 overexpression lead to the relative loss of melanosome-specific proteins Melan-A, PMEL and TYRP1, which were 3 of the top 4 downregulated proteins (Fig. 6d; Table S1). Upregulated proteins included COX2 (also known as PGHS2, encoded by the *Ptgs2* gene) and its homologue COX1 (also known as PGHS1, encoded by the *Ptgs1* gene), both of which are known NF-κB targets, upregulated 6.7-fold and 17.2-fold, respectively (Fig. 6d; Table S1), confirming data on *Ptgs2* transcription. They also included numerous other NF-κB targets with immune function, such as the cytokine osteopontin (OSTP); cytokine receptors IL-1RL1 and IL-6Rb; β_2 microglobulin (B2M), a component of MHC class I complex; adhesion molecules fibronectin (FINC) and integrin α_5 (ITA5); the glucocorticoid receptor (GCR, encoded by the *Nr3c1* gene); tumour necrosis factor α-induced protein 2 (TNAP2); and the cell cycle regulator cyclin-dependent kinase 6 (CDK6) (Fig. 6d; Table S1).

Single-nucleotide polymorphism affecting HECTD2 activity

Coon et al. reported the existence of naturally occurring single-nucleotide polymorphism affecting HECTD2 activity [2]. This nonsynonymous G/C polymorphism (rs7081569) causes an A19P substitution in the protein sequence and loss of HECTD2 function due to mislocalisation to the cytosol [2]. Importantly, Coon et al. reported a C allele frequency of 8.5% in the human population [2], which could have important implications for melanoma progression too. However, despite C being the reference allele in the current assembly of the human genome, none of the 5,008 samples in the 1,000 Genomes Project (https://www.ncbi.nlm.nih.gov/snp/rs7081569) and none of the germline cases in the Pan-Cancer Analysis of Whole Genomes (PCAWG) project harboured a C allele and were all homozygous G at this SNP location. We therefore used the $HECTD2^{A19}$ variant throughout the study and conclude that the C allele is extremely rare in the human population and cannot be a major contributor to the regulation of HECTD2 activity.

References

1. Huang L, Kinnucan E, Wang G, Beaudenon S, Howley PM, Huibregtse JM et al. Structure of an E6AP-UbcH7 complex: insights into ubiquitination by the E2-E3 enzyme cascade. Science. 1999; 286: 1321-6.

2. Coon TA, McKelvey AC, Lear T, Rajbhandari S, Dunn SR, Connelly W et al. The proinflammatory role of HECTD2 in innate immunity and experimental lung injury. Sci Transl Med. 2015; 7: 295ra109.
