# Supplementary material for: E3 ubiquitin ligase HECTD2 mediates melanoma progression and immune evasion
Source: Oncogene. 2021 Jun 18;40(37):5567–78. doi: 10.1038/s41388-021-01885-4 (PMC8445817; doi:10.1038/s41388-021-01885-4)
Supplement: Supplementary file 2 — Supplementary materials and methods [file 41388_2021_1885_MOESM2_ESM.pdf]

Supplementary materials and methods

Cell lines, transfection and transduction

B6-derived B16-F0 cells (CRL-6322) cells were obtained from ATCC. HCmel3, HCmel12, HCmel17 and HCmel31 melanoma cells were established independently from primary 7,12-dimethyl-1,2-benzanthracene (DMBA)-induced melanomas in individual HGF-CDK4(R24C) mice [1, 2]. Melanoma cells expressing the *Braf*^{V600E} form were originally established from mice expressing an inducible *Braf*^{V600E} allele specifically in melanocytes [3] and were kindly provided by Dr. Richard Marais at the University of Manchester (Manchester, UK) through Dr. Caetano Reis e Sousa at the Francis Crick Institute. HEK293T and U937 cells were originally obtained from ATCC. IGR-1 cells were kindly provided by Julian Downward at the Francis Crick Institute. EL4 (TIB-39) cells were kindly provided by Drs. Brigitta Stockinger, at the Francis Crick Institute. All cells were grown in Iscove's Modified Dulbecco's Medium (IMDM) (Sigma-Aldrich, St. Louis, MO, USA) supplemented with 10% fetal bovine serum (Gibco, Thermo Fisher Scientific, Waltham, MA USA), 2 mM L-glutamine, penicillin (100 U/mL) and streptomycin (0.1 mg/mL) and non-essential amino acids (Gibco). Melanoma lines expressing HECTD2 or its mutant were generated by transduction with VSVg-pseudotyped viral particles harbouring Flag-tagged HECTD2 or HECTD2^{C743A}. Cells were FACS-purified by GFP expression twice, 72 hours and one week post transduction, on the Avalon, Propel Labs/Bio-Rad. Braf^{V600E} HECTD2-deficient cells were generated by transfection with the px459 plasmids containing guide RNAs 268 or 271 and 382 or 383 and the repair template which was generated via PCR using the pUC57 plasmid described above with the RANGER DNA polymerase (Meridian Bioscence) and the following primers: forward 5'-GATCTGTCCATCTGTAACTTAT-3', reverse 5'-GTGTGTCATTGCTGTCCAAAT-3'. Before transfection the amplicon was purified with the QIAquick PCR purification kit (Qiagen). After 48 hours post transfection the cells underwent 1 µg/ml puromycin selection for three days before FACS sorting the cells for GFP expression on the Avalon. HCmel31 and HCmel31.Hectd2 cells expressing FB29 were generated by

transduction with VSVg-pseudotyped viral particles harbouring FB29, and after 72 hours and one week post transduction were FACS sorted for envelope expression using the F-MuLV Env gp70-specific mAb 720 on the Avalon. All murine cell lines were verified for their origin and were mycoplasma free. IGR-1, HEK293T and U937 human cancer cell lines underwent an authentication process which included species identification and STR profiling performed at the Cell Services facility at the Francis Crick Institute. Certain cell lines were additionally treated for the indicated times with 200 µM of the HECTD2 inhibitor BC-1382 (Sigma-Aldrich). Cell growth was additionally assessed using an AlamarBlue (Invitrogen)-based assay, according to manufacturer's instructions. AlamarBlue fluorescence was read with a TECAN Spark plate reader (TECAN) and was plotted as arbitrary fluorescence units. All the transplantable cancer lines were passaged for a maximum 18-24 times or kept for a maximum of 8 weeks in culture. To monitor the levels of inflammatory mediators in melanoma upon HECTD2 overexpression, one million melanoma cells were plated for 24-48 hours in 6-well plates in 2 ml of culture medium supplemented or not with 10 µg/ml LPS, 0.5 µM LPA, 20 ng/ml TNF-α, or 100 U/ml IFN-γ. 48 hours post treatments the cells were collected and the levels of canonical and non-canonical NF-κB targets were measured via RT-qPCR, using the following primers:

Target gene	Forward primer sequence (5' – 3')	Reverse primer sequence (5' – 3')
Hprt	TTGTATACCTAATCATTATGCCGAG	CATCTCGAGCAAGTCTTTCA
Ptgs2	CAGGCAGCAAATCCTTGCTG	AAGTGGGTCAGGATGTAGTGC
Ccl2	AGCGCCATATGGAGCTGAC	TTCGAGTGACAAACACGACTG
Ccl5	ATCTCTGCAGCTGCCCTCA	AAAGCAGCAGGGAGTGGTG
Cxcl10	TCAAGCCATGGTCCTGAGACAA	CGCACCTCCACATAGCTTACAG
Nos2	CAGCTGGGCTGTACAAACCTT	CATTGGAAGTGAAGCGTTTCG
Il6	GAGAAAAGAGTTGTGCAATGGC	TCCAGGTAGCTATGGTACTCC
Il1b	AAAAGATGAAGGGCTGCTTCC	GTCCACGGGAAAGACACAGG
Ccl3	GCTGTAGTTTTTGTCACCAAGC	GCATTAGCTTCAGATTTACGGG

Gene synthesis, cloning and mutagenesis

Gene encoding flag-tagged murine or human HECTD2 were synthesised and cloned into pRV-IRES-GFP vector upstream of the internal ribosome entry site (IRES) sequence. The C743A mutation was introduced the murine gene using QuickChange site-directed mutagenesis kit (Agilent). The F-MLV clone FB29 envelope gene was synthesised and cloned into pRV-IRES-GFP vector. All gene synthesis, cloning and mutagenesis were performed by Genewiz LLC. All of the resulting constructs were verified by sequencing. Vesicular stomatitis virus glycoprotein (VSVg)-pseudotyped retroviral particles were produced by transfection, using GeneJuice (EMD Millipore), of 3 µg of either flag-tagged WT HECTD2 or $HECTD2^{C743A}$, or FB29 vector plasmids, together with 3 µg packaging (pHIT60) and 3 µg VSVg-expressing (pcVG-wt) plasmids into 3×10^{5} HEK293T cells. Virus-containing supernatants were collected 48 hours post transfection, passed through a 0.45-mm filter and stored at –80°C until further use. For the generation of HECTD2 defective cells via CRISPR-Cas9, the px459 vectors expressing the guides 268 (5'-GCGCCGGCCGTCACGATGGGCGG-3') or 271 (5'-GCTGCGCCGGCCGTCACGATGGG-3') or 383 (5'-ACAGGGAAGTTGCGCCTCGAGGG-3') or 382 (5'-AACAGGGAAGTTGCGCCTCGAGG-3') were synthesized by Genewiz LLC. For the generation of the repair template the sequence encoding for the first 34 amino acids of HECTD2 followed in frame by GFP and the first intron of HECTD2 was synthetized and cloned into the pUC57 AmpR vector by Genewiz LLC. All the sequences were verified by sequencing.

Flow cytometry and cell sorting

Single-cell suspensions were prepared from freshly isolated tumours and lymph nodes following digestion with 100 µg/ml Liberase TL (Roche, Mannheim, Germany) for 30 min at 37°C. Flow cytometry analysis was performed with the following monoclonal antibodies: RA3-6B2, anti-B220; H57-597, anti-TCRβ; GK1.5, anti-CD4; 53-6.7, anti-CD8; IM7, anti-CD44; M5/114.152, anti MHCII; M1/70 anti-CD11b; N418, anti-CD11c; RB6-8C5, anti–Gr-1; A20, anti-CD45.1; 104, anti-CD45.2; purchased from either eBioscience (San Diego, CA, USA), BioLegend (San Diego, CA, USA) or BD Biosciences (San Jose, CA,

USA). Dead cells were identified by staining with Propidium iodide. Samples were analysed on an LSRFortessa X20 cell analyser. Tumour and host cell sorting was performed on a BD FACSAria Fusion (BD Biosciences) or MoFlo XDP (Beckman Coulter, Brea, CA, USA) cell sorters.

Live cell imaging

Live cell imaging and quantitative analysis of cell growth and morphology were performed label-free on the Livecyte system (Phasefocus). Ten thousand melanoma cells in 0.5-2 ml medium were seeded per well in a 24-well plate 18 hours prior to imaging. Four regions of interest were selected for each well using 10× magnification and imaged with 5 minutes intervals. Images were acquired using a 10× Plan N objective and Livecyte Acquire software. Single-cell tracking, segmentation and analysis were performed using Livecyte Analyse software.

Protein modelling

The model of the putative HECT domain of murine HECTD2 (NP_001156943.1) was done with SWISS-MODEL at ExPASy using the E6AP HECT catalytic domain (1d5f.1.B) as template. The prediction scored 0.76 GMQE, 0.33 QSQE and 48.84% identity.

Tumour challenge

Tumour challenge was initiated by subcutaneous inoculation of tumour cell suspensions. One million parental or HECTD2-expressing HCmel31 cells were inoculated into the right flank of B6 hosts, which were monitored three times per week and up to 60 days. One million or half a million parental or HECTD2-expressing B16F0 cells were inoculated into the right flank of B6 hosts, which were monitored three times per week and up to 15 days. Half a million BrafV600E cells were injected into the right flank of B6 host, which were monitored three times per week and up to 30 days. Tumour sizes (cm^2) were determined by calliper measurements of two axes and calculated using the formula: $\pi \times a \times b$,

where *a* = half of length and *b* = half of width of the tumours. These measurements were confirmed by additional calliper measurements and weighing of the resected tumour mass at the end of the observation period. Investigators were not blinded to the different groups.

Gene and protein functional annotation

Pathway analyses were performed using *g:Profiler* web server [4] (https://biit.cs.ut.ee/gprofiler) with genes ordered by the degree of differential expression. *P* values were estimated by hypergeometric distribution tests and adjusted by multiple testing correction using the g:SCS (set counts and sizes) algorithm, integral to the *g:Profiler* server.

Cell line gene dependency analysis

Analysis of gene expression and essentiality in tumour cells lines was performed on the Cancer Dependency Map [5] (DepMap) portal website (https://depmap.org/portal).

Western blotting

Heat denatured (95°C for 10 min) cell lysates were run on a 4-20% gel (Biorad), transferred to a PVDF membrane (Biorad), and blocked in 5% (w/v) bovine serum albumin fraction V (Sigma-Aldrich). Human and murine HECTD2 were detected by incubation with a recombinant rabbit monoclonal anti-HECTD2 antibody (EPR11554) (Abcam, #ab173572), which reacts with HECTD2 from both species. Membranes were developed with secondary HRP-conjugated goat antiserum to rabbit IgG (Abcam, #ab6721). ACTIN was detected with an HRP-conjugated mouse monoclonal anti-β-actin antibody (Abcam, #ab49900). Blots were visualized by chemiluminescence on an Amersham Imager 600 (GE Healthcare).

Proteomics analyses

Five replicate cultures of at least 10^6 cells each of HCmel31 or HCmel31.Hectd2 c1 cells were processed for label-free proteomic analyses. Cell pellets were resuspended with pipetting, first in 50 µl ice-cold PBS, then a 2× lysis buffer was added (also 50 µl). Final lysis buffer concentration was 4 % SDS/HEPES pH 8 100mM/TCEP 20mM. Cells were then lysed using Ultrasonic probe (for 30 seconds) on ice. 10x Chloroacetamide (CAA) was added to final concentration of 40mM. Protein lysates reduced and alkylated simultaneously (70°C/10 min). Cell debris spun down (20,000×*g*/5min/RT). 50 µl (expected 50 µg) was removed into fresh tube and precipitated overnight with 8× volume of ice-cold acetone (at -20°C). Pellets were washed twice (with 80% ice-cold acetone/500 µl) after initial centrifugation (30mins/4°C/20,000×*g*). Pellets were centrifuged again between washes (10mins/4°C/20,000×*g*) and after being left to air-dry after last wash removed, resuspended in digest buffer (1M GuaHCl/100mM HEPES, pH 8). LysC enzyme (Wako) was added (0.5 µg) and digestion proceeded at 37°C with 1,400 rpm shaking for 3 hours. Samples were diluted 1:1 with water, and trypsin (Pierce) added (1 µg). Digestion continued overnight at 37°C/700 rpm).

Samples were then acidified (pH 2, with formic acid, 2 µl). Approximately 1 µg of each digested sample was loaded onto Evotips (as prepared according to manufacturer's instructions) and washed once with aqueous acidic buffer (0.1% formic acid in water) before loading onto the Evosep One system in front of the Orbitrap Fusion Lumos (Thermo). The Evosep One was fitted with a 15 cm column and the predefined method for a 44-minute method was employed. Data for all samples was acquired in Data Independent Acquisition mode (DIA). A pool of each condition made for duplicate data acquisition by DDA. DIA Lumos settings were as follows: Transfer capillary set to 300°C and 2.2kV applied to the nanospray needle (Evosep). MS1 data acquired in the Orbitrap with a resolution of 120k, max injection time of 20 ms, AGC target of 10^6, in positive ion mode, in profile mode, over the mass range 393-907 *m/z*. DIA segments over this mass range (20 *m/z* wide/1 Da overlap/27 in total) were acquired in the Orbitrap following fragmentation in the HCD cell (32%), with 30k resolution over the mass range 200-2,000 *m/z* and with a max injection time of 54 ms and AGC target of 10^6. DDA data used the same

source settings with the following MS method changes: MS1 resolution = 60k, charge state inclusion 2-6$^+$, MIPS mode (Peptide), dynamic exclusion of 15 s, intensity threshold of 5×10^4, DDA carried out with quadrupole isolation of 1.4 Da, HCD energy of 32%, MS2 acquired in the Orbitrap with 15k resolution, max injection time of 22 ms, AGC target of 10^6 in centroid mode. Data (DDA and DIA) were searched together using the Pulsar search engine inside Spectronaut software (Biognosys AG) to make a combined library. Data were searched against the Uniprot Proteome Database for *Mus Musculus (uniprot-proteome_UP000000589.fasta)* and a database of common contaminants. The DIA data were then searched against this library. The library contained 24,977 precursors that correspond to 18,854 peptides from 2,982 Protein Groups. Differential protein expression was carried out inside Spectronaut software to compare HCmel31.Hectd2 c1 versus parental HCmel31 cell differences. Data visualisation of exported candidate and report tables carried out in R-Studio. Raw data were uploaded onto the Proteomics Identification Database (PRIDE) (https://www.ebi.ac.uk/pride) under accession number: PXD025237.

RNA-seq data analysis

RNA-seq reads were obtained from TCGA, CCLE, and the two we cohorts of melanoma patients that were treated with PD-1 blocking antibodies [6, 7] (Accession numbers GSE91061 and GSE78220). Reads were aligned to the GENCODE assembly (basic, version 30) or our custom transcript assembly, as previously described [8]. Transcripts per million (TPM) calculations were carried out for all transcripts with a custom Bash pipeline using GNU parallel [9] and Salmon [10] (v0.12.0). TPM values were transformed with the *log()* function (R v3.6.3) and used for sample clustering. Euclidean distances between the samples were calculated using the *dist()* function (R v3.6.3). A "pheatmap" library was used to plot the distances. Sample clusters were defined using the *cutree()* function. All downstream differential expression analyses and visualisation were carried out using Qlucore Omics Explorer 3.3 (Qlucore, Lund, Sweden).

DNA-seq data analysis

We used alleleCounter (https://github.com/cancerit/alleleCount; v4.0.0) to obtain nucleotide counts at SNP rs7081569 (chr10:93170250, hg19 coordinates) for all germline cases part of the PCAWG (Pan-Cancer Analysis of Whole Genomes; 2,658 samples) cohort. At MAPQ=35 and BASEQ=20 thresholds (alleleCounter default values), 2,092 samples (79%) had more than 5 counts. Of those, 2061 samples only contained reads with the G nucleotide (VAF_G=1). The remaining 31 samples had a median VAF_G of 0.96 (minimum was 0.86), with no sample showing more than one read containing for A, C and T, strongly suggesting that these were sequencing errors. With relaxed thresholds (MAPQ=0 and BASEQ=0), 2,189 samples (82%) had more than 5 counts, including 1,848 samples with VAF_G=1. Median VAF_G for the remaining 341 samples was 0.96, and counts for A, C and T did not exceed 4. Of note, coverage at this location was globally only half of the mean read coverage reported for normal samples in PCAWG (39X), likely due to a very high GC content around this SNP (77% in a +/- 125bp window), with a flagged (RepeatMasker) region due to high G/C repeats adjacent to this locus.

References

1. Bald T, Quast T, Landsberg J, Rogava M, Glodde N, Lopez-Ramos D et al. Ultraviolet-radiation-induced inflammation promotes angiotropism and metastasis in melanoma. Nature. 2014; 507: 109-13.

2. Landsberg J, Kohlmeyer J, Renn M, Bald T, Rogava M, Cron M et al. Melanomas resist T-cell therapy through inflammation-induced reversible dedifferentiation. Nature. 2012; 490: 412-6.

3. Dhomen N, Reis-Filho JS, da Rocha Dias S, Hayward R, Savage K, Delmas V et al. Oncogenic Braf induces melanocyte senescence and melanoma in mice. Cancer Cell. 2009; 15: 294-303.

4. Raudvere U, Kolberg L, Kuzmin I, Arak T, Adler P, Peterson H et al. g:Profiler: a web server for functional enrichment analysis and conversions of gene lists (2019 update). Nucleic Acids Res. 2019; 47: W191-w98.

5. Tsherniak A, Vazquez F, Montgomery PG, Weir BA, Kryukov G, Cowley GS et al. Defining a Cancer Dependency Map. Cell. 2017; 170: 564-76.e16.

6. Hugo W, Zaretsky JM, Sun L, Song C, Moreno BH, Hu-Lieskovan S et al. Genomic and Transcriptomic Features of Response to Anti-PD-1 Therapy in Metastatic Melanoma. Cell. 2016; 165: 35-44.

7. Riaz N, Havel JJ, Makarov V, Desrichard A, Urba WJ, Sims JS et al. Tumor and Microenvironment Evolution during Immunotherapy with Nivolumab. Cell. 2017; 171: 934-49.e16.

8. Attig J, Young GR, Hosie L, Perkins D, Encheva-Yokoya V, Stoye JP et al. LTR retroelement expansion of the human cancer transcriptome and immunopeptidome revealed by de novo transcript assembly. Genome Res. 2019; 29: 1578-90.

9. Tange O. GNU Parallel: The Command-Line Power Tool. The USENIX Magazine. 2011; 36: 42-47.

10. Patro R, Duggal G, Love MI, Irizarry RA, Kingsford C. Salmon provides fast and bias-aware quantification of transcript expression. Nat Methods. 2017; 14: 417-19.
