# Supplementary material for: E3 ubiquitin ligase HECTD2 mediates melanoma progression and immune evasion
Source: Oncogene. 2021 Jun 18;40(37):5567–78. doi: 10.1038/s41388-021-01885-4 (PMC8445817; doi:10.1038/s41388-021-01885-4)
Supplement: Supplementary file 3 — Supplementary figures [file 41388_2021_1885_MOESM3_ESM.pdf]

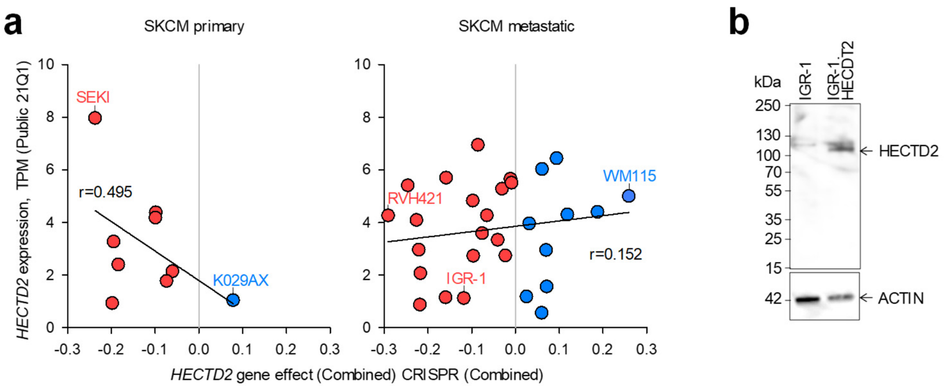

Figure S1. *HECTD2* expression and essentiality in human melanoma cell lines. a, The effect of *HECTD2* loss of function (through Cas9-mediated loss) is plotted against *HECTTD2* expression for SKCM cells lines available on the DeMap portal. Each symbol is a different cell line and cell lines derived from primary and metastatic SKCM are plotted separately. Blue and red symbols represent positive and negative gene effects, respectively. IGR-1 cells and cell lines at the extremes of the spectrum are also marked. **b**, Western blotting of HECTD2 in parental IGR-1 cells and IGR-1 cells overexpressing HECTD2 (IGR-1.HECTD2). Blotting for ACTIN is also included as a loading control. The blots are from a single experiment.

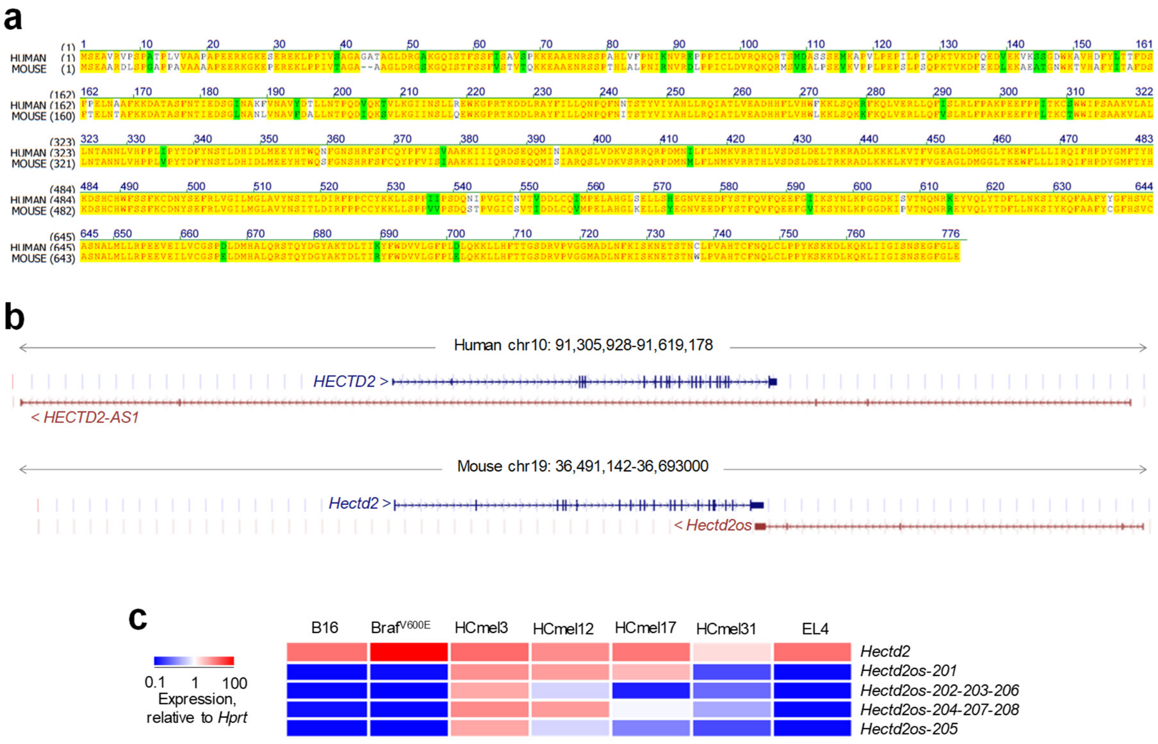

Figure S2. Comparison of human and murine HECTD2. a, Sequence alignment human and murine HECTD2 amino acid sequence. Yellow shading denote amino acid identity. Note that although the human reference HECTD2 has a Proline in position 19, the actual sequence of all alleles we have inspected has an Alanine, similar to the mouse sequence. **b**, Transcripts produced from the human *HECTD2* (top) and murine *Hectd2* (bottom) loci, showing both sense and antisense transcription. **c**, Expression levels of sense and antisense transcripts produced from murine *Hectd2* loci in the indicated murine melanoma cell line or EL4 lymphoma cells. Annotation of antisense transcripts is according to *Ensembl*.

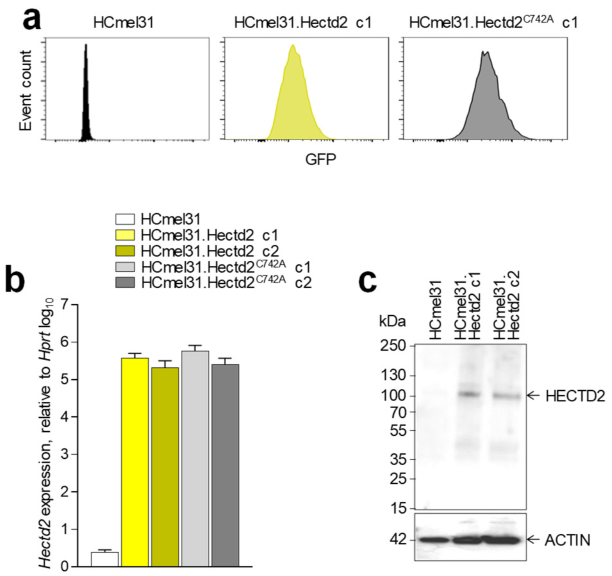

Figure S3. HECTD2 and HECTD2^{C742A} overexpression in HCmel31 murine melanoma cells. a, Expression of GFP from retroviral vector producing a bicistronic message also encoding murine HECTD2 or HECTD2^{C742A} in parental HCmel31 cells or those transduced with the respective vector. **d**, Expression of *Hectd2*, assessed by RT-qPCR in parental HCmel31 cells or those transduced the express HECTD2 or HECTD2^{C742A}. Two clones with slightly different expression levels were established for each variant. Bars indicate the mean (±SEM) *Hectd2* expression of technical replicates, relative to *Hprt* expression in the same samples. **c**, Western blotting of HECTD2 in parental HCmel31 cells and two clones of HCmel31 cells overexpressing HECTD2 (HCmel31.Hectd c1 and HCmel31.Hectd c2). Blotting for ACTIN is also included as a loading control. The blots are from a single experiment.

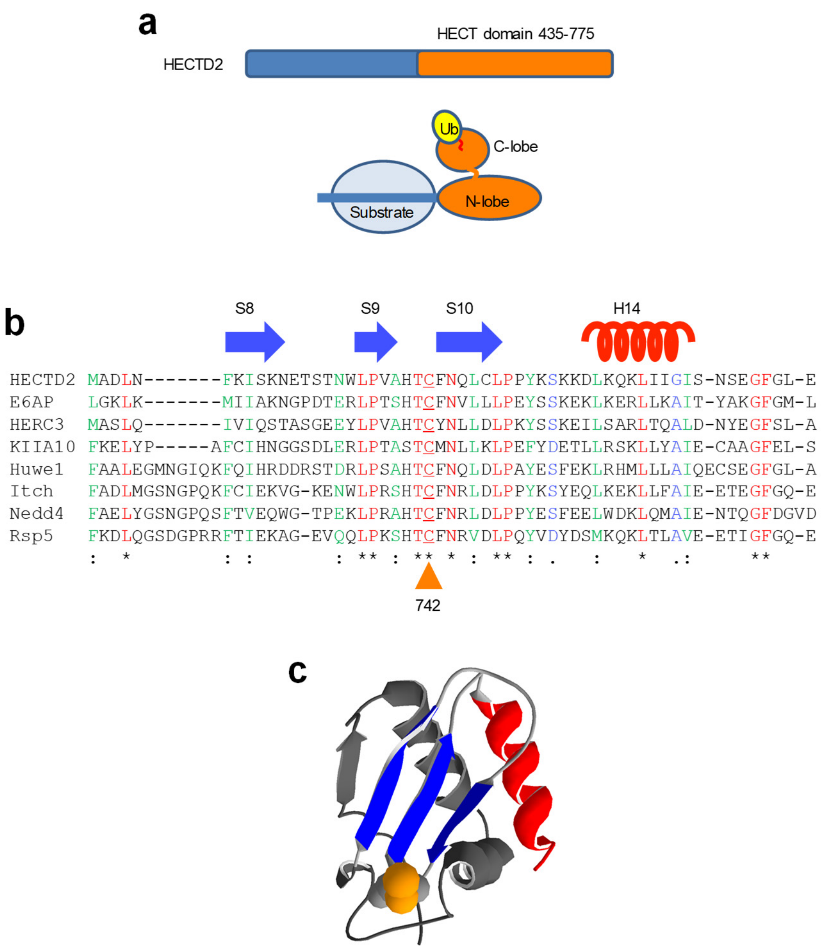

Figure S4. Prediction of a catalytically-inactive HECTD2 variant. **a**, Schematic representation of murine HECTD2 depicting the HECT domain. **b**, Sequence alignment of the last 60 amino acid residues of the HECT domain C-lobes of several E3 ubiquitin ligases. Identical residues are shown in red, with highly conserved residues in green. The conserved Cysteine at position 742 of murine HECTD2 is indicated by the orange arrow. **c**, Position of cysteine 742 (orange) in the predicted structure of the murine HECTD2 HECT domain.

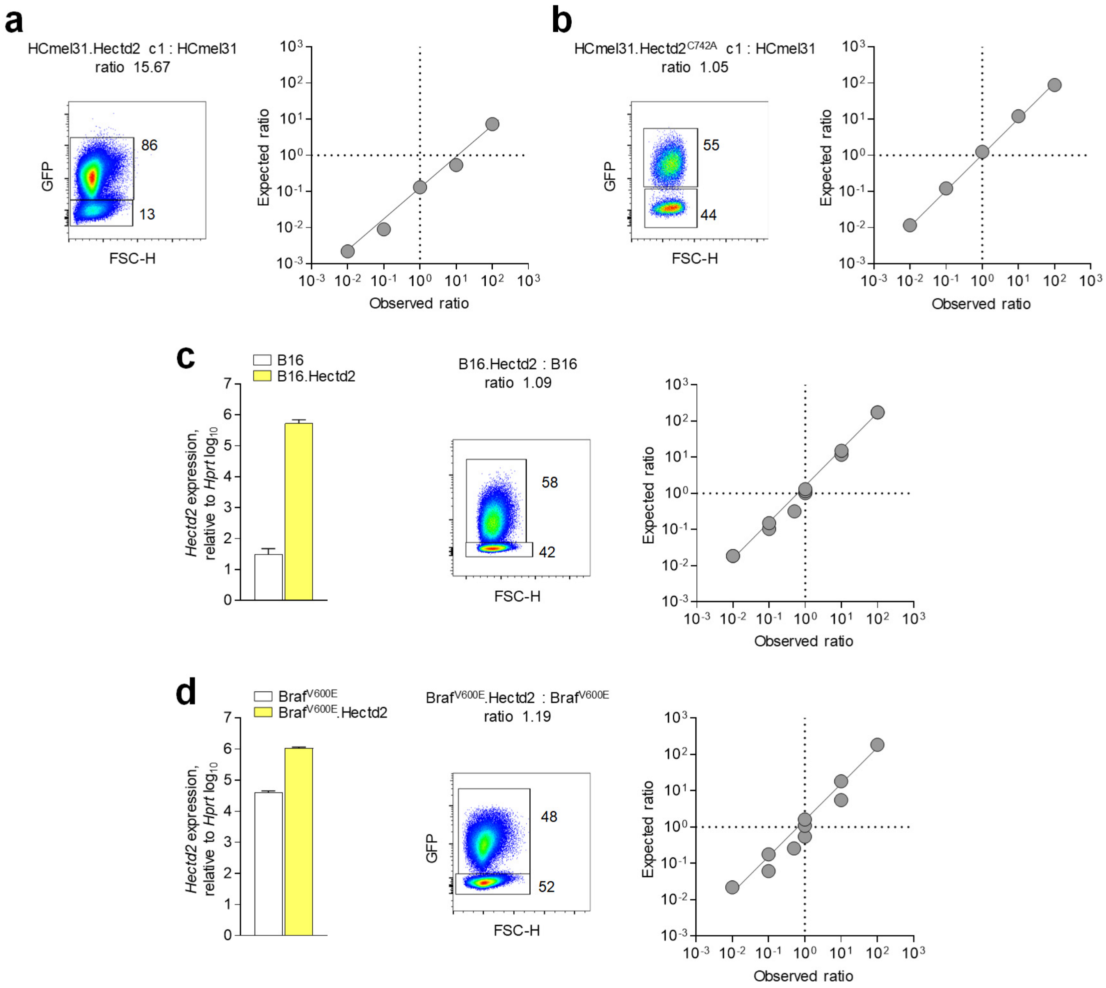

Figure S5. Effect of HECTD2 expression on murine melanoma cell growth in competition assays. In each case, parental cells and cells expressing either HECTD2 or HECTD2^{C742A} were mixed together at titrated ratios and were distinguished by GFP expression only in those expressing one of the HECTD2 variants. Deviation from the input ratio at the end of the competitive growth assay was then used to determine the effect of HECTD2 overexpression. **a**, Flow cytometric example (left) of the ratio of HCmel31 cells (GFP$^-$) and HCmel31.Hectd2 c1 cells (GFP$^+$) at the end of the competitive growth and expected ratio plotted against the observed ratio for these two populations (right). **b**, Flow cytometric example (left) of the ratio of HCmel31 cells (GFP$^-$) and HCmel31.Hectd2^{C742A} c1 cells (GFP$^+$) at the end of the competitive growth and expected ratio plotted against the observed ratio for these two populations (right). **c**, Mean (±SEM) expression of *Hectd2*, assessed by RT-qPCR in B16 and B16.Hectd2 cells (left), flow cytometric example (middle) of the ratio of B16 cells (GFP$^-$) and B16.Hectd2 cells (GFP$^+$) at the end of the competitive growth and expected ratio plotted against the observed ratio for these two populations (right). **d**, Mean (±SEM) expression of *Hectd2*, assessed by RT-qPCR in Braf^{V600E} and Braf^{V600E}.Hectd2 cells (left), flow cytometric example (middle) of the ratio of Braf^{V600E} cells (GFP$^-$) and Braf^{V600E}.Hectd2 cells (GFP$^+$) at the end of the competitive growth and expected ratio plotted against the observed ratio for these two populations (right).

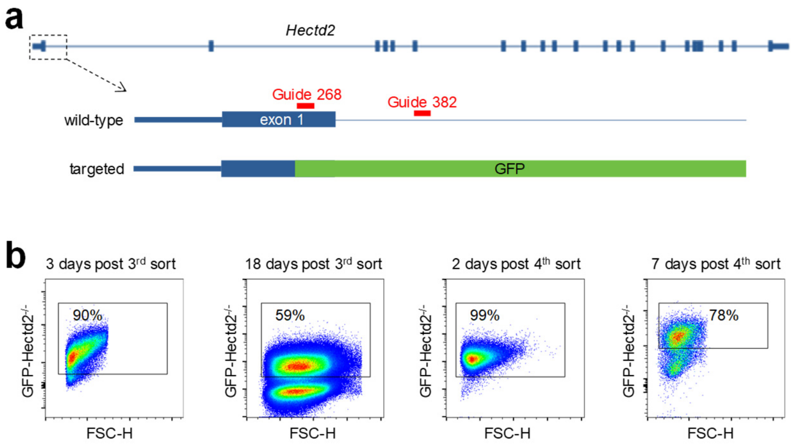

Figure S6. Effect of HECTD2 loss on murine melanoma growth *in vitro*. a, Murine *Hectd2* locus depicting the strategy for introducing a promoterless GFP encoding gene in the first exon of *Hectd2*. Red lines indicate the position of the Cas9 guide oligonucleotides used. **b**, GFP expression marking *Hectd2*-targeted cells in a population of Braf^{V600E} cells at the indicated times after successive FACS selection for GFP^{+} cells. Note the drop in the percentage of GFP^{+} cells after each sort.

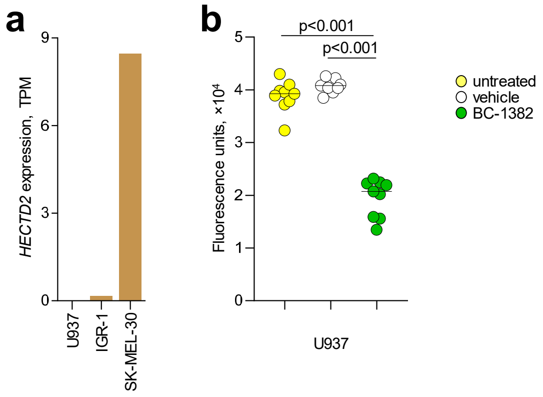

Figure S7. Effect of the BC-1382 inhibitor on the growth of the HECTD2-negative U937 cell line. **a**, Expression of *HECTD2* (TPM) in monocytic U937 cells compared with melanoma IGR-1 and SK-MEL-30 cells in data from CCLE. **b**, Growth of U937 cells over 5 days of culture, in the absence of treatment or in the presence of the 200 µM BC-1382 inhibitor or of the DMSO (vehicle) alone. Cell accumulation at the end of the culture period was assessed by AlamarBlue fluorescence and expressed as arbitrary fluorescence units.

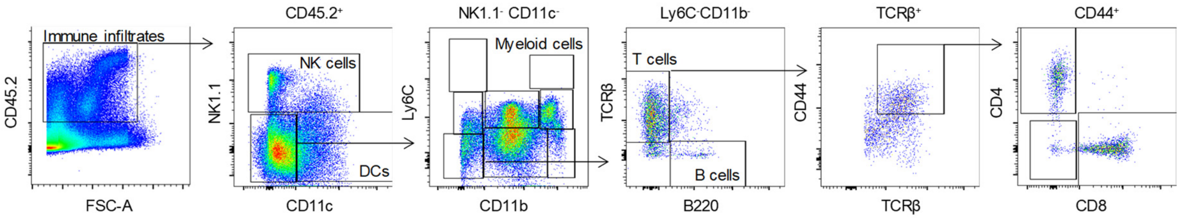

Figure S8. Flow cytometric detection of immune infiltrates in murine melanomas. Single cell suspensions prepared from murine melanomas were stained with the indicated antibodies and immune subpopulations were defined according to this gating scheme.

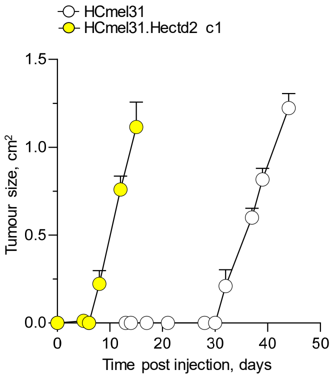

Figure S9. Effect of HECTD2 overexpression on murine melanoma growth in immunodeficient hosts. Mean size (±SEM) of tumours formed over time following injection of HCmel31 cells or HCmel31.Hectd2 c1 cells into severely immunodeficient *Rag1$^{-/-}$Il2rg$^{-/-}$Cd47$^{-/-}$* recipients (n=5 and n=8, respectively).

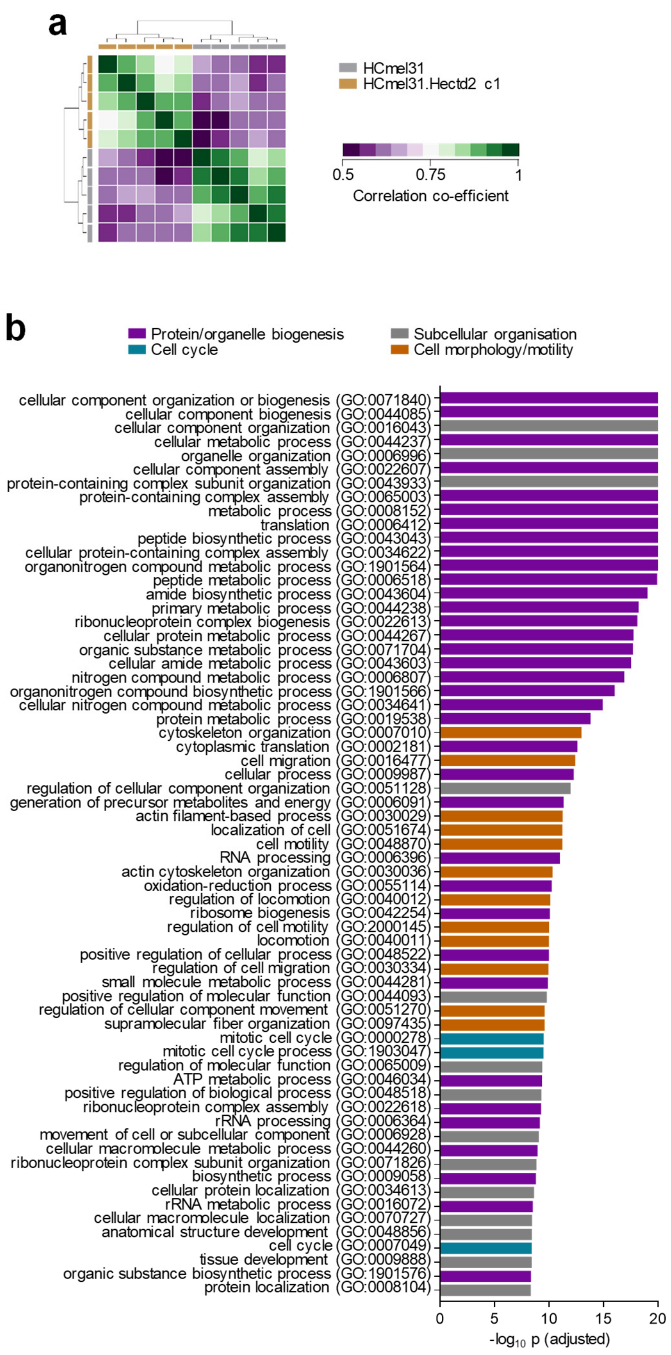

Figure S10. Effect of HECTD2 loss on the proteomic profile of murine melanoma cells. **a**, The proteomic profiles of HCmel31 and HCmel31.Hectd2 c1 cells were determined in quintuplicate samples. Hierarchical clustering of the samples based on the overall proteomic profiles distinguishes HCmel31 and HCmel31.Hectd2 c1 cells. **b**, Protein functional annotation analysis of the 990 proteins that were differentially abundant (≥2-fold, $p \leq 0.05$, $q \leq 0.05$) between HCmel31 and HCmel31.Hectd2 c1 cells.
