# Supplementary material for: E3 ubiquitin ligase HECTD2 mediates melanoma progression and immune evasion
Source: Oncogene. 2021 Jun 18;40(37):5567–78. doi: 10.1038/s41388-021-01885-4 (PMC8445817; doi:10.1038/s41388-021-01885-4)
Supplement: Supplementary file 4 — Table S1 [file 41388_2021_1885_MOESM4_ESM.pdf]

Table S1. Ratio of protein abundance in HCmel31.Hectd2 c1 over HCmel31 cells

Protein Groups	Aerage Log2 Ratio	P value	Q value	# of Ratios	Genes	# Unique Total Peptides	% Change	Ratio
Q2TA50	-6.20	3.74E-15	1.21E-14	30	Mlana	2	-98.64	0.01
D3YVY4;D3YWW2;D3YWW4;D3YY79;D3Z266;D3Z4Q6;F6WUX1;Q60928	-5.85	0.00045285	0.000298	10	Ggt1	1	-98.27	0.02
Q60696;Q9CZB2	-5.51	2.43E-32	3.50E-31	90	Pmel	7	-97.80	0.02
P07147	-5.11	3.65E-53	1.73E-51	180	Tyrp1	15	-97.10	0.03
P29812	-5.06	4.19E-62	2.77E-60	220	Dct	15	-97.00	0.03
Q9DCJ9	-5.05	2.42E-05	2.12E-05	10	Npl	1	-96.98	0.03
A2AQU8;Q9D975	-5.01	0.00112234	0.00067178	10	Srxn1	1	-96.90	0.03
Q05816	-4.91	6.88E-27	6.51E-26	90	Fabp5	6	-96.68	0.03
F6WP10;O35465	-4.91	0.00115182	0.00068694	10	Fkbp8	1	-96.67	0.03
Q9D2V7	-4.85	0.00013311	9.99E-05	20	Coro7	3	-96.53	0.03
Q9D1C1	-4.82	1.31E-23	9.01E-23	80	Ube2c	7	-96.45	0.04
D3Z2R9;P35505	-4.70	0.00300366	0.00159584	10	Fah	1	-96.14	0.04
P49813	-4.62	5.32E-12	1.30E-11	40	Tmod1	4	-95.94	0.04
Q9JK92	-4.54	0.00430165	0.00218381	10	Hspb8	2	-95.70	0.04
Q99P91	-4.52	4.40E-53	1.94E-51	210	Gpnmb	16	-95.63	0.04
A0A2I3BPT1;A0A2I3BQZ9;A0A2I3BR03;P12023;Q3TWF3	-4.48	8.18E-18	3.47E-17	40	App	4	-95.53	0.04
A0A5F8MPH5;A0A5F8MPX2;Q99N50	-4.47	1.08E-10	2.20E-10	30	Sytl2	5	-95.50	0.04
Q62425	-4.37	9.64E-10	1.72E-09	60	Ndufa4	6	-95.18	0.05
O88668	-4.31	1.57E-08	2.28E-08	20	Creg1	3	-94.96	0.05
Q9D7S7	-4.26	3.71E-09	6.00E-09	40	Rpl22l1	4	-94.79	0.05
B1B1A8;Q6PDN3	-4.17	0.00019282	0.00013951	20	Mylk	1	-94.45	0.06
P24668	-4.05	4.06E-11	8.84E-11	40	M6pr	3	-93.97	0.06
F8WIR1;P18242	-4.05	4.58E-17	1.79E-16	50	Ctsd	6	-93.95	0.06
Q91ZJ5	-4.02	3.49E-07	4.17E-07	30	Ugp2	6	-93.85	0.06
Q9R0P5	-4.01	5.81E-20	3.13E-19	100	Dstn	7	-93.80	0.06
Q9CWF2	-4.01	7.01E-11	1.50E-10	50	Tubb2b	5	-93.78	0.06
A0A087WQ70;Q07235	-4.00	0.00033435	0.00022796	10	Serpine2	2	-93.77	0.06
O09174	-3.88	0.00025697	0.00018155	10	Amacr	1	-93.22	0.07
Q9CQI6	-3.88	1.41E-05	1.31E-05	40	Cotl1	6	-93.19	0.07
P97370	-3.82	0.00284052	0.00153005	10	Atp1b3	2	-92.93	0.07
D3YX76;P15626	-3.78	0.0020668	0.00115462	10	Gstm2	1	-92.71	0.07
Q64442	-3.76	0.00093081	0.00057214	10	Sord	2	-92.62	0.07
Q8R2Y2	-3.71	0.00174796	0.00099156	10	Mcam	1	-92.35	0.08
P11157	-3.62	2.06E-10	4.06E-10	30	Rrm2	2	-91.87	0.08
A0A0A6YWU2;A0A0A6YY47;A0A0A6YY91;E9Q589;E9QB01;P13595	-3.60	0.00242274	0.00133543	10	Ncam1	1	-91.73	0.08
P53690	-3.59	0.0031301	0.00165241	10	Mmp14	2	-91.70	0.08
K3W4S6;Q9R062;V9GX26	-3.53	1.23E-18	5.70E-18	60	Gyg;Gyg1;Gyg	6	-91.35	0.09
O35215	-3.52	3.15E-07	3.80E-07	40	Ddt	4	-91.31	0.09
Q9QXY6	-3.51	0.00135234	0.00078848	20	Ehd3	2	-91.20	0.09
P62889	-3.50	3.89E-07	4.63E-07	50	Rpl30	5	-91.14	0.09
P50114	-3.44	6.85E-06	6.69E-06	20	S100b	2	-90.81	0.09
Q9CQX4	-3.44	9.10E-09	1.40E-08	30	Pclaf	3	-90.77	0.09
B7ZNH7;F7D5Y4;K3W4R4;Q80X19	-3.43	0.0394436	0.01501533	10	Col14a1	1	-90.72	0.09
A0A2I3BR94;Q9CQI3	-3.43	1.11E-12	2.84E-12	50	Gmfb	5	-90.69	0.09
B0V2N1	-3.42	0.00209424	0.00116896	10	Ptprs	1	-90.69	0.09
P56390	-3.42	3.05E-22	1.92E-21	90	Cks2	7	-90.68	0.09
P16460	-3.41	8.25E-11	1.72E-10	50	Ass1	6	-90.62	0.09
P60521	-3.41	2.10E-06	2.22E-06	10	Gabarapl2	1	-90.59	0.09
P70670;Q60817	-3.36	8.15E-27	7.60E-26	130	Naca	9	-90.24	0.10
A0A0G2JF03;A0A0G2JF31;A0A0G2JGG2;D6RCW9;P07607	-3.35	0.04356239	0.01641338	10	Tyms	1	-90.19	0.10
Q91XV3	-3.33	2.89E-16	1.02E-15	220	Basp1	16	-90.07	0.10
P16125	-3.31	2.44E-17	9.93E-17	80	Ldhb	8	-89.90	0.10
D3YX99;D3Z568;D3Z6W9;F6Y6V5;Q9CQH3	-3.30	1.99E-05	1.79E-05	20	Ndufb5	2	-89.87	0.10
G5E850;P56395	-3.29	1.06E-21	6.28E-21	120	Cyb5a	9	-89.76	0.10
Q3TQS0	-3.25	1.93E-05	1.74E-05	20	Calr4	1	-89.51	0.10
D3YVA2;Q91X97	-3.25	0.00279093	0.00151442	30	Ncald	3	-89.48	0.11
P97311;Q3ULG5	-3.23	0.00280461	0.00151563	10	Mcm6	2	-89.35	0.11
Q9D0K2	-3.23	2.21E-18	9.97E-18	140	Oxct1	15	-89.33	0.11
A0A0G2JG29;Q6ZWU9	-3.20	2.37E-27	2.41E-26	80	Rps27	6	-89.11	0.11
O54890	-3.20	0.00013458	0.0001009	10	Itgb3	1	-89.10	0.11
P62267	-3.17	9.83E-11	2.02E-10	90	Rps23	8	-88.85	0.11
Q9CR57	-3.16	1.05E-15	3.51E-15	120	Rpl14	9	-88.79	0.11
Q6PGH1	-3.16	1.19E-13	3.37E-13	40	Bud31	3	-88.79	0.11
A0A338P711;A0A338P7E3;C0HKG5;C0HKG6	-3.11	0.00096047	0.00058873	20	Rnaset2b;Rnaset2b;R	2	-88.40	0.12
P18155	-3.11	2.13E-14	6.53E-14	50	Mthfd2	5	-88.39	0.12
Q7TMM9	-3.09	0.02279707	0.00931584	10	Tubb2a	1	-88.30	0.12
P29416	-3.09	0.00710387	0.00339025	10	Hexa	1	-88.25	0.12
P62315	-3.08	0.00017105	0.00012531	20	Snrpd1	1	-88.18	0.12
A0A494B9B9;Q8C6I2	-3.06	1.82E-05	1.65E-05	20	Sdhaf2	1	-87.99	0.12
A0A0A6YWP9;A0A0A6YXY0;Q9CQ79	-3.03	0.00237265	0.00131329	10	Txndc9	1	-87.76	0.12
A0A1L1SR11;A0A1L1ST53;B2RUC1;O54818;Q3V2G6	-3.00	0.01012522	0.00455979	10	Tpd52l1	1	-87.49	0.13
Q6NV52;Q99LH8;Q9JJ11	-2.99	1.66E-05	1.52E-05	30	Tacc3	4	-87.45	0.13
A0A0G2JF16;Q8R344	-2.96	0.00021282	0.00015297	20	Ccdc12	2	-87.12	0.13
Q8VE99	-2.95	0.00022381	0.00016035	30	Ccdc115	4	-87.09	0.13
P97315	-2.94	9.17E-13	2.37E-12	50	Csrp1	6	-86.97	0.13
Q9D1K2	-2.94	6.17E-10	1.14E-09	70	Atp6v1f	7	-86.94	0.13
Q9ESP1	-2.93	0.00019895	0.00014363	10	Sdf2l1	1	-86.89	0.13
P50580	-2.93	1.64E-18	7.51E-18	160	Pa2g4	14	-86.89	0.13
D3YZ62;D3Z4J3;Q99104	-2.91	1.11E-12	2.84E-12	50	Myo5a	4	-86.65	0.13
A0A3B2W7I6;A0A3B2W883;O70551	-2.90	0.00214609	0.00119689	10	Srpk1	1	-86.64	0.13
Q91Z50	-2.89	7.39E-06	7.16E-06	20	Fen1	4	-86.54	0.13
Q62048	-2.86	1.95E-15	6.44E-15	80	Pea15	7	-86.22	0.14
P62309	-2.84	0.00092635	0.00056993	10	Snrpg	1	-86.08	0.14
D3YZX8;P49962	-2.82	0.00048016	0.00031379	10	Srp9	1	-85.84	0.14
P97797	-2.81	6.48E-08	8.62E-08	30	Sirpa	5	-85.74	0.14
A0A2R8VKL5;E9Q066;G3X9Q6;Q8BWW4	-2.81	1.36E-14	4.26E-14	90	Larp4	12	-85.72	0.14
P23927	-2.80	8.53E-26	7.24E-25	200	Cryab	14	-85.62	0.14
E9PX89;E9Q705;Q8CEI1	-2.77	1.64E-05	1.51E-05	20	Bola3	1	-85.36	0.15
A2AFG7;A2AFG8;P11627;Q6PGJ3	-2.75	4.44E-07	5.22E-07	20	L1cam	2	-85.13	0.15
A0A0N4SUH8;Q9QZ23	-2.74	1.57E-08	2.28E-08	30	Nfu1	3	-85.01	0.15
Q6ZQ73	-2.73	7.98E-11	1.67E-10	20	Cand2	3	-84.98	0.15
A0A087WQM0;A0A087WQS0;A0A6I8MWZ2	-2.73	1.93E-18	8.74E-18	70	Tns1	8	-84.94	0.15
A0A0R4J0R1;O70404	-2.72	6.40E-18	2.73E-17	60	Vamp8	5	-84.87	0.15
Q80VP0	-2.71	0.01555647	0.00662276	10	Tecpr1	1	-84.75	0.15
Q62351;Q8C872	-2.70	2.09E-11	4.82E-11	50	Tfrc	5	-84.65	0.15
P56382	-2.68	3.77E-05	3.21E-05	40	Atp5f1e	3	-84.42	0.16
A0A0R4J0G7;A0A2I3BPW9;A0A2I3BQS1	-2.68	3.01E-05	2.60E-05	40	Dlgap5	4	-84.41	0.16
Q01320	-2.68	8.12E-19	3.81E-18	130	Top2a	14	-84.39	0.16
O88811	-2.67	1.04E-09	1.84E-09	40	Stam2	4	-84.33	0.16
A0A0A6YW01;A0A0A6YXT6;A0A0A6YXW3;Q3U9G9	-2.67	0.00037036	0.00024816	30	Lbr	2	-84.30	0.16
Q91WE1	-2.67	2.11E-05	1.88E-05	20	Snx15	4	-84.26	0.16

Q8C605;Q9WUA3	-2.66	4.64E-14	1.38E-13	70	Pfkp	6	-84.20	0.16
A0A2R8VJV3;P61924;Q8R3M1	-2.65	8.28E-08	1.08E-07	30	Copz1	3	-84.12	0.16
P26369;Q80XR5	-2.64	1.12E-05	1.06E-05	60	U2af2	6	-83.98	0.16
Q04447	-2.64	4.93E-26	4.30E-25	90	Ckb	7	-83.98	0.16
Q8BG32	-2.64	3.21E-10	6.13E-10	50	Psmd11	7	-83.91	0.16
Q9D1C9	-2.61	0.00929296	0.00424272	10	Rrp7a	2	-83.60	0.16
Q9EST4	-2.60	0.01708996	0.00719692	10	Psmg2	1	-83.45	0.17
P14901	-2.59	9.49E-14	2.71E-13	40	Hmox1	3	-83.44	0.17
Q8K183	-2.58	0.00057939	0.00037311	10	Pdxk	1	-83.24	0.17
Q6NZD2;Q9WV80	-2.57	3.02E-11	6.77E-11	80	Snx1	7	-83.21	0.17
A0A1B0GRV0;Q9Z0S1	-2.57	7.94E-15	2.51E-14	40	Bpnt1	4	-83.16	0.17
Q60597;Z4YJV4	-2.57	8.91E-17	3.39E-16	100	Ogdh	10	-83.16	0.17
Q9WVE8	-2.57	0.00010979	8.39E-05	50	Pacsin2	5	-83.16	0.17
A0A5F8MPX7;E9Q7G6;Q8VC70	-2.57	0.00043429	0.00028693	10	Rbms2	1	-83.15	0.17
F8VQ95;Q6Y685	-2.57	2.21E-05	1.96E-05	40	Tacc1	7	-83.13	0.17
F7AYW2;F8WHT3;Q7TPM1	-2.56	1.10E-05	1.03E-05	50	Prrc2b	5	-83.09	0.17
P57746	-2.55	4.08E-08	5.58E-08	40	Atp6v1d	5	-82.91	0.17
Q9CZX7	-2.55	8.70E-05	6.79E-05	10	Pip4p2	1	-82.88	0.17
B1AZ42;P0C0A3	-2.54	0.01241442	0.00540681	10	Chmp6	1	-82.85	0.17
D3YYB0;D6RH49;Q6ZWY3	-2.54	1.68E-09	2.90E-09	20	Rps27l	1	-82.83	0.17
Q9JJ66;V9GXW0	-2.53	0.00068314	0.00043111	10	Cdc20	1	-82.72	0.17
Q9CQV1	-2.53	0.00160363	0.00091439	10	Pam16	1	-82.64	0.17
P62242	-2.52	1.14E-25	9.44E-25	210	Rps8	16	-82.60	0.17
P29758	-2.50	7.92E-17	3.05E-16	80	Oat	10	-82.36	0.18
Q922S8	-2.48	2.95E-08	4.12E-08	20	Kif2c	3	-82.12	0.18
Q8BK35	-2.47	3.37E-13	9.02E-13	20	Nop53	3	-81.91	0.18
Q8K0D5	-2.45	0.00766834	0.00360031	20	Gfm1	3	-81.73	0.18
A0A0A6YVU8;Q9JKV1	-2.44	2.31E-09	3.89E-09	50	Gm9774;Adrm1	4	-81.59	0.18
P62717	-2.43	6.13E-14	1.78E-13	160	Rpl18a	10	-81.49	0.19
A0A0A6YWP6;A0A0A6YX18;Q8BVE3	-2.43	6.03E-10	1.12E-09	50	Atp6v1h	5	-81.48	0.19
Q62313	-2.43	2.98E-11	6.72E-11	30	Tgoln1	2	-81.43	0.19
Q9D787	-2.42	2.33E-05	2.05E-05	10	Ppil2	1	-81.30	0.19
Q3TWW8	-2.42	2.13E-05	1.89E-05	80	Srsf6	8	-81.29	0.19
A2A4J1;A2A4J3;P61290	-2.42	0.02965118	0.01171885	10	Psme3	1	-81.27	0.19
F6YBV1;Q8K0V4	-2.42	8.99E-06	8.64E-06	10	Cnot3	1	-81.25	0.19
P45591	-2.40	2.74E-13	7.39E-13	120	Cfl2	11	-81.12	0.19
Q7TNS2	-2.40	0.01972523	0.00817153	10	Micos10	1	-81.06	0.19
P97377	-2.39	3.32E-28	3.73E-27	90	Cdk2	7	-80.94	0.19
P48437	-2.38	0.00018728	0.00013594	10	Prox1	1	-80.83	0.19
Q9ER00	-2.38	1.83E-10	3.65E-10	90	Stx12	8	-80.77	0.19
P51881	-2.37	3.22E-26	2.92E-25	150	Slc25a5	18	-80.69	0.19
Q8BP27	-2.37	5.35E-07	6.15E-07	50	Sfr1	3	-80.68	0.19
A7M7Q8;F7ARZ1;Q3TI14;Q8VE22	-2.36	1.83E-09	3.15E-09	20	Mrps23	2	-80.56	0.19
P17918	-2.36	9.84E-12	2.37E-11	50	Pcna	6	-80.55	0.19
Q9CQQ7	-2.36	1.52E-11	3.57E-11	40	Atp5pb	3	-80.55	0.19
P48771	-2.36	0.00145741	0.00084115	40	Cox7a2	2	-80.50	0.20
Q9QZD9	-2.34	0.00046406	0.00030386	20	Eif3i	3	-80.23	0.20
Q8CGC6	-2.34	9.23E-08	1.20E-07	40	Rbm28	6	-80.21	0.20
Q5SYH2	-2.33	0.00026182	0.000184	10	Tmem199	1	-80.15	0.20
Q9Z1Z2	-2.33	0.00044538	0.00029396	40	Strap	3	-80.13	0.20
Q9DC71	-2.32	0.02518864	0.01014287	30	Mrps15	4	-79.99	0.20
A0A0U1RQ20;Q9EPB4	-2.32	0.01334166	0.0057689	10	Pycard	1	-79.94	0.20
Q60865	-2.32	2.01E-19	1.01E-18	120	Caprin1	14	-79.90	0.20
Q8BWY3	-2.31	2.51E-10	4.89E-10	40	Etf1	4	-79.84	0.20
P45376	-2.27	9.42E-22	5.62E-21	70	Akr1b1	8	-79.32	0.21
A2ATU9;Q8BY71	-2.27	0.00289516	0.00154939	10	Hat1	1	-79.27	0.21
E9Q0U7;Q61699	-2.27	6.14E-45	1.82E-43	240	Hsph1	21	-79.27	0.21
Q9CQZ5	-2.27	7.90E-10	1.42E-09	30	Ndufa6	4	-79.26	0.21
Q9Z1D1	-2.26	5.17E-20	2.81E-19	130	Eif3g	13	-79.18	0.21
Q8BH40	-2.25	2.87E-16	1.02E-15	60	Stx7	6	-78.98	0.21
O35900	-2.25	0.01539401	0.00656626	10	Lsm2	2	-78.95	0.21
P68040	-2.25	4.67E-07	5.46E-07	50	Rack1	6	-78.94	0.21
P46656	-2.24	0.02873704	0.01140566	10	Fdx1	1	-78.89	0.21
Q8R0Y6	-2.24	0.00029202	0.00020264	20	Aldh1l1	3	-78.76	0.21
D3Z365	-2.22	3.41E-11	7.58E-11	20	Hk1	1	-78.59	0.21
B1AXW5;B1AXW6;P35700	-2.22	2.72E-59	1.50E-57	240	Prdx1	17	-78.50	0.21
D3Z069;Q8K1N2	-2.21	0.0013339	0.00077938	30	Phldb2	4	-78.44	0.22
Q5SQ20;Q9EQ61	-2.21	0.00013263	9.97E-05	10	Pes1	1	-78.44	0.22
A0A1Y7VMC8;Q8VC77;Q9CZU0	-2.21	2.15E-06	2.27E-06	10	Akr1c20	1	-78.40	0.22
Q9DBC7	-2.21	7.71E-05	6.08E-05	20	Prkar1a	3	-78.34	0.22
Q8K0B2	-2.20	0.04170778	0.01576845	10	Lmbrd1	1	-78.31	0.22
P62702	-2.20	1.24E-50	4.84E-49	350	Rps4x	26	-78.22	0.22
P47911	-2.20	4.16E-23	2.81E-22	310	Rpl6	23	-78.17	0.22
P09671	-2.19	3.25E-19	1.62E-18	60	Sod2	3	-78.15	0.22
Q6P5E4	-2.19	0.04067828	0.0154321	20	Uggt1	2	-78.12	0.22
P33174	-2.19	6.14E-05	4.97E-05	50	Kif4	6	-78.05	0.22
O35593	-2.17	0.0019041	0.00107095	10	Psmd14	1	-77.82	0.22
Q80Y14	-2.17	0.00014767	0.00010984	40	Glrx5	3	-77.79	0.22
P62754	-2.17	2.21E-30	2.87E-29	280	Rps6	18	-77.77	0.22
P62307	-2.17	1.56E-09	2.70E-09	20	Snrpf	2	-77.76	0.22
P63325	-2.16	4.01E-26	3.54E-25	300	Rps10	18	-77.64	0.22
P16332	-2.15	7.58E-08	9.98E-08	20	Mmut	3	-77.49	0.23
A2AE89;P10649	-2.15	9.63E-10	1.72E-09	110	Gstm1	10	-77.47	0.23
Q91WN1	-2.15	4.18E-20	2.29E-19	80	Dnajc9	7	-77.45	0.23
A0A0U1RP62;A0A0U1RPI8;Q9BCZ4	-2.14	0.03138836	0.01233913	10	Selenos	1	-77.39	0.23
P20060	-2.14	0.01626583	0.00689371	10	Hexb	1	-77.38	0.23
P50518	-2.14	5.36E-36	9.34E-35	180	Atp6v1e1	18	-77.36	0.23
A3KFU5;A3KFU8;Q6PHQ9;Q91YZ8	-2.14	4.23E-13	1.12E-12	130	Pabpc4	12	-77.30	0.23
A0A0D9SEG8;E9QJS7;E9QMJ5;Q9DC42;Q9Z0M6	-2.14	0.01098279	0.00489274	10	Adgre5	1	-77.28	0.23
P35980	-2.14	1.58E-24	1.20E-23	240	Rpl18	17	-77.27	0.23
F8VPM7;Q99MI1;V9GXF0;V9GXH3;V9GXP8	-2.13	2.58E-10	5.00E-10	70	Erc1	9	-77.19	0.23
Q99KR7	-2.13	0.01682974	0.00710089	10	Ppif	2	-77.15	0.23
P25206	-2.12	2.28E-08	3.23E-08	70	Mcm3	10	-77.03	0.23
Q922F4	-2.12	1.04E-05	9.85E-06	30	Tubb6	4	-76.98	0.23
Q91WC0	-2.11	0.00184894	0.00104259	20	Setd3	2	-76.90	0.23
Q80VJ3	-2.11	8.29E-14	2.38E-13	50	Dnph1	4	-76.80	0.23
Q9WVB0;T1ECW4	-2.10	9.54E-09	1.46E-08	30	Rbpms	5	-76.75	0.23
Q59J78	-2.10	1.46E-05	1.35E-05	50	Ndufaf2	3	-76.73	0.23
P54071	-2.10	2.23E-28	2.55E-27	170	Idh2	14	-76.73	0.23
A0A494BA49;A0A494BAX2;D3Z4B2;Q9CWZ7	-2.09	0.0119829	0.00526739	10	Napg	1	-76.55	0.23
P61025	-2.09	4.71E-19	2.29E-18	70	Cks1b	5	-76.49	0.24
P10404;P11370	-2.08	3.31E-09	5.40E-09	20	;Fv4	2	-76.35	0.24
G3UZY2;P97493	-2.08	1.18E-08	1.75E-08	20	Txn2	2	-76.33	0.24

Q9CQE5	-2.08	0.00011279	8.61E-05	10	Rgs10	1	-76.32	0.24
Q8VDF2	-2.08	1.16E-16	4.34E-16	50	Uhrf1	7	-76.31	0.24
A0A0R4J091;D6RDT4;Q9JKP7	-2.08	4.55E-05	3.80E-05	20	Pole3	2	-76.30	0.24
P30412	-2.07	6.91E-06	6.74E-06	30	Ppic	4	-76.20	0.24
Q3V1H1	-2.07	7.28E-07	8.29E-07	20	Ckap2	3	-76.17	0.24
Q9D8T7	-2.06	0.03142457	0.01234603	80	Slirp	7	-76.07	0.24
P61255	-2.06	1.13E-27	1.19E-26	290	Rpl26	18	-76.00	0.24
A0A1Y7VMP9;Q9WVL3	-2.05	0.00361684	0.00188087	10	Slc12a7	1	-75.85	0.24
A2AL12;Q8BG05	-2.04	6.56E-42	1.74E-40	530	Hnrnpa3	31	-75.67	0.24
Q99KI0	-2.04	1.68E-40	4.13E-39	370	Aco2	33	-75.63	0.24
P83882	-2.04	0.02924942	0.01158081	20	Rpl36a	2	-75.63	0.24
A0A571BG95	-2.03	0.00245849	0.00135288	10	Abr	2	-75.56	0.24
P15532	-2.02	4.98E-37	9.69E-36	190	Nme1	14	-75.41	0.25
A0A2I3BRP6;Q0KL02	-2.02	0.00041566	0.00027544	20	Trio	2	-75.35	0.25
A0A6I8MWW6;B1AU71;Q5SS00	-2.02	0.04382969	0.0165047	10	Zdbf2	1	-75.34	0.25
A0A338P6W3;G5E8T2;O88196	-2.02	0.00084556	0.00052659	10	Ttc3	1	-75.32	0.25
P48024	-2.01	6.62E-19	3.15E-18	60	Eif1	4	-75.23	0.25
P62911	-2.01	2.12E-09	3.60E-09	130	Rpl32	10	-75.22	0.25
Q8BFZ9	-2.00	3.12E-08	4.33E-08	40	Erlin2	3	-75.04	0.25
A0A0R4J069;Q9JLI6	-2.00	7.86E-09	1.22E-08	20	Scly	2	-75.01	0.25
A0A1Y7VKX7;A0A1Y7VLU4;F8WJA0;Q9ESV0	-1.99	6.93E-05	5.54E-05	10	Ddx24	1	-74.90	0.25
Q3UH60	-1.99	3.07E-08	4.28E-08	30	Dip2b	4	-74.87	0.25
Q9DBG9	-1.99	3.67E-08	5.04E-08	30	Tax1bp3	3	-74.87	0.25
H3BL05;H3BLD9;Q8C1M2	-1.99	8.86E-05	6.89E-05	10	Zfp428;Zfp428;Znf42	1	-74.75	0.25
P63028	-1.98	2.10E-25	1.72E-24	120	Tpt1	8	-74.67	0.25
Q9D6F9	-1.98	1.09E-05	1.03E-05	10	Tubb4a	1	-74.63	0.25
K3W4T3;Q9Z1G4	-1.97	0.04993153	0.01847662	10	Atp6v0a1	1	-74.53	0.25
E9QN31	-1.97	1.91E-14	5.92E-14	90	Nop2	12	-74.47	0.26
P11031	-1.97	5.39E-18	2.32E-17	80	Sub1	8	-74.45	0.26
D3Z494;G5E895;S4R2G9	-1.97	0.00888794	0.00407749	10	Akr1b10	1	-74.40	0.26
A0A0J9YUI3;A0A0J9YVJ2;Q9CQT5	-1.96	0.00018363	0.00013359	10	Pomp	1	-74.28	0.26
Q99N92	-1.96	4.32E-07	5.10E-07	40	Mrpl27	3	-74.25	0.26
G3UY65;P24860;Q68EM3	-1.96	5.05E-10	9.47E-10	20	Ccnb1	2	-74.25	0.26
P49717	-1.96	3.43E-08	4.73E-08	40	Mcm4	6	-74.22	0.26
Q9JIH2	-1.95	3.90E-09	6.28E-09	60	Nup50	6	-74.19	0.26
Q8CHT0	-1.95	0.00065973	0.00041834	20	Aldh4a1	2	-74.17	0.26
Q921K2	-1.95	2.09E-08	2.98E-08	80	Parp1	8	-74.10	0.26
A0A338P736;A0A338P781;P63085	-1.94	6.48E-05	5.23E-05	10	Mapk1	1	-74.03	0.26
P19536;Q9D881	-1.94	2.36E-06	2.47E-06	60	Cox5b;Cox5b-ps	4	-74.02	0.26
Q9DBP5	-1.94	9.17E-24	6.46E-23	140	Cmpk1	13	-74.01	0.26
Q8BT07	-1.94	0.00176765	0.00100187	20	Cep55	2	-73.89	0.26
Q9CZD3	-1.94	2.12E-14	6.52E-14	180	Gars1	15	-73.86	0.26
P60843	-1.94	2.59E-14	7.82E-14	110	Eif4a1	8	-73.85	0.26
A0A0R4J0U2;Q0VGB7	-1.93	1.86E-07	2.31E-07	100	Ppp4r2	8	-73.74	0.26
P21126	-1.91	8.89E-08	1.16E-07	50	Ubl4a	5	-73.47	0.27
Q8K298	-1.91	1.70E-16	6.16E-16	130	Anln	13	-73.43	0.27
D3YYD9;D3YYE0;P47856	-1.90	6.85E-05	5.49E-05	10	Gfpt1	1	-73.27	0.27
P49718	-1.90	2.33E-05	2.05E-05	20	Mcm5	2	-73.20	0.27
Q8JZK9	-1.89	0.00042489	0.000281	10	Hmgcs1	1	-73.08	0.27
Q99KP6	-1.89	0.01114799	0.00495966	40	Prpf19	6	-73.04	0.27
P48962	-1.89	4.97E-07	5.77E-07	80	Slc25a4	8	-73.00	0.27
P11499	-1.89	3.28E-47	1.03E-45	520	Hsp90ab1	41	-72.98	0.27
B8JJF6;Q9Z2D1	-1.89	4.15E-05	3.51E-05	10	Mtmr2	1	-72.93	0.27
P97496;Q3UNN4	-1.88	1.15E-12	2.93E-12	150	Smarcc1	15	-72.81	0.27
Q9D5V6	-1.88	4.43E-18	1.93E-17	60	Syap1	7	-72.77	0.27
P10126	-1.87	1.35E-17	5.61E-17	380	Eef1a1	27	-72.73	0.27
A0A1B0GSU2;D3YVK4;D3YVK5;Q9WV96	-1.87	2.14E-05	1.90E-05	20	Timm10b;Gm45799;	2	-72.64	0.27
P14131	-1.87	1.62E-17	6.72E-17	170	Rps16	13	-72.60	0.27
Q62419	-1.87	5.97E-13	1.57E-12	90	Sh3gl1	9	-72.59	0.27
E9PVC5;E9PVC6;E9Q9E1;Q6NZJ6	-1.87	7.90E-39	1.69E-37	200	Eif4g1	17	-72.57	0.27
P97314	-1.87	0.00040853	0.00027099	60	Csrp2	5	-72.55	0.27
P62855	-1.86	1.22E-08	1.81E-08	70	Rps26	4	-72.43	0.28
A2AU62;Q64012	-1.85	1.85E-10	3.67E-10	100	Raly	11	-72.33	0.28
Q9D0N7	-1.85	0.00157961	0.0009038	10	Chaf1b	2	-72.29	0.28
Q9D945	-1.84	7.39E-10	1.34E-09	30	Llph	2	-72.08	0.28
P26040	-1.84	3.28E-18	1.46E-17	90	Ezr	8	-72.06	0.28
Q80YR5	-1.83	0.00098015	0.00059913	30	Safb2	3	-71.90	0.28
Q99JT9	-1.83	4.79E-05	3.97E-05	10	Adi1	1	-71.81	0.28
Q8K363	-1.82	6.84E-08	9.05E-08	20	Ddx18	2	-71.76	0.28
P13864	-1.82	2.22E-29	2.72E-28	110	Dnmt1	10	-71.70	0.28
P18760	-1.82	3.60E-28	3.97E-27	260	Cfl1	18	-71.65	0.28
P52293	-1.81	1.25E-19	6.31E-19	60	Kpna2	6	-71.53	0.28
P62869	-1.81	5.30E-14	1.57E-13	100	Elob	10	-71.42	0.29
E9Q6F0;E9Q8C5;G3X9I1;G5E911;Q91VU0	-1.80	0.00127266	0.00074823	10	Fam3c	1	-71.22	0.29
E9PX77;F8WGT1;Q68FL4	-1.79	2.63E-11	5.97E-11	30	Ahcyl2	3	-71.13	0.29
Q63850	-1.79	6.26E-05	5.05E-05	20	Nup62	3	-71.05	0.29
Q62426	-1.79	3.57E-09	5.81E-09	120	Cstb	7	-71.01	0.29
B1AUY7;B1AUY8;B1AUY9;B1AUZ1;Q3V4D5;Q9QY36	-1.78	4.02E-05	3.42E-05	40	Naa10	5	-70.95	0.29
P49312;Q5EBP8	-1.78	1.44E-32	2.12E-31	430	Hnrnpa1	28	-70.89	0.29
P25444	-1.78	2.61E-10	5.05E-10	130	Rps2	14	-70.83	0.29
O55029	-1.78	0.00593527	0.00289547	10	Copb2	1	-70.81	0.29
Q8BVQ5	-1.78	0.00022484	0.00016091	20	Ppme1	2	-70.81	0.29
Q810V0	-1.77	1.79E-05	1.62E-05	20	Mphosph10	5	-70.70	0.29
Q9WV02	-1.77	0.00039947	0.00026604	30	Rbmx	2	-70.60	0.29
P11440	-1.76	5.38E-09	8.56E-09	50	Cdk1	6	-70.48	0.30
Q9CWZ3	-1.76	0.01447335	0.00620153	50	Rbm8a	5	-70.42	0.30
Q8BK64	-1.76	2.75E-05	2.39E-05	20	Ahsa1	3	-70.38	0.30
A0A0N4SUH6	-1.75	0.00031116	0.00021412	10	Rbm8a2	1	-70.36	0.30
Q91YR1	-1.75	0.00024747	0.00017559	20	Twf1	3	-70.35	0.30
P57759	-1.75	3.08E-12	7.61E-12	50	Erp29	6	-70.34	0.30
Q9WV98	-1.75	0.00120138	0.0007101	20	Timm9	1	-70.25	0.30
A0A3Q4EBV4;Q3TJZ6	-1.75	0.00061281	0.00039234	10	Fam98a	1	-70.19	0.30
A0A1W2P7X0;E9QMV2;Q4KML4	-1.75	9.32E-06	8.93E-06	30	Abracl	2	-70.18	0.30
A5A4Y9;G3UZ30	-1.74	1.00E-05	9.56E-06	50	Ppp1r11	3	-70.15	0.30
P14211	-1.74	1.91E-49	6.43E-48	390	Calr	26	-70.07	0.30
Q8VDW0	-1.74	0.00291675	0.00155843	20	Ddx39a	2	-70.04	0.30
P80316	-1.74	1.95E-11	4.52E-11	140	Cct5	15	-70.01	0.30
Q63829	-1.74	0.00280358	0.00151563	10	Commd3	2	-70.00	0.30
Q8C156	-1.74	2.31E-07	2.84E-07	30	Ncaph	4	-69.97	0.30
D3Z7F7;Q8BFS6	-1.73	0.00030654	0.00021138	10	Cpped1	1	-69.96	0.30
F2Z471	-1.73	2.13E-21	1.23E-20	100	Vdac1	10	-69.90	0.30
P16045	-1.73	1.19E-11	2.84E-11	150	Lgals1	10	-69.90	0.30
Q99KX1	-1.73	0.00013538	0.00010138	10	Mlf2	1	-69.89	0.30

Q3UKC1	-1.72	0.02480156	0.01002967	10	Tax1bp1	2	-69.72	0.30
Q9D902	-1.72	1.29E-06	1.41E-06	20	Gtf2e2	2	-69.66	0.30
Q6PAM1	-1.72	7.08E-10	1.29E-09	30	Txlna	4	-69.63	0.30
P61027	-1.72	0.0144664	0.00620153	40	Rab10	3	-69.61	0.30
Q9CY62	-1.71	1.22E-08	1.80E-08	40	Rnf181	4	-69.51	0.30
P62849	-1.71	2.34E-11	5.37E-11	100	Rps24	10	-69.50	0.30
Q9Z0R9	-1.71	0.00100794	0.00061385	10	Fads2	1	-69.47	0.31
P20108	-1.71	0.01211405	0.00531445	10	Prdx3	1	-69.43	0.31
Q91WS0	-1.71	8.72E-16	2.93E-15	50	Cisd1	3	-69.34	0.31
P28271	-1.70	0.00612599	0.00297535	10	Aco1	2	-69.31	0.31
Q9CX86	-1.70	3.94E-10	7.45E-10	120	Hnrnpa0	10	-69.29	0.31
E9PZF0	-1.70	2.40E-18	1.07E-17	110	Gm20390	8	-69.17	0.31
P46061	-1.69	4.15E-05	3.51E-05	50	Rangap1	5	-69.07	0.31
P52760	-1.69	2.57E-06	2.68E-06	20	Rida	2	-69.02	0.31
Q8BGQ7	-1.69	2.09E-11	4.82E-11	150	Aars1	15	-69.01	0.31
Q9CPW4	-1.69	1.71E-07	2.15E-07	60	Arpc5	7	-69.01	0.31
Q920A5	-1.68	9.68E-11	2.00E-10	50	Scpep1	5	-68.86	0.31
E9Q5B5;O08528	-1.68	0.01575955	0.00668821	10	Hk2	1	-68.71	0.31
P62245	-1.68	3.81E-19	1.88E-18	120	Rps15a	12	-68.70	0.31
Q9D1E6	-1.67	0.04507105	0.01690483	10	Tbcb	3	-68.63	0.31
P70295;Q3U3K9	-1.66	0.00012949	9.75E-05	20	Aup1	3	-68.44	0.32
E9PY85;G3UZF1;Q99L28	-1.66	0.00180487	0.00102034	10	Rsl24d1;Gm20509;Rsl	1	-68.41	0.32
P67984	-1.66	3.61E-26	3.23E-25	140	Rpl22	10	-68.36	0.32
D3YWF8;D3Z198;Q9CQE3	-1.65	7.74E-07	8.76E-07	10	Mrps17	1	-68.17	0.32
B1AR50;Q9DCD6	-1.64	0.00027673	0.00019363	20	Gabarap	1	-68.01	0.32
Q8VHM5	-1.64	0.00940599	0.00429136	50	Hnrnpr	5	-67.95	0.32
Q9Z2U0	-1.63	1.44E-05	1.33E-05	90	Psma7	9	-67.76	0.32
Q8C845;Q9D8Y0	-1.63	7.37E-11	1.57E-10	100	Efhd2	9	-67.74	0.32
Q62261	-1.63	3.07E-33	4.72E-32	400	Sptbn1	41	-67.71	0.32
H9KV04;Q8R3C0	-1.63	9.45E-06	9.04E-06	20	Mcmbp	2	-67.68	0.32
D3YZE8;D3Z5P7;Q14B71	-1.62	0.00065067	0.00041378	10	Cdca2	1	-67.53	0.32
A0A5F8MPZ2;E9Q7C9;Q5UE59;Q7TNF4;Q8CD76	-1.62	5.07E-08	6.86E-08	100	Klc1	11	-67.51	0.32
A0A1L1STF0;Q6PDI6	-1.62	0.00888674	0.00407749	10	Mindy2	1	-67.41	0.33
Q8K3J1	-1.62	2.56E-08	3.63E-08	30	Ndufs8	3	-67.37	0.33
M0QWS4;Q9CR09	-1.62	0.01206234	0.00529655	10	Ufc1	1	-67.37	0.33
Q9CWX9	-1.61	0.00232044	0.00128654	10	Ddx47	1	-67.33	0.33
F8WIT2;P14824	-1.61	0.00132759	0.00077638	80	Anxa6	7	-67.32	0.33
D3Z4S3	-1.61	0.0112814	0.00500891	10	Ptrhd1	1	-67.31	0.33
A0A0R4J0S1;Q91W92	-1.61	7.27E-06	7.06E-06	20	Cdc42ep1	2	-67.30	0.33
P62311	-1.61	0.00035127	0.00023802	40	Lsm3	4	-67.26	0.33
A2AFK7;Q91VC3	-1.61	0.00062309	0.00039738	30	Eif4a3	2	-67.13	0.33
Q8R4U7	-1.60	1.76E-07	2.20E-07	20	Luzp1	2	-66.92	0.33
A0A1L1SS44;Q60709	-1.59	0.00121776	0.0007185	10	Aplp2	1	-66.88	0.33
Q9JJ80;S4R1Y5	-1.59	0.00081488	0.0005094	30	Rpf2	3	-66.87	0.33
Q9ERB0	-1.59	1.08E-09	1.91E-09	30	Snap29	4	-66.86	0.33
B1AU75;B1AU76;Q99MD9	-1.59	0.01191673	0.00524875	40	Nasp	5	-66.82	0.33
Q99K28	-1.59	7.18E-08	9.49E-08	40	Arfgap2	5	-66.80	0.33
O88746;Q3UDC3	-1.59	2.11E-08	3.00E-08	50	Tom1	5	-66.77	0.33
P24369	-1.59	1.27E-28	1.48E-27	280	Ppib	19	-66.74	0.33
P62082	-1.58	4.67E-27	4.48E-26	340	Rps7	21	-66.66	0.33
Q99M54	-1.58	0.00025802	0.00018171	10	Cdca3	2	-66.64	0.33
Q9CQM9	-1.58	2.77E-12	6.93E-12	60	Glrx3	7	-66.58	0.33
Q8R0E2;Q9CQP0	-1.58	0.00017364	0.00012688	10	Mrpl33	1	-66.58	0.33
P62320	-1.58	1.80E-11	4.19E-11	50	Snrpd3	4	-66.58	0.33
P62858	-1.58	0.00045746	0.00030043	180	Rps28	9	-66.54	0.33
E9PUD2	-1.58	1.86E-05	1.68E-05	20	Dnm1l	3	-66.53	0.33
A0A0R4J079;Q8BMP6	-1.58	0.03580957	0.01383077	10	Acbd3	1	-66.51	0.33
E9PYF4;E9PYI7;F6TFN2;F6VG99	-1.57	1.19E-05	1.12E-05	50	Lmo7	5	-66.43	0.34
O35943	-1.57	0.00975818	0.00442157	10	Fxn	1	-66.34	0.34
Q00612	-1.57	9.19E-09	1.41E-08	90	G6pdx	8	-66.31	0.34
Q8CAQ8	-1.57	1.30E-29	1.62E-28	350	Immt	29	-66.29	0.34
P16546	-1.57	1.94E-49	6.43E-48	660	Sptan1	65	-66.29	0.34
Q8C263	-1.57	0.00723846	0.00343502	10	Ska3	1	-66.29	0.34
Q91WK2	-1.56	0.00784013	0.00366796	60	Eif3h	8	-66.10	0.34
Q5F2E8	-1.56	0.01206526	0.00529655	10	Taok1	1	-66.08	0.34
P97477	-1.55	0.00138468	0.00080479	10	Aurka	1	-65.86	0.34
Q80W00	-1.55	5.45E-09	8.62E-09	20	Ppp1r10	3	-65.79	0.34
P24547	-1.55	5.24E-08	7.08E-08	110	Impdh2	12	-65.76	0.34
H3BKN0;Q1HFZ0	-1.54	0.00035188	0.00023818	30	Nsun2	3	-65.63	0.34
Q61171	-1.54	1.30E-09	2.28E-09	90	Prdx2	8	-65.58	0.34
Q9D8U8	-1.54	0.01154565	0.00509888	30	Snx5	3	-65.57	0.34
Q9CZM2	-1.53	9.33E-17	3.53E-16	180	Rpl15	12	-65.49	0.35
E9PVA8	-1.53	0.03566732	0.01378387	10	Gcn1	2	-65.40	0.35
B1ARW4;Q99LY9	-1.53	0.00358332	0.00186491	10	Ndufs5	2	-65.35	0.35
Q9DBE9	-1.53	5.99E-08	8.02E-08	60	Ftsj3	6	-65.34	0.35
P58044	-1.53	0.00058679	0.00037678	20	Idi1	3	-65.34	0.35
E9PZ58;P97355	-1.53	2.12E-05	1.88E-05	10	Sms	2	-65.29	0.35
P07901	-1.52	6.31E-15	2.02E-14	250	Hsp90aa1	22	-65.15	0.35
Q8R050	-1.52	3.99E-06	4.04E-06	30	Gspt1	2	-65.03	0.35
A0A0A6YW53;A0A0A6YWM8;A0A0A6YWR8;B2RUE8;B7ZNR9;E9PVG7;F8VPL5;P9	-1.51	0.00035584	0.00024038	50	Map4k4	5	-64.95	0.35
Q62159	-1.51	0.00411281	0.0020976	30	Rhoc	2	-64.91	0.35
Q69ZQ2	-1.51	0.00050917	0.00033105	20	Isy1	2	-64.90	0.35
Q9CZT6	-1.51	0.01136418	0.0050288	10	Cmss1	1	-64.78	0.35
D3YUM1;Q91YT0	-1.50	3.22E-07	3.88E-07	40	Ndufv1	4	-64.73	0.35
H3BIY9;H3BKM0;Q9DBG3	-1.50	0.00668672	0.0032044	20	Ap2b1	3	-64.71	0.35
Q9DCX2	-1.49	2.18E-12	5.52E-12	170	Atp5pd	14	-64.52	0.35
Q9CPQ1	-1.49	4.77E-08	6.47E-08	90	Cox6c	4	-64.46	0.36
Q9CQL7	-1.49	8.29E-11	1.73E-10	40	Mrfap1	4	-64.45	0.36
P62313	-1.49	4.40E-07	5.19E-07	30	Lsm6	3	-64.42	0.36
P51150	-1.49	4.81E-11	1.04E-10	60	Rab7a	6	-64.40	0.36
O08807	-1.49	8.81E-05	6.87E-05	20	Prdx4	2	-64.38	0.36
Q99JY0	-1.49	1.02E-08	1.54E-08	140	Hadhb	14	-64.37	0.36
Q9D903	-1.48	1.53E-07	1.95E-07	60	Ebna1bp2	6	-64.25	0.36
Q9D6L8	-1.48	0.0002573	0.00018159	10	Ppil3	1	-64.17	0.36
Q9Z1Z0	-1.48	2.45E-09	4.10E-09	100	Uso1	10	-64.17	0.36
Q6PB66	-1.48	2.62E-11	5.97E-11	90	Lrpprc	9	-64.16	0.36
Q8VCG1;Q9CQ43	-1.47	5.01E-06	4.99E-06	50	Dut	5	-63.85	0.36
Q8VDJ3	-1.46	5.94E-06	5.83E-06	130	Hdlbp	17	-63.66	0.36
E9Q6U4;Q3UWL8	-1.46	8.29E-06	8.02E-06	60	Pfdn4	6	-63.63	0.36
O88384;Q91XH6	-1.46	1.48E-14	4.62E-14	20	Vti1b	3	-63.59	0.36
P09103	-1.45	2.61E-12	6.54E-12	360	P4hb	34	-63.43	0.37
Q91WE4	-1.45	4.79E-05	3.97E-05	20	NaN	1	-63.36	0.37
A0A0N4SUH4;Q8BKS9	-1.44	0.01134877	0.00502534	20	Pum3	2	-63.24	0.37

Q8CG48	-1.44	6.63E-05	5.34E-05	160	Smc2	19	-63.14	0.37
A0A087WRM8;E9QM90;G3X9J4;Q148V7	-1.44	0.01084008	0.00483567	30	Relch	3	-63.08	0.37
A0A087WNL9;Q0VG62	-1.44	0.00062281	0.00039738	10	Rbis	1	-63.06	0.37
P62077	-1.43	0.00410999	0.0020976	30	Timm8b	3	-62.87	0.37
A0A1L1SQG7;E9PZQ0;K3W4M2	-1.43	7.35E-06	7.13E-06	10	Ryr1	1	-62.77	0.37
O09005	-1.43	0.0023156	0.00128493	10	Degs1	2	-62.77	0.37
O35685	-1.42	6.07E-21	3.46E-20	220	Nudc	16	-62.66	0.37
Q7TNE3	-1.42	3.07E-06	3.18E-06	70	Spag7	7	-62.59	0.37
A0A0R4J1J1	-1.42	0.00016689	0.00012289	40	Pnkd	4	-62.52	0.37
Q9WUD1	-1.41	3.31E-08	4.58E-08	90	Stub1	7	-62.46	0.38
P61222	-1.41	0.01289468	0.00559023	20	Abce1	3	-62.44	0.38
A0A494B953;A0A494B9F0;A0A494B9L5;A0A494BA39;A0A494BAG2;A0A494BAX(	-1.41	4.07E-05	3.45E-05	30	Nedd4l	3	-62.40	0.38
D3Z0L4;Q9CRB9;Q9D9P1	-1.41	1.10E-13	3.12E-13	120	Chchd3	11	-62.33	0.38
P29341	-1.40	6.31E-45	1.82E-43	450	Pabpc1	36	-62.19	0.38
G3UXX3;Q64105;Q91XH5	-1.40	3.20E-10	6.13E-10	50	Spr	5	-62.11	0.38
Q9DCS9	-1.40	0.00831683	0.00385286	20	Ndufb10	5	-62.05	0.38
Q8VDN2	-1.39	9.31E-07	1.04E-06	100	Atp1a1	11	-61.96	0.38
Q9Z2I8	-1.39	1.35E-11	3.21E-11	30	Suclg2	3	-61.93	0.38
Q9CQ62	-1.39	5.56E-06	5.49E-06	20	Decr1	2	-61.91	0.38
P39689	-1.39	1.14E-07	1.46E-07	20	Cdkn1a	1	-61.79	0.38
A2AFI9;A2AFJ1;Q60973	-1.38	1.82E-07	2.27E-07	50	Rbbp7	7	-61.66	0.38
Q9CXW3	-1.38	5.23E-18	2.26E-17	180	Cacybp	15	-61.64	0.38
Q6P8N8;Q9JHS4	-1.38	0.0335684	0.01307962	10	Clpx	2	-61.60	0.38
P18654	-1.38	6.39E-15	2.03E-14	40	Rps6ka3	4	-61.58	0.38
Q8VBZ3	-1.38	0.00827599	0.00383663	10	Clptm1	1	-61.53	0.38
Q5XJE5	-1.38	0.00354113	0.0018473	10	Leo1	1	-61.52	0.38
Q6ZPJ3	-1.38	6.91E-05	5.52E-05	10	Ube2o	1	-61.46	0.39
Q9D8M4	-1.37	0.01154435	0.00509888	10	Rpl7l1	1	-61.38	0.39
Q9WUM5	-1.37	6.11E-08	8.17E-08	60	Suclg1	5	-61.26	0.39
O55091	-1.37	0.01170903	0.00516758	10	Impact	1	-61.20	0.39
A2AEB3;A2AEB4;A2AEB5;Q9R0Q4	-1.36	8.44E-09	1.31E-08	50	Morf4l2	4	-61.16	0.39
Q8R1B4	-1.36	2.37E-07	2.91E-07	150	Eif3c	15	-61.03	0.39
P14115	-1.36	1.13E-05	1.06E-05	110	Rpl27a	8	-61.00	0.39
Q9CWK3	-1.36	0.00261047	0.00142939	20	Cd2bp2	2	-60.96	0.39
Q60648	-1.36	1.38E-09	2.41E-09	40	Gm2a	5	-60.94	0.39
Q8R3R8	-1.36	0.02633704	0.01055395	10	Gabarapl1	2	-60.92	0.39
Q9JMH6	-1.35	2.66E-05	2.32E-05	30	Txnrd1	2	-60.84	0.39
Q9D9E9	-1.35	0.00017493	0.00012768	10	1700086D15Rik	1	-60.83	0.39
Q3TXN1;Q8VDQ1	-1.35	0.00035562	0.00024038	10	Ptgr2	1	-60.72	0.39
P62806	-1.35	3.07E-15	9.96E-15	560	H4c1	29	-60.71	0.39
Q924C1	-1.34	3.52E-05	3.02E-05	10	Xpo5	1	-60.53	0.39
Q9D7Z3	-1.34	0.00023735	0.00016895	20	Nol7	2	-60.42	0.40
A0A2R8VHP9;A0A2R8VI25;B2M1R7;Q61990	-1.34	1.83E-10	3.65E-10	100	Pcbp2	9	-60.42	0.40
M0QWP2;M0QWY0;O70378	-1.34	8.50E-05	6.66E-05	40	Emc8	4	-60.39	0.40
Q9D1Q1	-1.33	0.00465	0.00234091	20	Mphosph6	2	-60.33	0.40
Q921N6	-1.33	0.02064349	0.00850404	40	Ddx27	5	-60.29	0.40
A0A3B2WBL1;P53026;Q5XJF6	-1.33	9.67E-13	2.49E-12	250	Rpl10a	15	-60.27	0.40
Q8VCI5	-1.33	0.00026159	0.000184	30	Pex19	3	-60.25	0.40
E9PYI8;Q9JMA1	-1.33	2.03E-06	2.16E-06	30	Usp14	5	-60.11	0.40
Q9CQF8	-1.32	0.01966431	0.00815139	10	Mrpl57	1	-60.04	0.40
O35604	-1.32	0.00657093	0.0031636	20	Npc1	2	-60.00	0.40
Q9ERD7	-1.32	3.31E-07	3.97E-07	80	Tubb3	5	-59.94	0.40
A0A0A6YWB3;Q3TFK5	-1.32	0.00427324	0.00217106	10	Gpatch4	1	-59.93	0.40
Q8R0A0	-1.32	3.38E-05	2.90E-05	70	Gtf2f2	7	-59.86	0.40
P63017	-1.32	6.92E-90	9.16E-88	1060	Hspa8	63	-59.82	0.40
A0A1B0GSV1;Q9D8P4	-1.31	0.0046665	0.00234387	10	Mrpl17	1	-59.79	0.40
Q76KJ5	-1.31	1.00E-06	1.11E-06	20	Polr1g	2	-59.67	0.40
Q9D0M3	-1.31	4.20E-05	3.54E-05	50	Cyc1	5	-59.67	0.40
E9PX68;E9Q585	-1.31	0.04266657	0.01609417	20	Slc4a1ap	2	-59.67	0.40
Q9D2R8	-1.31	0.02001705	0.00828206	10	Mrps33	1	-59.61	0.40
A3KGG6;A3KGG8;F7BE71;H3BKK2;Q9D9Z1	-1.31	0.03463481	0.01345554	10	Knstrn	1	-59.55	0.40
A0A087WNV1;A0A087WR52;A0A087WSR7;Q8K2K6	-1.30	2.03E-06	2.16E-06	30	Agfg1	3	-59.49	0.41
P50516	-1.30	2.19E-09	3.71E-09	80	Atp6v1a	8	-59.48	0.41
A0A0J9YTY0;A0A0J9YUL3;Q8C1B7	-1.30	1.69E-10	3.42E-10	170	Septin11	13	-59.45	0.41
Q9D3B1	-1.29	4.38E-05	3.68E-05	10	Hacd2	1	-59.08	0.41
Q9ER81	-1.28	0.01224925	0.00535955	20	Tor1aip2	1	-58.92	0.41
Q9WVA4	-1.28	7.05E-39	1.55E-37	380	Tagln2	25	-58.87	0.41
Q99KV1	-1.28	5.07E-10	9.49E-10	40	Dnajb11	2	-58.86	0.41
P62814	-1.28	2.99E-11	6.74E-11	130	Atp6v1b2	12	-58.84	0.41
Q9D823	-1.28	0.01355007	0.00584755	20	Rpl37	1	-58.81	0.41
Q64337	-1.28	6.74E-20	3.54E-19	120	Sqstm1	9	-58.79	0.41
Q9DB05	-1.28	0.00090662	0.00056039	20	Napa	5	-58.78	0.41
P0DN34	-1.28	3.51E-07	4.19E-07	30	Ndufb1	2	-58.75	0.41
Q9QYH6	-1.28	1.54E-05	1.42E-05	30	Maged1	3	-58.70	0.41
G3UY42;Q8CCS6	-1.28	0.00588542	0.00287751	50	Pabpn1	4	-58.69	0.41
O09111	-1.27	0.00225639	0.00125313	10	Ndufb11	2	-58.63	0.41
G3UWQ7	-1.27	1.31E-05	1.22E-05	20	Prc1	2	-58.63	0.41
Q9D819	-1.27	1.47E-15	4.88E-15	120	Ppa1	10	-58.52	0.41
F6YVP7;P62270	-1.27	8.91E-34	1.48E-32	280	Rps18-ps6;Rps18	22	-58.52	0.41
F6ZFT1;F8WJ64;Q9CR21	-1.27	0.02657946	0.01062317	60	Ndufab1	4	-58.49	0.42
Q62465	-1.27	1.72E-26	1.58E-25	130	Vat1	12	-58.47	0.42
A0A5F8MPN8;P14152	-1.27	9.59E-07	1.07E-06	80	Mdh1	6	-58.46	0.42
Q9D0B6	-1.26	0.00189873	0.00106884	10	Pbdc1	1	-58.31	0.42
Q9WUA2	-1.26	0.00131324	0.00076935	30	Farsb	3	-58.31	0.42
A2AFQ0;Q7TMY8	-1.25	4.78E-07	5.57E-07	130	Huwe1	14	-58.01	0.42
D5MCW4;Q9CQ89	-1.25	0.00013786	0.00010313	20	Cuta	2	-57.92	0.42
P70372	-1.25	1.54E-07	1.95E-07	160	Elavl1	16	-57.86	0.42
P62908	-1.25	3.52E-11	7.76E-11	140	Rps3	15	-57.84	0.42
P97379	-1.24	4.71E-30	6.00E-29	220	G3bp2	18	-57.58	0.42
Q3V4B5	-1.24	3.31E-05	2.84E-05	20	Commd6	2	-57.57	0.42
Q640M1	-1.24	0.00016281	0.00012029	90	Utp14a	11	-57.55	0.42
Q9WTP7	-1.23	1.24E-07	1.58E-07	50	Ak3	5	-57.48	0.43
Q9DAM7	-1.23	0.0004033	0.00026779	30	Tmem263	3	-57.38	0.43
Q9D1A2	-1.23	1.32E-05	1.23E-05	60	Cndp2	5	-57.32	0.43
P63037	-1.23	1.02E-25	8.57E-25	140	Dnaja1	13	-57.32	0.43
Q9JIK9	-1.23	2.64E-05	2.31E-05	20	Mrps34	3	-57.29	0.43
Q3THS6	-1.23	2.42E-06	2.52E-06	60	Mat2a	6	-57.27	0.43
Q9D6J6	-1.23	1.07E-05	1.02E-05	40	Ndufv2	6	-57.25	0.43
Q8R2Q8	-1.23	0.00130216	0.00076353	20	Bst2	3	-57.23	0.43
Q99LE6	-1.22	0.0046082	0.00232163	30	Abcf2	3	-57.14	0.43
Q8C5G6;Q9QZ06	-1.22	1.59E-06	1.71E-06	20	Tollip	2	-57.11	0.43
Q9CR51	-1.22	3.23E-07	3.89E-07	70	Atp6v1g1	5	-57.03	0.43
Q9WUM4	-1.22	2.03E-05	1.81E-05	70	Coro1c	6	-56.96	0.43

Q9JJ94	-1.22	0.03622586	0.01395897	30	Ssna1	3	-56.95	0.43
D3YY13;D3YZT4;D3Z1Z3;D6REF7;Q8R0X7	-1.21	0.02488689	0.01003968	10	Sgpl1	1	-56.89	0.43
P80315	-1.21	2.19E-07	2.71E-07	140	Cct4	13	-56.88	0.43
Q62418	-1.21	4.52E-27	4.40E-26	180	Dbnl	15	-56.69	0.43
B1ASC2;Q99LH1	-1.21	0.00030555	0.00021092	10	Gnl2	1	-56.67	0.43
Q9Z0J0	-1.20	1.01E-09	1.81E-09	100	Npc2	6	-56.48	0.44
E9Q3V6;P42208	-1.20	0.00108965	0.00065637	190	Septin2	16	-56.39	0.44
B2FDG7;Q99N87	-1.19	0.00850412	0.00392863	10	Mrps5	1	-56.25	0.44
P97807	-1.19	1.57E-06	1.70E-06	80	Fh	8	-56.12	0.44
P14206	-1.18	4.01E-20	2.21E-19	160	Rpsa	14	-56.01	0.44
Q3V3R1	-1.18	1.55E-06	1.67E-06	90	Mthfd1l	13	-56.00	0.44
P30416	-1.18	4.53E-28	4.84E-27	210	Fkbp4	14	-55.96	0.44
Q9JJY4	-1.18	0.00063079	0.00040152	10	Ddx20	1	-55.91	0.44
Q8R3N1	-1.18	5.39E-06	5.33E-06	20	Nop14	4	-55.90	0.44
Q9DB77	-1.18	0.0075375	0.00355148	50	Uqcrc2	4	-55.87	0.44
Q9R1T2	-1.18	0.01989456	0.00823652	50	Sae1	5	-55.78	0.44
P09405	-1.17	5.27E-37	9.97E-36	690	Ncl	52	-55.66	0.44
Q91YQ5	-1.17	3.10E-11	6.90E-11	80	Rpn1	7	-55.59	0.44
Q6PGB6	-1.17	1.30E-08	1.91E-08	60	Naa50	7	-55.51	0.44
O35226	-1.17	0.00079415	0.00049738	70	Psmd4	8	-55.49	0.45
J3QN19;P20664	-1.17	0.00693142	0.00331546	10	Prim1	1	-55.42	0.45
P61358	-1.16	4.79E-32	6.75E-31	250	Rpl27	14	-55.30	0.45
Q9CQK7	-1.16	8.24E-05	6.48E-05	20	Rwdd1	2	-55.11	0.45
A0A0A6YX02;Q9CQ22	-1.15	0.00074639	0.00046924	40	Lamtor1	4	-55.05	0.45
Q91W45	-1.15	0.04696494	0.01750607	10	Paip2b	2	-55.02	0.45
P63101	-1.15	1.03E-10	2.12E-10	240	Ywhaz	17	-54.99	0.45
P54726;Q3TN85;Q8CAP3	-1.15	0.00357299	0.00186099	10	Rad23a	2	-54.98	0.45
Q9CPY1	-1.15	1.66E-05	1.52E-05	20	Mrpl51	3	-54.90	0.45
Q8VEJ9	-1.15	2.25E-06	2.36E-06	20	Vps4a	2	-54.86	0.45
Q9D554	-1.15	0.00563637	0.00277212	80	Sf3a3	9	-54.81	0.45
A2AP32;Q3UIU2	-1.14	0.01095751	0.00488476	20	Ndufb6	3	-54.76	0.45
Q6PGC1	-1.14	0.00450436	0.00227278	10	Dhx29	2	-54.72	0.45
P47955	-1.14	1.44E-06	1.57E-06	20	Rplp1	1	-54.68	0.45
Q91YT7	-1.14	0.01672637	0.00706628	40	Ythdf2	5	-54.64	0.45
Q8R2K3	-1.14	4.07E-06	4.11E-06	40	Ssbp1	5	-54.63	0.45
Q9WTP6	-1.14	1.58E-20	8.85E-20	120	Ak2	8	-54.61	0.45
Q91VE6	-1.14	0.00078643	0.00049301	20	Nifk	4	-54.58	0.45
A0A338P734;A0A338P7J7;Q3UFY4;Q9DA80	-1.14	0.00590759	0.00288622	10	Rsph3b;Rsph3a;Rsph3	1	-54.52	0.45
Q8VCR4;Q9DCG9	-1.13	2.82E-09	4.66E-09	60	Trmt112	5	-54.45	0.46
A0A498WGD8;Q8CDN6	-1.13	1.87E-09	3.19E-09	100	Txnl1	9	-54.39	0.46
O35737;Q8C2Q7	-1.13	0.02190198	0.00898334	60	Hnrnph1	6	-54.33	0.46
Q3UE92	-1.13	0.0037524	0.00194221	40	Xpnpep1	4	-54.28	0.46
A0A1Y7VKZ4;Q91VK1	-1.13	0.01785611	0.00749097	30	Bzw2	2	-54.27	0.46
Q8K2Y7	-1.13	0.00641355	0.00309684	50	Mrpl47	6	-54.26	0.46
Q9D892	-1.13	5.05E-05	4.17E-05	70	Itpa	6	-54.19	0.46
Q9CQE8	-1.13	9.12E-05	7.08E-05	70	RTRAF	6	-54.17	0.46
Q8K0C4	-1.12	0.00344823	0.00180739	20	Cyp51a1	3	-54.07	0.46
P59325	-1.12	2.79E-08	3.91E-08	110	Eif5	11	-53.98	0.46
P80314	-1.12	5.40E-10	1.01E-09	210	Cct2	19	-53.92	0.46
A0A0N4SW28;Q9DAS9	-1.12	1.39E-05	1.29E-05	30	Gng12	5	-53.89	0.46
D3YX62;D3YXN4;O70252	-1.11	0.00018179	0.00013239	10	Hmox2	1	-53.82	0.46
Q9JII6	-1.11	3.97E-17	1.57E-16	120	Akr1a1	11	-53.69	0.46
Q5RL20	-1.11	0.00099488	0.00060645	60	Mrpl43	6	-53.68	0.46
Q91VD9	-1.11	0.03712574	0.01425594	40	Ndufs1	5	-53.68	0.46
E9Q6W2;Q9D2R6	-1.11	1.70E-05	1.55E-05	30	Coa3	3	-53.62	0.46
Q9Z0G0	-1.11	0.00528957	0.00261907	20	Gipc1	2	-53.55	0.46
A0A087WNT1;A0A087WQE6;P83940	-1.10	0.01119819	0.00497864	20	Eloc	3	-53.46	0.47
Q8R332	-1.10	0.01264978	0.00549125	30	Nup58	3	-53.44	0.47
Q9CQA3	-1.10	0.00013104	9.86E-05	90	Sdhb	8	-53.32	0.47
P43024;Q9DCW5	-1.09	7.17E-05	5.71E-05	70	Cox6a1	4	-53.18	0.47
Q9JIK5	-1.09	2.16E-17	8.84E-17	190	Ddx21	21	-53.16	0.47
E0CYB9;E0CYI3;Q6PCP5	-1.09	0.0090176	0.00413076	30	Mff	3	-53.16	0.47
Q8BK30	-1.09	0.01951174	0.00809829	10	Ndufv3	1	-53.12	0.47
P14148	-1.09	1.56E-13	4.35E-13	380	Rpl7	23	-53.10	0.47
Q5U5I3;Q99N85	-1.09	0.02622328	0.01051473	10	Mrps18a	1	-53.06	0.47
E9PVZ8;E9QAH1	-1.09	0.01079892	0.00482055	50	Golgb1	7	-53.06	0.47
Q9CX34	-1.09	1.15E-16	4.33E-16	90	Sugt1	10	-53.00	0.47
O55142	-1.09	1.07E-11	2.56E-11	140	Rpl35a	12	-52.94	0.47
Q7TPE5	-1.08	1.22E-05	1.14E-05	20	Slc7a6os	2	-52.84	0.47
Q923D4	-1.08	1.35E-07	1.71E-07	60	Sf3b5	3	-52.79	0.47
Q50HX3;Q91V41	-1.08	0.01256224	0.005464	20	Rab14	2	-52.79	0.47
P58468	-1.08	2.87E-06	2.98E-06	20	Fam207a	3	-52.79	0.47
P62281	-1.08	0.00065512	0.00041621	300	Rps11	20	-52.76	0.47
P26516	-1.08	7.06E-06	6.88E-06	50	Psmd7	3	-52.74	0.47
A0A0U1RNJ1;P19096	-1.08	2.07E-07	2.57E-07	170	Fasn	18	-52.74	0.47
A0A0R4J0H7;Q8K2Z4	-1.08	0.00578917	0.00283674	10	Ncapd2	1	-52.71	0.47
F7BX26;Q60676	-1.08	5.35E-07	6.15E-07	20	Ppp5c	2	-52.70	0.47
A2ALA4;Q62276	-1.08	0.03298482	0.01289779	10	Med22	1	-52.70	0.47
P63166	-1.08	2.82E-14	8.47E-14	50	Sumo1	4	-52.67	0.47
O09172	-1.07	1.15E-06	1.26E-06	40	Gclm	4	-52.50	0.48
G3UVV4;P17710	-1.07	0.01132338	0.00502082	70	Hk1	9	-52.43	0.48
Q9WVM1	-1.07	0.00566767	0.00278361	10	Racgap1	2	-52.41	0.48
Q9R1J0	-1.07	0.00025776	0.00018171	30	Nsdhl	2	-52.35	0.48
G5E902;Q8VEM8	-1.07	0.00025376	0.00017967	70	Slc25a3	5	-52.29	0.48
D3YVZ9;P58059	-1.07	0.0001635	0.00012053	20	Mrps21	2	-52.22	0.48
Q78PY7	-1.06	2.52E-10	4.89E-10	200	Snd1	24	-52.12	0.48
A2AKU9;Q8C2Q8;Q91VR2	-1.06	5.76E-05	4.69E-05	30	Atp5c1;Atp5c1;Atp5f1	3	-52.09	0.48
Q99JY9	-1.06	6.05E-05	4.90E-05	60	Actr3	5	-52.03	0.48
Q8C522	-1.05	5.47E-05	4.49E-05	20	Endod1	2	-51.75	0.48
P97351	-1.05	8.82E-18	3.72E-17	430	Rps3a	32	-51.73	0.48
P26638;Q8C483	-1.05	3.60E-11	7.88E-11	180	Sars1;Sars	12	-51.70	0.48
Q91ZW3	-1.05	0.0040232	0.00206143	50	Smarca5	6	-51.54	0.48
Q9D967	-1.05	0.03375891	0.01314612	10	Mdp1	1	-51.54	0.48
Q9JKX6	-1.04	0.00109758	0.00066054	20	Nudt5	2	-51.49	0.49
A0A140T8T4;P51410	-1.04	4.82E-05	4.00E-05	40	Rpl9-ps6;Rpl9	6	-51.39	0.49
Q8CAY6	-1.04	1.42E-13	3.99E-13	100	Acat2	10	-51.33	0.49
Q61048	-1.04	0.03993053	0.01516581	10	Wbp4	2	-51.29	0.49
A0A0A0MQ99;Q8K3D3;V9GXX7	-1.04	0.00451633	0.00227708	10	Swi5	1	-51.21	0.49
O54734	-1.04	0.00289409	0.00154939	30	Ddost	3	-51.20	0.49
E0CY49;Q91YI0	-1.03	0.00683748	0.00327289	30	Asl	4	-51.10	0.49
P56399;Q3U4W8	-1.03	7.58E-07	8.60E-07	120	Usp5	12	-51.02	0.49
P52480	-1.03	1.20E-08	1.77E-08	550	Pkm	43	-51.00	0.49
A0A0R4J099;Q3UY34	-1.03	0.0112915	0.00501003	30	2210016L21Rik;Custo	3	-51.00	0.49

P67778	-1.03	2.37E-09	3.98E-09	160	Phb	16		-50.92	0.49
Q9CQ06	-1.03	0.01127566	0.00500891	20	Mrpl24	2		-50.87	0.49
Q08288	-1.02	6.00E-11	1.29E-10	110	Lyar	11		-50.85	0.49
Q91VR5	-1.02	0.01040694	0.00466761	50	Ddx1	7		-50.74	0.49
Q9QZE5	-1.02	0.04569352	0.01711891	20	Copg1	3		-50.71	0.49
J3QMG3;Q60931	-1.02	0.00107645	0.0006496	20	Vdac3	2		-50.70	0.49
A2APB8	-1.02	2.63E-13	7.13E-13	110	Tpx2	10		-50.65	0.49
G5E8V8;Q9JJT9	-1.02	0.00467156	0.00234463	10	Phax	1		-50.54	0.49
P19253	-1.01	1.45E-18	6.64E-18	260	Rpl13a	19		-50.38	0.50
Q9CQJ8	-1.01	0.00179734	0.00101717	30	Ndufb9	3		-50.37	0.50
P08249	-1.01	6.35E-20	3.39E-19	210	Mdh2	18		-50.36	0.50
F6YU21;Q3TYG6	-1.01	0.00034999	0.00023739	10	Togaram2	1		-50.31	0.50
P16110;Q8C253	-1.01	7.99E-17	3.06E-16	250	Lgals3	14		-50.28	0.50
P35278	-1.01	0.00275802	0.00150149	30	Rab5c	4		-50.22	0.50
A0A0N4SVB8;F6QKK2;Q8VEH3;Q9CQW2	-1.00	0.00629338	0.00304104	20	Arl8b;Arl8a;Arl8a;Arl8	2		-50.13	0.50
Q9CPQ8	-1.00	5.63E-05	4.60E-05	30	Atp5mg	2		-50.06	0.50
Q3TZZ7	1.00	0.00243393	0.00134049	20	Esyt2	2		100.35	2.00
E9PZN0;E9Q3K1;E9Q8I7	1.01	0.02569624	0.01031589	10	Nfxl1	1		100.72	2.01
Q64318	1.01	0.00795613	0.00370912	10	Zeb1	1		101.77	2.02
A0A1B0GX25;Q9Z2V5	1.01	0.02485385	0.0100328	10	Hdac6	1		101.99	2.02
Q922Y1	1.02	3.40E-08	4.69E-08	90	Ubxn1	9		103.39	2.03
F8VQ93;Q8VDD9	1.03	0.00345119	0.0018075	30	Phip	3		103.55	2.04
G3UXH4;G3UZM9;Q8BWW9	1.03	0.00652896	0.00314798	10	Pkn2	1		103.77	2.04
A0A5F8MPQ8;E9PUI5;E9PUK3;Q8K012	1.03	0.02949409	0.01166373	30	Fnbp1l	5		103.86	2.04
Q8VHX6	1.03	3.69E-25	2.98E-24	330	Flnc	34		103.96	2.04
Q3UPH7;S4R189;S4R2U9	1.03	0.04958382	0.01835822	10	Arhgef40	2		104.58	2.05
Q9DAR7	1.04	7.28E-05	5.79E-05	60	Dcps	9		105.15	2.05
D3YW42;G3X927;Q8VC31	1.04	0.00036108	0.00024317	20	Ccdc9	2		105.61	2.06
Q8K4Z3	1.04	0.00568878	0.00279168	30	Naxe	3		105.91	2.06
Q91VM9	1.05	0.00026976	0.00018918	60	Ppa2	7		107.05	2.07
Q8VDP4	1.05	0.00140685	0.00081553	30	Ccar2	3		107.18	2.07
P27661	1.05	0.00015865	0.00011761	100	H2ax	6		107.35	2.07
P60840	1.05	3.13E-07	3.78E-07	60	Ensa	4		107.37	2.07
A0A0R4J073;A0A0R4J183;E9Q577;Q5U5Q9	1.05	0.00147121	0.00084764	10	Uimc1	1		107.71	2.08
Q9JHJ0	1.06	5.11E-22	3.13E-21	220	Tmod3	19		107.88	2.08
A0A0R4J0T8;Q9D8S3	1.06	8.17E-07	9.21E-07	60	Arfgap3	9		108.22	2.08
Q9CU62	1.07	0.00822033	0.00381349	70	Smc1a	12		109.46	2.09
Q8C6E0	1.07	0.0003347	0.00022796	20	Cfap36	3		109.47	2.09
P50396	1.07	0.00116757	0.00069571	20	Gdi1	3		110.36	2.10
A0A0R4J1N9;P40630	1.08	6.63E-10	1.22E-09	60	Tfam	6		111.16	2.11
A3KGL9;F6W687;P09602	1.08	2.32E-10	4.55E-10	190	Hmgn2	11		111.17	2.11
Q9Z1M8	1.08	2.33E-06	2.44E-06	100	Ik	10		111.23	2.11
Q62093	1.08	7.22E-05	5.75E-05	120	Srsf2	10		112.10	2.12
Q80VD1	1.09	0.00599348	0.00291527	10	Fam98b	1		112.25	2.12
O54834	1.09	0.00309574	0.0016395	10	Arhgap6	1		112.57	2.13
A0A286YDF5;Q69ZN7	1.09	1.55E-27	1.60E-26	320	Myof	36		112.72	2.13
E9Q3X0;Q9EQK5	1.09	5.71E-11	1.23E-10	100	Mvp	11		113.35	2.13
Q3UPH1	1.09	0.02182141	0.00895584	10	Prrc1	1		113.51	2.14
Q9QZM0	1.09	0.03113971	0.01226323	50	Ubqln2	5		113.60	2.14
Q9DBG7	1.10	0.00377844	0.00195416	20	Srpra	2		113.96	2.14
A0A0G2JDY4;Q8JZN5	1.10	0.00029787	0.00020605	10	Acad9	1		114.32	2.14
Q8C854	1.11	3.69E-10	6.99E-10	140	Myef2	12		115.72	2.16
A2BH40;E9QAQ7	1.12	3.20E-05	2.75E-05	60	Arid1a	6		116.95	2.17
P0DP26;P0DP27;P0DP28	1.12	4.34E-05	3.65E-05	120	Calm1;Calm2;Calm3	12		117.19	2.17
A2AIM4	1.12	1.28E-16	4.72E-16	90	Tpm2	6		117.66	2.18
D3YV69;P35279	1.13	0.00744632	0.00351602	10	Rab6a	2		118.17	2.18
E0CYV0;E9PWE0;F7D432;P23506	1.13	0.00050957	0.00033105	80	Pcmt1	8		118.37	2.18
D3Z4L9;G3UWJ7;G3UXC6;G3XA62;Q61333	1.13	0.01022368	0.00459476	10	Tnfaip2	1		119.16	2.19
P45952	1.13	1.85E-10	3.67E-10	90	Acadm	11		119.36	2.19
Q8BH43	1.13	4.48E-06	4.48E-06	100	Wasf2	11		119.39	2.19
A0A1Y7VMH3;Q80X41	1.14	0.00030899	0.00021285	30	Vrk1	2		121.03	2.21
F7CVJ5	1.15	6.48E-29	7.66E-28	390	Ahnak2	27		122.07	2.22
A0A0G2JEX1;Q7TPW1	1.15	0.00432227	0.0021926	60	Nexn	9		122.60	2.23
H3BIX4;P97300;Z4YLB7	1.16	0.00267431	0.00145951	30	Nptn	2		123.09	2.23
A0A2C9F2D2;Q07076	1.16	5.31E-07	6.12E-07	70	Anxa7	4		123.70	2.24
Q8BGC4	1.16	2.95E-06	3.05E-06	40	Zadh2	3		123.94	2.24
P81117;Q3UKN6	1.16	3.04E-11	6.79E-11	110	Nucb2	11		123.99	2.24
F7D1J2;Q60855	1.16	0.0010136	0.0006156	10	Ripk1	1		124.06	2.24
Q8BHC9	1.17	0.01905537	0.00793874	10	Fut11	3		124.36	2.24
A0A571BE25;A0A571BEG2;A0A571BEI3;A0A571BGH5;A2A610;P63094;Q6R0H7	1.17	0.0034922	0.00182465	10	Gnas	1		124.60	2.25
Q3UH70;Q9ESU6	1.17	4.11E-08	5.60E-08	60	Brd4	5		124.84	2.25
A0A1D5RMM8;F8VQ28;Q8VI36	1.17	6.80E-10	1.24E-09	60	Pxn	5		124.93	2.25
D3Z124;D3Z6R9;F6QA74;P28352	1.18	0.00011772	8.94E-05	60	Apex1	7		126.08	2.26
Q8BMK4	1.18	6.11E-14	1.78E-13	250	Ckap4	21		126.30	2.26
E9Q9X1;Q91ZU6;S4R1P5	1.18	0.00014341	0.00010703	30	Dst	9		126.35	2.26
Q8VCF0	1.18	1.61E-08	2.32E-08	30	Mavs	3		126.47	2.26
Q9WV55	1.18	6.13E-15	1.97E-14	190	Vapa	15		126.93	2.27
Q8QZS1	1.18	0.00172257	0.00097883	20	Hibch	2		127.13	2.27
A0A1C7CYV0;B1AX58;Q99K51	1.19	5.86E-06	5.76E-06	70	Pls3	7		127.85	2.28
Q8C8U0	1.19	3.99E-06	4.04E-06	70	Ppfibp1	8		128.26	2.28
P01887	1.20	3.39E-06	3.48E-06	30	B2m	3		129.08	2.29
Q9EQC8	1.20	0.00978551	0.00443092	20	Prcc	2		129.39	2.29
P49586	1.20	0.00015727	0.00011685	40	Pcyt1a	4		129.59	2.30
D3Z0X5;D3Z4N0;E9PWB1;Q6PDH0	1.21	2.45E-10	4.81E-10	120	Phldb1	15		130.63	2.31
Q9D6S7	1.21	7.44E-07	8.46E-07	30	Mrrf	4		131.27	2.31
Q80WW9	1.21	0.00123152	0.00072532	10	Ddrgk1	1		132.07	2.32
P43276	1.22	5.69E-14	1.67E-13	270	H1-5	15		132.23	2.32
Q9QXS1	1.22	4.32E-266	1.43E-263	3800	Plec	295		132.28	2.32
Q3TCJ1;Z4YJY0	1.22	0.00793787	0.00370583	20	Abraxas2	2		132.50	2.32
E9PUR6;E9PYV1;E9Q310;E9Q8A6;P06537	1.22	2.62E-05	2.30E-05	30	Nr3c1	3		133.30	2.33
E9Q805;F8VPU2	1.23	8.60E-05	6.72E-05	10	Farp1	1		133.86	2.34
Q7TSV4	1.23	5.35E-05	4.40E-05	30	Pgm2	3		133.91	2.34
Q64511	1.23	4.04E-05	3.43E-05	30	Top2b	5		133.98	2.34
P28667	1.23	0.00529512	0.00261986	30	Marcksl1	3		134.44	2.34
Q62446	1.23	1.11E-23	7.72E-23	420	Fkbp3	26		134.55	2.35
P42125	1.24	0.00067794	0.00042824	80	Eci1	8		136.62	2.37
Q9CW03	1.25	2.19E-06	2.31E-06	80	Smc3	8		137.74	2.38
A0A5H1ZRL3	1.27	0.00118394	0.00070289	40	Nelfb	3		140.38	2.40
Q99K30	1.27	0.00388598	0.0020004	10	Eps8l2	1		141.01	2.41
B1AZ46;Q8BKX1	1.27	3.77E-06	3.84E-06	40	Baiap2	6		141.39	2.41
P35831	1.28	0.00700287	0.00334481	10	Ptpn12	1		142.95	2.43
G3X9J0;S4R1S0;S4R2N4	1.28	0.0363634	0.01400382	40	Sipa1l3	3		143.14	2.43
Q9JLZ6	1.28	0.00269469	0.00146822	10	Hic2	1		143.31	2.43

Q4VA55	1.29	0.04819766	0.01790508	10	Pwwp3b	1	144.10	2.44
A6H644;Q8BG95	1.31	0.00281453	0.00151976	10	Ppp1r12b	2	148.16	2.48
A0A0J9YVJ6;E9Q9A5;G5E8N7;Q9JLV6	1.32	0.00425943	0.0021657	20	Pnkp	2	148.93	2.49
Q9D8S9	1.32	1.09E-18	5.07E-18	70	Bola1	5	149.77	2.50
E9Q0C1	1.32	0.00239525	0.00132359	10	Sh3kbp1	1	149.88	2.50
Q9JIG8	1.32	0.00011645	8.87E-05	10	Praf2	1	150.11	2.50
F2Z456;Q9DCN2	1.33	0.00313769	0.0016551	60	Cyb5r3	6	151.44	2.51
Q3ULB0;S4R1W5	1.33	0.01890807	0.0078873	10	Rbm6	1	151.82	2.52
E9QMI7;E9QMJ1;E9QN63;H3BJY2;H3BKD4;H3BKE6;H3BL41;Q9QWY8	1.34	0.0005905	0.00037879	20	Asap1	4	152.87	2.53
A0A0R4J036;P08553	1.34	0.00169341	0.00096392	20	Nefm	2	152.91	2.53
Q9Z2E1	1.34	9.66E-05	7.47E-05	50	Mbd2	6	153.15	2.53
P37040;Q05DV1	1.35	0.00382936	0.00197895	20	Por	2	155.65	2.56
Q61792	1.36	2.22E-38	4.60E-37	320	Lasp1	18	157.08	2.57
A0A668KL36;Q810U5	1.36	6.12E-08	8.17E-08	50	Ccdc50	5	157.14	2.57
A2APX3;P21460	1.36	5.50E-08	7.38E-08	30	Cst3	3	157.30	2.57
D3YUE7;D6RET7;Q8CB44	1.37	2.97E-05	2.57E-05	20	Gramd4	2	157.78	2.58
P43277	1.37	3.92E-24	2.89E-23	470	H1-3	32	158.22	2.58
P12382	1.39	0.02267312	0.00927664	10	Pfkl	3	162.01	2.62
B1ASX6;O35936	1.39	0.0075635	0.00356119	10	Alox8	1	162.69	2.63
A0A0R4IZX2;A0A0R4J006;A0A3Q4EBU8;A0A3Q4EHM9;Q6NS54;Q9JHL1	1.40	0.02514634	0.01013648	10	Slc9a3r2	1	163.50	2.63
Q60668	1.40	0.00010849	8.30E-05	290	Hnrnpd	18	164.17	2.64
A0A0R4J0J8;A0A1W2P785;Q2KN98	1.40	0.03518165	0.01362003	10	Specc1l	1	164.59	2.65
P51125	1.40	5.08E-25	4.05E-24	410	Cast	27	164.62	2.65
Q99JF8	1.41	8.67E-14	2.48E-13	310	Psip1	22	164.85	2.65
P53986	1.41	4.98E-07	5.78E-07	40	Slc16a1	2	166.28	2.66
P09450	1.42	0.00514045	0.00255287	10	Junb	2	167.01	2.67
E9Q0G1;Q9CQJ6	1.42	0.00555578	0.00274267	20	Denr	3	167.05	2.67
D3YU17;Q8VCM8	1.42	0.00852093	0.00393365	10	Ncln	1	167.76	2.68
A0A0J9YUZ4;P63158	1.42	1.74E-33	2.74E-32	460	Hmgb1	22	168.01	2.68
A2AHX7;A2AHX8;A2AHX9;Q64373;Q9QWX2	1.42	0.00303527	0.00161135	10	Bcl2l1	1	168.31	2.68
Q11011	1.43	0.00011578	8.83E-05	20	Npepps	3	169.58	2.70
Q80VP1	1.44	9.22E-06	8.85E-06	20	Epn1	2	171.38	2.71
Q8BPB5	1.44	1.76E-09	3.03E-09	60	Efemp1	4	171.41	2.71
Q8CJ53	1.44	1.39E-10	2.82E-10	70	Trip10	8	171.50	2.72
Q6NXL1	1.45	0.0349963	0.01358004	10	Sec24d	1	172.51	2.73
D3YWT1;D3Z3N4	1.45	0.03763917	0.01439465	30	Hnrnph3	4	172.94	2.73
Q62470	1.45	7.49E-10	1.35E-09	40	Itga3	4	173.33	2.73
O88952	1.45	1.83E-13	5.06E-13	70	Lin7c	8	173.70	2.74
Q9D809	1.46	6.35E-06	6.21E-06	50	2200002D01Rik	3	174.62	2.75
Q8BPZ8	1.46	0.00122496	0.00072211	10	Abraxas1	1	175.37	2.75
Q91YU6	1.46	0.00595883	0.00290231	20	Lzts2	2	175.45	2.75
Q9R059	1.47	0.00014615	0.00010895	30	Fhl3	3	177.24	2.77
A0A494B8Y8;A0A494BA71;A0A494BA88;Q91XB7	1.48	0.00093584	0.0005747	10	Yif1a	1	178.19	2.78
P52927;Q6NSP9	1.48	7.01E-09	1.10E-08	90	Hmga2	8	179.32	2.79
A0A1C7ZN10;B2RRE2;E9Q405;E9QA74;E9QAX2;K3W4L0;Q9JMH9	1.48	0.02798421	0.01114654	40	Myo18a	5	179.39	2.79
G3UXT7;P56959;Q8CFQ9	1.48	0.02827561	0.01124907	200	Fus	14	179.73	2.80
Q3U9H3;Q61337	1.48	0.00721834	0.00342794	10	Bad	1	179.87	2.80
C0HKE1;C0HKE2;C0HKE3;C0HKE4;C0HKE5;C0HKE6;C0HKE7;C0HKE8;C0HKE9;Q8C	1.49	9.03E-20	4.63E-19	439	H2ac4;H2ac6;H2ac7;H	32	180.27	2.80
Q64191	1.49	4.41E-05	3.70E-05	60	Aga	7	180.33	2.80
Q8CCK0	1.52	0.04680193	0.01747483	20	Macroh2a2	2	186.37	2.86
Q9CWS0	1.53	0.00182997	0.00103365	10	Ddah1	1	187.85	2.88
E9PZ21	1.53	5.36E-05	4.40E-05	30	Rbms1	5	188.50	2.89
P97384	1.54	0.00434599	0.00220126	50	Anxa11	4	189.97	2.90
A0A494B9U1;Q7TSH3	1.54	0.01956083	0.00811358	10	Zfp516;Znf516	1	190.36	2.90
Q8BI72	1.54	5.42E-08	7.29E-08	40	Cdkn2aip	5	191.08	2.91
G5E8C3;Q8BHL4	1.55	0.000103	7.91E-05	10	Gprc5a	1	192.25	2.92
G5E8R8	1.55	2.19E-12	5.54E-12	50	Ubxn7	5	193.34	2.93
A0A0J9YUE9;A0A0J9YUN4;F8WIV5;G3X9G4;P39053;P39054;Q3T9X3;Q3TCR7	1.56	0.00098976	0.00060389	10	Dnm1;Dnm1;Dnm2;D	2	194.46	2.94
Q8BX02	1.56	2.84E-20	1.58E-19	110	Kank2	10	195.32	2.95
J3KMQ5;J3QM81;J3QPB4;Q80UU1	1.57	0.00375237	0.00194221	10	Ankzf1	1	196.42	2.96
A0A338P6P6;D4AFX6;Q6ZQK5	1.57	0.01520426	0.0064895	30	Acap2	2	197.29	2.97
A0A3B2WBE1;A0A3B2WD96;A0A5F8MPE1;A0A5F8MPR1;A7YY80;D0VYV6;Q9W	1.58	1.87E-09	3.19E-09	80	Epb41l3	9	199.37	2.99
Q9D6U8	1.58	1.56E-07	1.98E-07	50	Fam162a	6	199.39	2.99
Q58A65	1.58	1.92E-06	2.05E-06	90	Spag9	6	199.39	2.99
P84104	1.59	5.39E-09	8.56E-09	130	Srsf3	11	200.23	3.00
Q8R550	1.59	1.09E-36	1.95E-35	190	Sh3kbp1	18	200.25	3.00
Q8C166	1.59	4.41E-06	4.41E-06	50	Cpne1	5	200.81	3.01
P43274	1.59	1.06E-08	1.59E-08	130	H1-4	7	200.97	3.01
D3Z5N6;Q61103	1.59	0.0048546	0.00242602	10	Dpf2	2	201.35	3.01
P70202	1.61	7.02E-10	1.28E-09	30	Lxn	2	205.24	3.05
Q3TQP0;Q9CQY2	1.61	0.02271227	0.00928692	10	Gm10767;Ramac	1	206.01	3.06
Q9ESU7	1.61	0.00384759	0.00198674	10	Slc1a5	1	206.04	3.06
Q91XU0	1.62	0.00783252	0.00366699	10	Wrnip1	1	206.35	3.06
Q8VE94	1.62	0.00261985	0.00143216	10	Fam110c	2	206.56	3.07
O55022	1.62	1.67E-22	1.09E-21	150	Pgrmc1	12	206.60	3.07
Q99P72	1.62	1.06E-08	1.59E-08	60	Rtn4	6	207.12	3.07
A0A286YD76	1.62	4.60E-07	5.38E-07	30	Macf1	2	207.27	3.07
Q3U422	1.62	2.42E-07	2.97E-07	80	Ndufv3	9	207.95	3.08
Q99JY1	1.63	0.00566814	0.00278361	10	Tirap	1	209.06	3.09
E9QM38	1.64	3.97E-08	5.45E-08	50	Slc12a2	4	211.48	3.11
E9Q1L5;Q8BUH8	1.64	7.54E-09	1.18E-08	30	Senp7	2	211.86	3.12
Q99J99	1.64	1.43E-08	2.08E-08	50	Mpst	7	212.00	3.12
Q3ULB1;Q921W7	1.66	4.25E-43	1.17E-41	190	Tes	14	215.75	3.16
O70318	1.67	6.84E-37	1.26E-35	410	Epb41l2	37	217.25	3.17
Q3U182	1.67	0.00191245	0.00107474	20	Crtc2	2	218.60	3.19
Q08093	1.67	3.16E-10	6.08E-10	110	Cnn2	10	218.75	3.19
A0A1L1SSX7;F6VG18;F6VTH5;Q9JHQ5	1.68	0.00111798	0.00066978	10	Lztfl1	1	219.40	3.19
A0A1Y7VLX7;E9Q5D6;P69566	1.68	0.00294103	0.00156886	10	Ranbp9	1	220.49	3.20
Q8BP40	1.68	8.89E-09	1.37E-08	20	Acp6	2	221.25	3.21
Q8C3W1	1.69	3.62E-09	5.87E-09	100	NaN	11	221.85	3.22
A0A0U1RNK7;A2A9M4;A2A9M5;E9PX48;Q8R1A4	1.69	2.74E-08	3.86E-08	50	Dock7	5	221.98	3.22
F7A465	1.69	1.12E-06	1.23E-06	40	Hnrnpd	3	223.02	3.23
E0CYE3;Q3TMW1	1.70	0.04399745	0.01655845	10	Ccdc102a	1	225.55	3.26
F2Z3X3;Q8R3V6	1.71	0.00133703	0.00078052	10	Cuedc1	1	226.13	3.26
Q811P8	1.72	8.53E-07	9.61E-07	10	Arhgap32	1	228.74	3.29
P17095	1.73	1.59E-11	3.72E-11	110	Hmga1	11	230.85	3.31
P11688	1.73	0.04717673	0.01757512	10	Itga5	2	232.20	3.32
E9Q7B0;Q60715	1.73	3.98E-09	6.39E-09	70	P4ha1	8	232.37	3.32
Q501J7	1.75	0.00029583	0.00020485	40	Phactr4	3	235.20	3.35
A0A0R4J1E3;F7CPL2;Q9QXS6	1.75	0.00144133	0.0008326	10	Dbn1	1	235.96	3.36
O70475	1.75	3.64E-10	6.92E-10	100	Ugdh	12	236.51	3.37
E9QJU8;Q8CG79	1.76	0.00613081	0.00297551	10	Trp53bp2;Tp53bp2	3	239.52	3.40
A0A338P6G6;A0A384DV79	1.77	6.46E-08	8.60E-08	20	Hmga1	1	241.25	3.41

Q9DCL8	1.78	7.26E-20	3.79E-19	140	Ppp1r2	11	243.31	3.43
Q8VCQ8	1.79	3.62E-103	7.98E-101	650	Cald1	44	246.43	3.46
Q60929	1.80	0.04084174	0.01547638	10	Mef2a	1	247.13	3.47
Q91Z78;Q9Z2F7	1.80	0.00385644	0.00198674	10	Bnip3l	1	249.40	3.49
Q8C0E3	1.84	1.18E-08	1.75E-08	20	Trim47	2	256.87	3.57
E9QNX9;Q60751	1.84	9.06E-09	1.39E-08	20	Igf1r	3	258.74	3.59
Q5I1X5	1.84	0.0039413	0.00202573	20	Ppp1r13l	2	259.14	3.59
Q9D281	1.85	3.45E-06	3.54E-06	20	Fam114a1	3	259.96	3.60
Q63810	1.85	0.03321641	0.01298068	10	Ppp3r1	1	260.27	3.60
E9Q6R7	1.85	2.67E-13	7.23E-13	80	Utrn	14	260.54	3.61
E9Q616	1.86	0	0	7140	Ahnak	439	261.83	3.62
Q91VH2	1.86	2.05E-13	5.65E-13	60	Snx9	6	263.27	3.63
P10922	1.86	2.08E-24	1.55E-23	360	H1-0	23	264.25	3.64
A0A1D5RLY6;Q8C052	1.87	2.29E-06	2.41E-06	70	Map1s	9	266.36	3.66
Q69Z38	1.87	6.26E-10	1.16E-09	40	Peak1	6	266.76	3.67
A0A1Y7VJN9;A0A1Y7VL34;Q8CFR5;Q9D2N4	1.88	3.91E-06	3.97E-06	20	Dtna	2	268.59	3.69
Q8K297	1.88	1.35E-08	1.97E-08	50	Colgalt1	5	268.90	3.69
A2AIH8;Q9D711	1.89	0.00054237	0.00035064	20	Pir	2	270.22	3.70
P63328	1.90	3.44E-07	4.11E-07	20	Ppp3ca	2	274.21	3.74
G5E829	1.90	4.64E-05	3.87E-05	50	Atp2b1	4	274.47	3.74
P29533;Q3UPN1	1.91	4.48E-07	5.26E-07	20	Vcam1	2	274.66	3.75
A2AKA9;Q8K2F0	1.91	0.00206255	0.00115322	20	Brd3	4	275.66	3.76
Q64727	1.93	5.34E-71	4.42E-69	470	Vcl	42	279.95	3.80
P57787	1.93	0.0008955	0.00055404	10	Slc16a3	1	280.61	3.81
Q9D832	1.94	9.65E-09	1.47E-08	60	Dnajb4	5	283.69	3.84
O55125	1.94	0.02012342	0.00831048	10	Nipsnap1	1	284.22	3.84
P23780	1.94	0.00034907	0.00023701	40	Glb1	4	284.57	3.85
Q61205	1.94	1.58E-07	2.00E-07	50	Pafah1b3	5	284.61	3.85
A0A0R4J196;E9PUD1;F6WNT4;F8WH31	1.94	0.00345793	0.00180818	10	Spag9	1	284.95	3.85
Q60770	1.96	0.00958148	0.00435941	10	Stxbp3	1	288.12	3.88
Q6GSS7	1.96	2.20E-13	6.02E-13	100	Hist2h2aa1	6	288.42	3.88
P08207	1.97	8.27E-05	6.50E-05	100	S100a10	7	290.60	3.91
B1AV75;Q3UJP5	1.99	0.00267847	0.00146058	10	2610301B20Rik;	1	296.63	3.97
Q9WTX2	2.00	0.01704186	0.00718123	10	Prkra	1	298.62	3.99
A0A338P6F6	2.01	0.01246268	0.00542426	10	Hmga1	1	302.70	4.03
B9EHJ3	2.01	3.09E-58	1.57E-56	480	Tjp1	36	303.43	4.03
E9Q1S3;Q01405	2.01	0.00433456	0.00219715	20	Sec23a	2	303.67	4.04
P61166	2.02	0.00659406	0.00316783	10	Tmem258	1	305.96	4.06
Q80W68	2.03	0.00261908	0.00143216	10	Kirrel1	2	307.16	4.07
Q9DBX1	2.03	0.00048379	0.00031584	10	Rgcc	1	309.10	4.09
P83741	2.04	9.27E-05	7.19E-05	100	Wnk1	11	310.55	4.11
A0A0R4J0G0;A0A2I3BQL9;Q8BH04	2.05	0.0002685	0.00018849	30	Pck2	5	312.87	4.13
Q8BFW7	2.05	4.47E-40	1.06E-38	230	Lpp	18	313.82	4.14
P15864	2.06	0.00012875	9.71E-05	150	H1-2	9	316.17	4.16
A2AN61;F6S5B6;F7D5G9;Q3UUV0;Q8VEH2	2.06	5.00E-05	4.13E-05	30	Ciz1	3	318.21	4.18
Q8K094	2.07	0.00010227	7.86E-05	30	Pvr	4	320.33	4.20
Q61576	2.08	3.79E-05	3.23E-05	60	Fkbp10	6	322.17	4.22
D6REG4	2.08	4.42E-14	1.32E-13	20	Grhpr	2	323.96	4.24
Q4VA93	2.09	0.00975063	0.00442118	20	Prkca	2	324.71	4.25
Q6P9R2	2.13	0.00785356	0.00367165	30	Oxsr1	5	337.57	4.38
E9Q5E0;E9QKT0;Q63943;Q921S6	2.14	0.04463531	0.01677943	10	Mef2d	1	341.40	4.41
P47739	2.15	7.46E-25	5.88E-24	160	Aldh3a1	14	345.27	4.45
Q920P5	2.16	0.00297389	0.0015813	30	Ak5	3	346.25	4.46
D3YW86;D3Z0V7;E9QLZ1;H3BLI9;P62500	2.16	4.10E-06	4.14E-06	70	Tsc22d1	5	348.08	4.48
Q9EQP2	2.17	5.34E-17	2.07E-16	80	Ehd4	8	348.87	4.49
P48755	2.17	0.02481661	0.01002967	10	Fosl1	1	350.37	4.50
Q6P9Q4	2.18	8.34E-08	1.09E-07	30	Fhod1	3	353.30	4.53
E9QN70;P02469	2.18	8.37E-31	1.13E-29	210	Lamb1	26	353.64	4.54
Q925B0	2.19	0.0027761	0.00151009	20	Pawr	3	355.10	4.55
A2AEW0;A2AEW1;Q64092;Z4YLF7	2.20	0.01235472	0.00539145	10	Tfe3	1	358.95	4.59
Q8BUN5	2.22	0.00083976	0.00052396	30	Smad3	2	366.34	4.66
A0A0R4J0I9;Q91ZX7	2.24	5.57E-14	1.64E-13	40	Lrp1	4	373.51	4.74
P15306	2.25	0.00025213	0.00017871	10	Thbd	1	376.58	4.77
A0A2R8VK26;D3Z4A3;D3Z4S9;D3Z606;D3Z6P2;Q6NZQ6	2.26	0.01307176	0.00565958	10	Zfp740;Zfp740;Zfp74(	1	379.31	4.79
A0A1L1SS82	2.27	0.0015112	0.00086917	10	Ccdc7a	1	381.68	4.82
A0A494BA29;Q6IRU7	2.30	1.90E-06	2.04E-06	10	Cep78	1	391.06	4.91
P06797	2.30	1.43E-22	9.58E-22	90	Ctsl	9	392.03	4.92
Q91YN9	2.30	4.95E-05	4.10E-05	10	Bag2	1	392.81	4.93
Q60772	2.31	0.00058428	0.00037553	30	Cdkn2c	3	396.19	4.96
Q4FZC9	2.32	0.00027699	0.00019363	20	Syne3	4	400.09	5.00
Q00560;V9GX00	2.32	0.00541361	0.00267648	10	Il6st	1	400.72	5.01
Q8K3X4	2.35	8.47E-10	1.52E-09	60	Irf2bpl	6	409.26	5.09
Q9R0Y5	2.39	3.74E-06	3.83E-06	50	Ak1	8	424.79	5.25
A0A494BB84;Q3U4F0;Q91V61	2.40	7.26E-16	2.46E-15	40	Sfxn3	4	428.53	5.29
Q99L04	2.41	7.15E-07	8.18E-07	20	Dhrs1	2	429.72	5.30
E9PZD2;Q3TN34	2.42	1.20E-05	1.12E-05	20	Micall2	4	435.92	5.36
P81122	2.43	1.37E-05	1.28E-05	40	Irs2	6	439.65	5.40
G5E897	2.44	9.29E-05	7.20E-05	20	Poglut3	1	441.25	5.41
A0A0R4J138;P50429	2.44	2.24E-13	6.10E-13	70	Arsb	5	444.51	5.45
Q9JMG7	2.45	5.16E-09	8.24E-09	40	Hdgfl3	3	445.76	5.46
D3YXR5;D3Z3A6;Q60953	2.47	2.07E-09	3.52E-09	20	Pml	5	453.43	5.53
A0A3B2WD42	2.47	0.02283164	0.00932421	10	Gm4356	1	455.68	5.56
P52651;Q3UJH4;V9GXJ4	2.48	0.00067206	0.00042493	10	Rhox5	1	459.76	5.60
D3YTT6;D3Z194;F8WH20;Q9DBS9	2.49	1.76E-09	3.03E-09	50	Osbpl3	9	459.84	5.60
P55200	2.50	1.08E-09	1.91E-09	40	Kmt2a	5	467.38	5.67
O54724	2.51	4.67E-62	2.81E-60	400	Cavin1	26	469.60	5.70
Q91XC8	2.53	7.63E-13	2.00E-12	80	Dap	4	476.91	5.77
D3Z7U4	2.53	3.43E-09	5.59E-09	30	Mecp2	4	478.18	5.78
Q80U35	2.53	2.67E-08	3.77E-08	30	Arhgef17	3	478.71	5.79
Q91X84	2.54	0.01223807	0.00535955	10	Crtc3	1	481.74	5.82
A0A0G2JGD2;P07091	2.54	3.79E-33	5.71E-32	210	S100a4	16	482.32	5.82
Q5SF07	2.54	1.10E-24	8.50E-24	100	Igf2bp2	10	482.89	5.83
Q60963	2.55	0.00062007	0.0003966	10	Pla2g7	1	483.83	5.84
A0A0R4J0J1;A0A0R4J1D0;P59108;Q0VE82;Q1RLL3;Q3UYN2;Q8BLR2;Q8BT60;Q8J	2.60	0.00345491	0.00180802	10	Cpne9;Cpne2;Cpne2;(	1	508.26	6.08
Q9QYZ8	2.61	0.00519478	0.00257599	10	Dkk2	1	509.19	6.09
O70326	2.61	2.24E-05	1.98E-05	10	Grem1	1	509.60	6.10
Q9D7J9	2.61	0.00010791	8.27E-05	10	Echdc3	1	510.46	6.10
P21550	2.61	0.00117192	0.00069705	20	Eno3	4	510.81	6.11
A0A0G2JG52;E9PVP1	2.61	4.25E-06	4.27E-06	40	Crybg1	5	511.01	6.11
Q8BJS4	2.62	7.09E-05	5.65E-05	20	Sun2	4	514.99	6.15
Q99KC8	2.62	1.00E-09	1.79E-09	20	Vwa5a	4	516.63	6.17
Q64261	2.63	0.00021464	0.00015395	10	Cdk6	1	519.64	6.20
P62748	2.63	8.87E-07	9.96E-07	20	Hpcal1	3	519.94	6.20

H3BKG0;P49817	2.65	9.72E-15	3.06E-14	60	Cav1	5	529.52	6.30
Q9Z2D6	2.68	0.00144116	0.0008326	20	Mecp2	2	539.96	6.40
A0A2C9F2A2;Q921Q7	2.68	3.55E-13	9.47E-13	80	Rin1	9	542.12	6.42
A2AJT3;A2AJT4;A2AJT5	2.68	0.02421377	0.00984614	10	Pnisr	1	542.93	6.43
Q810Q5	2.69	0.00035775	0.00024117	20	Nmes1	2	543.55	6.44
A2AQR0;Q64521	2.70	0.01239912	0.00540681	30	Gpd2	6	548.64	6.49
Q8BH64	2.72	5.93E-35	1.01E-33	110	Ehd2	15	557.40	6.57
Q05769	2.74	0.00140257	0.00081376	40	Ptgs2	4	567.12	6.67
H3BJ97;Q99JR5	2.76	0.00020074	0.00014476	10	Tinagl1	3	579.46	6.79
G5E8E1	2.78	2.15E-05	1.90E-05	30	Lrrfip1	4	585.04	6.85
Q9QUH0	2.80	0.00296424	0.0015787	10	Glrx	2	598.48	6.98
A2AFL3;P70699	2.82	3.99E-08	5.46E-08	40	Gaa	3	605.90	7.06
A2AWS5;B7ZDF4;B7ZDF5;B7ZDF7;Q3UJF1;Q9Z2V6	2.87	0.0050308	0.00250594	10	Hdac5	1	629.17	7.29
Q922B1	2.88	1.47E-06	1.60E-06	30	Macrod1	2	636.53	7.37
P82349	2.89	0.03461628	0.01345554	10	Sgcb	1	641.86	7.42
P20065	2.91	2.02E-24	1.52E-23	90	Tmsb4x	4	653.88	7.54
Q9Z0L0	2.94	1.13E-06	1.25E-06	20	Tpbg	2	665.63	7.66
A2AMM0	2.95	1.34E-09	2.35E-09	70	Cavin4	9	673.53	7.74
Q63918	3.00	2.17E-15	7.14E-15	190	Cavin2	19	697.48	7.97
Q9WTI7	3.00	0.00014684	0.00010935	40	Myo1c	8	698.40	7.98
G3UYU3;P14719	3.01	0.0002062	0.00014854	10	Il1rl1	1	704.15	8.04
D3YUS4;Q9DD03	3.01	0.00844804	0.00390545	10	Rab13	1	706.33	8.06
Q8C180	3.01	6.04E-09	9.49E-09	30	Frs2	3	706.61	8.07
D2DFA9;Q923Z0	3.02	5.56E-05	4.55E-05	10	Gprc5b	1	709.10	8.09
Q9CXS4	3.03	6.53E-05	5.26E-05	10	Cenpv	1	715.42	8.15
Q5DTX6	3.05	0.00311675	0.00164685	10	Jcad	1	725.98	8.26
Q8K1I7	3.11	1.48E-05	1.36E-05	20	Wipf1	3	761.84	8.62
A6PWS5	3.16	4.21E-10	7.94E-10	40	Gsn	2	795.33	8.95
F6ZKZ3;Q9R112	3.20	0.00103632	0.00062767	30	Sqor	4	820.22	9.20
O35640;Q921D0	3.22	0.00031224	0.00021464	30	Anxa8	4	833.88	9.34
Q52KR3	3.25	6.14E-05	4.97E-05	30	Prune2	3	852.99	9.53
Q8BJH1	3.26	3.82E-19	1.88E-18	70	Zc2hc1a	6	857.83	9.58
P56389	3.29	0.00066825	0.00042333	10	Cda	1	875.07	9.75
P99025	3.33	0.00173831	0.00098693	10	Gchfr	1	907.67	10.08
A0A140T8J4	3.38	5.37E-24	3.82E-23	90	Hebp1	7	937.58	10.38
A0A087WR50;A0A087WS56;A0A087WSN6;B7ZNJ1;B9EHT6;P11276;Q3UHL6	3.46	4.28E-18	1.88E-17	90	Fn1	12	996.90	10.97
P15066	3.47	0.00297337	0.0015813	10	Jund	1	1009.27	11.09
P23242	3.48	2.54E-09	4.24E-09	30	Gja1	2	1013.31	11.13
A0A1B0GS91;E9Q3Z5;Q8K4L2;Q8K4L3	3.54	0.00235731	0.00130589	10	Svil	1	1066.13	11.66
P13020	3.54	1.55E-71	1.47E-69	460	Gsn	35	1066.82	11.67
D3Z4J9;D6RII3;Q05BC3	3.58	7.89E-11	1.66E-10	30	Eml1	3	1093.81	11.94
F8WIP8;P10923	3.60	1.38E-33	2.23E-32	130	Spp1	10	1112.23	12.12
D3Z0J2	3.61	4.20E-06	4.23E-06	30	Cav1	2	1119.31	12.19
A0A3Q4EGK3;Q8CG19	3.62	1.50E-22	9.94E-22	90	Ltbp1	8	1126.22	12.26
Q61001	3.63	1.75E-08	2.52E-08	20	Lama5	7	1138.15	12.38
Q8K124	3.63	2.14E-14	6.53E-14	50	Plekho2	6	1138.33	12.38
A0A0G2JDY6	3.64	0.00157475	0.0009018	10	Nexn	1	1143.72	12.44
Q91VJ2	3.68	4.09E-31	5.64E-30	100	Cavin3	8	1180.47	12.80
A0A1B0GR85	3.68	0.00045853	0.00030054	30	2900026A02Rik	4	1181.13	12.81
E9Q4X2	3.69	0.01846201	0.00773045	10	Uggt2	1	1188.94	12.89
G3X9Q1;Q3TZS3;Q61738	3.74	1.85E-05	1.67E-05	20	Itga7	4	1232.85	13.33
A2AUR3;A2AUR5;Q60866	3.75	0.00089198	0.00055238	10	Pter	1	1244.03	13.44
P47880	3.75	0.00121638	0.00071832	10	Igfbp6	1	1248.74	13.49
Q8BM75	3.82	1.87E-08	2.68E-08	30	Arid5b	3	1312.82	14.13
Q8R3G9	3.84	0.00484756	0.00242561	10	Tspan8	1	1332.04	14.32
P28650	3.84	6.73E-09	1.05E-08	50	Adss1	9	1333.60	14.34
Q9CZH7	3.96	2.17E-06	2.29E-06	20	Mxra7	3	1460.66	15.61
F8VQJ3;P02468	4.02	2.09E-50	7.68E-49	220	Lamc1	22	1525.66	16.26
P22437	4.11	5.39E-06	5.33E-06	30	Ptgs1	3	1623.90	17.24
B1B0C7;E9PZ16;Q05793	4.21	2.61E-07	3.18E-07	20	Hspg2	5	1756.40	18.56
Q60793	4.24	0.00353981	0.0018473	20	Klf4	2	1786.51	18.87
A0A2R8VHQ0;Q8CGB6	4.26	3.05E-09	5.02E-09	30	Tns2	4	1811.76	19.12
Q8R1G6	4.58	3.17E-37	6.35E-36	130	Pdlim2	13	2291.19	23.91
Q8R138	4.63	0.00205668	0.00115091	10	Tmem119	2	2379.87	24.80
P08122	5.11	2.71E-11	6.14E-11	30	Col4a2	3	3360.77	34.61
